# Supplementary material for: miR-6077 promotes cisplatin/pemetrexed resistance in lung adenocarcinoma via CDKN1A/cell cycle arrest and KEAP1/ferroptosis pathways
Source: Mol Ther Nucleic Acids. 2022 Mar 28;28:366–86. doi: 10.1016/j.omtn.2022.03.020 (PMC9035384; doi:10.1016/j.omtn.2022.03.020)
Supplement: Document S1. Figures S1–S7 and Tables S1–S5 [file mmc1.pdf]

OMTN, Volume *28*

Supplemental information

miR-6077 promotes cisplatin/pemetrexed resistance in lung adenocarcinoma via CDKN1A/ cell cycle arrest and KEAP1/ferroptosis pathways

Guoshu Bi, Jiaqi Liang, Mengnan Zhao, Huan Zhang, Xing Jin, Tao Lu, Yuansheng Zheng, Yunyi Bian, Zhencong Chen, Yiwei Huang, Valeria Besskaya, Cheng Zhan, Qun Wang, and Lijie Tan

Supplementary Table 1. Oligonucleotides

Name	Source	Sequence
miR-NC1 mimic<br>(micrON mimic NC#19)	RiboBio	Sense: 5’-3’:<br>UUUGUACUACACAAAAGUACUG<br>Antisense: 3’-5’:<br>AAACAUGAUGUGUUUUCAUGAC
miR-NC2 mimic<br>(micrON mimic NC#22)	RiboBio	Sense: 5’-3’:<br>UCACAACCUCCUAGAAAGAGUAGA<br>Antisense: 3’-5’:<br>AGUGUUGGAGGAUCUUUCUCAUCU
hsa-miR-6077 mimic<br>(Purchased from)	RiboBio	Sense: 5’-3’:<br>GGGAAGAGCUGUACGGCCUUC<br>Antisense: 3’-5’:<br>CCCUUCUCGACAUGCCGGAAG
hsa-miR-3678-3p mimic	RiboBio	Sense: 5’-3’:<br>CUGCAGAGUUUGUACGGACCGG<br>Antisense: 3’-5’:<br>GACGUCUCAAACAUGCCUGGCC
hsa-miR-3678-5p mimic	RiboBio	Sense: 5’-3’:<br>UCCGUACAAACUCUGCUGUG<br>Antisense: 3’-5’:<br>AGGCAUGUUUGAGACGACAC
hsa-miR-7702 mimic	RiboBio	Sense: 5’-3’:<br>CUUAGACUGCCAGACUCCCUGA<br>Antisense: 3’-5’:<br>GAAUCUGACGGUCUGAGGGACU
hsa-miR-6855-5p mimic	RiboBio	Sense: 5’-3’:<br>UUGGGGUUUGGGGUGCAGACAUUG<br>C<br>Antisense: 3’-5’:<br>AACCCCAAACCCCACGUCUGUAACG
hsa-miR-548ak mimic	RiboBio	Sense: 5’-3’:<br>AAAAGUAACUGCGGUUUUUGA<br>Antisense: 3’-5’:<br>UUUUCAUUGACGCCAAAAACU
miR-NC1 inhibitor<br>(micrOFF NC#19)	RiboBio	Antisense: 3’-5’:<br>AAACAUGAUGUGUUUUCAUGAC
miR-NC2 inhibitor<br>(micrOFF NC#22)	RiboBio	Antisense: 3’-5’:<br>AGUGUUGGAGGAUCUUUCUCAUCU
hsa-miR-6077 inhibitor	RiboBio	Sense: 5’-3’:<br>GGGAAGAGCUGUACGGCCUUC<br>Antisense: 3’-5’:<br>CCCUUCUCGACAUGCCGGAAG

Supplementary Table 2. Primers used for PCR and qPCR

Gene	Source	Sequence
CDKN1A	Sangon Biotech	F: TGTCCGTCAGAACCCATGC<br>R: AAAGTCGAAGTTCCATCGCTC
KEAP1 (qPCR)	Sangon Biotech	F: CTGGAGGATCATACCAAGCAGG<br>R: GGATACCCTCAATGGACACCAC
KEAP1 (PCR)	Sangon Biotech	F: ACTCGTTGACGCCGAACTTCC<br>R: GGGTTGTAAGCCTCCAGGTAGC
PTGS2	Sangon Biotech	F: CTGGCGCTCAGCCATACAG<br>R: CGCACTTATACTGGTCAAATCCC
GAPDH	Sangon Biotech	F: AGAAGGCTGGGGCTCATTTG<br>R: AGGGGCCATCCACAGTCTTC
GMDS-AS1	Sangon Biotech	F: AATGCTTTGAGGCCAAGCTA<br>R: TGGGTTCATAAGGGTTGCAT
LINC01128	Sangon Biotech	F: CAGAGGAGCTACGAAGGGAG<br>R: CTGTGGAATCGCTTGGTACG
LINC00174	Sangon Biotech	F: AACCTCTCAGGCTCAAGCAGTC<br>R: GGTCACTTCGTTTCGCAGTCAG
LINC01278	Sangon Biotech	F: AAGAAAGCTTCTGTGCCCCC<br>R: AGCTCAGCAGGCAGAATTGA
RALY-AS1	Sangon Biotech	F: AAGGAACGTGCCACCTAGAC<br>R: GGGGAAGCGATGTGAGTCAT
U6	TIANGEN	F: CTCGCTTCGGCAGCACA<br>R: AACGCTTCACGAATTTGCGT
miR-6077	TIANGEN	F: AACAAGGGGAAGAGCTGTACGG<br>R: CAGTGCAGGGTCCGAGGT

Supplementary Table 3. Characteristics of the 31 NSCLC patients included in this study

ID	Age	Gender	T stage	N stage	Location	Cohort	Group
Patient 1	59	M	2	2	R	A	Resistant
Patient 2	37	M	2	0	R	A	Resistant
Patient 3	58	M	3	0	L	A	Sensitive
Patient 4	49	M	2	0	R	A	Resistant
Patient 5	53	M	1	0	R	A	Sensitive
Patient 6	67	M	2	0	R	A	Sensitive
Patient 7	68	M	2	2	R	A	Resistant
Patient 8	60	W	2	2	L	A	Sensitive
Patient 9	61	M	2	0	R	A	Sensitive
Patient 10	65	M	3	2	L	A	Resistant
Patient 11	59	M	2	0	R	A	Resistant
Patient 12	56	M	2	1	L	A	Sensitive
Patient 13	54	M	2	1	R	A	Sensitive
Patient 14	57	W	3	0	L	A	Sensitive
Patient 15	62	W	2	1	L	A	Resistant
Patient 16	56	M	2	1	R	A	Resistant
Patient 17	62	W	3	1	R	A	Sensitive
Patient 18	59	M	2	1	R	A	Sensitive
Patient 19	60	W	2	0	L	A	Resistant
Patient 20	63	W	3	0	R	A	Sensitive
Patient 21	70	M	3	1	R	B	Treated
Patient 22	60	W	2	2	R	B	Treated
Patient 23	50	M	2	3	L	B	Treated
Patient 24	58	M	1	2	R	B	Treated
Patient 25	66	W	2	2	R	B	Untreated
Patient 26	52	W	2	1	L	B	Untreated
Patient 27	74	M	2	2	R	B	Untreated
Patient 28	47	W	3	2	R	B	Untreated
Patient 29	82	M	2	0	L	PDX #1	Untreated
Patient 30	60	M	2	1	L	PDX #2	Untreated
Patient 31	63	W	2	1	R	PDX #3	Untreated

Supplementary Table 4. Antibodies and dyes used for western blotting and flow cytometry

Protein	Source	Dilution
CDKN1A	Abways, #CY5088	1:1000
KEAP1	Absin, #abs131362	1:1000
TUBULIN	Beyotimes, #AF1216	1:1000
MAP3K1	Santa Cruz Biotechnology (USA), #sc-17820	1:500
FOXP4	Santa Cruz Biotechnology (USA), #sc-390892	1:1000
CREN1	Abways, #CY5426	1:1000
KLF6	Santa Cruz Biotechnology (USA), #sc-365633	1:1000
KLF7	Sigma-Aldrich (USA), #HPA030490	1:2000
VEGFA	Abways, #CY5096	1:500
ACTIN	Beyotime, #AF0003	1:1000
CDK1	Abways, #AB3187	1:100
CDK1-Thr161	Abways, #CY6096	1:500
NRF2	Absin, #abs120634	1:500
SLC7A11	Affinity, #DF12509	1:1000
NQO1	Abways, #CY6710	1:1000
AGO2	Abways, #CY6652	1:500
Dyes	Source	Conjugation
Zombie Aqua Fixable Viability Kit	BioLegend (San Diego, USA), #423101	BV510
EPCAM	BioLegend, #369809	APC
CD45	BioLegend, #368531	PE/Cyanine7
FOLR1	R & D Systems (Minneapolis, USA), #FAB5646P	PE

Supplementary Table 5. Sequences of sgRNA, repair template, and the siRNAs used in HDR

Name	Source	Sequence
sgRNA	Sangon Biotech	CTGTCGGAAGTAGCAGCCCG
Repair template	Sangon Biotech	TGCAGATGCAGCTGCAGAAGTGCGAGATCCTGCAGTCCGACTCCCGCTGCAAGGACTACCTGGTCAAGATCTTCGAGGAGCTCACCCTGCACAAGCCCACGCAGGTGATGCCCTGCCGGGCCCCTAAGGTGGGCCGCCTGATCTACACCGCGGGAGGATATTTCAGGCAATCGCTCAGCTACCTGGAGGCTTACAACCCCAGTGACGGCACCTGGCTCCGGTTGGCGGACCTGCAGGTGCCGCGGAGCGGCCTGGCCGGCTGCGTGGTGGGCGGGCTGTTGTACGCCGTGGGCGGCAGGAACAACTCGCCCGACGGCAAC
siKu70-1	RiboBio	UUCAGGUGACUCCUCCAGGTT
siKu70-2	RiboBio	UUCUCUUGGUAACUUUCCCTT
siXRCC4-1	RiboBio	AUAUGUUGGUGAACUGAGATT
siXRCC4-2	RiboBio	UCUUGGGACAGAACCUAAATT
siLig4-1	RiboBio	CGACCUUUUAGACUCAAUUTT
siLig4-2	RiboBio	GCUAGAUGGUGAACGUAUGTT

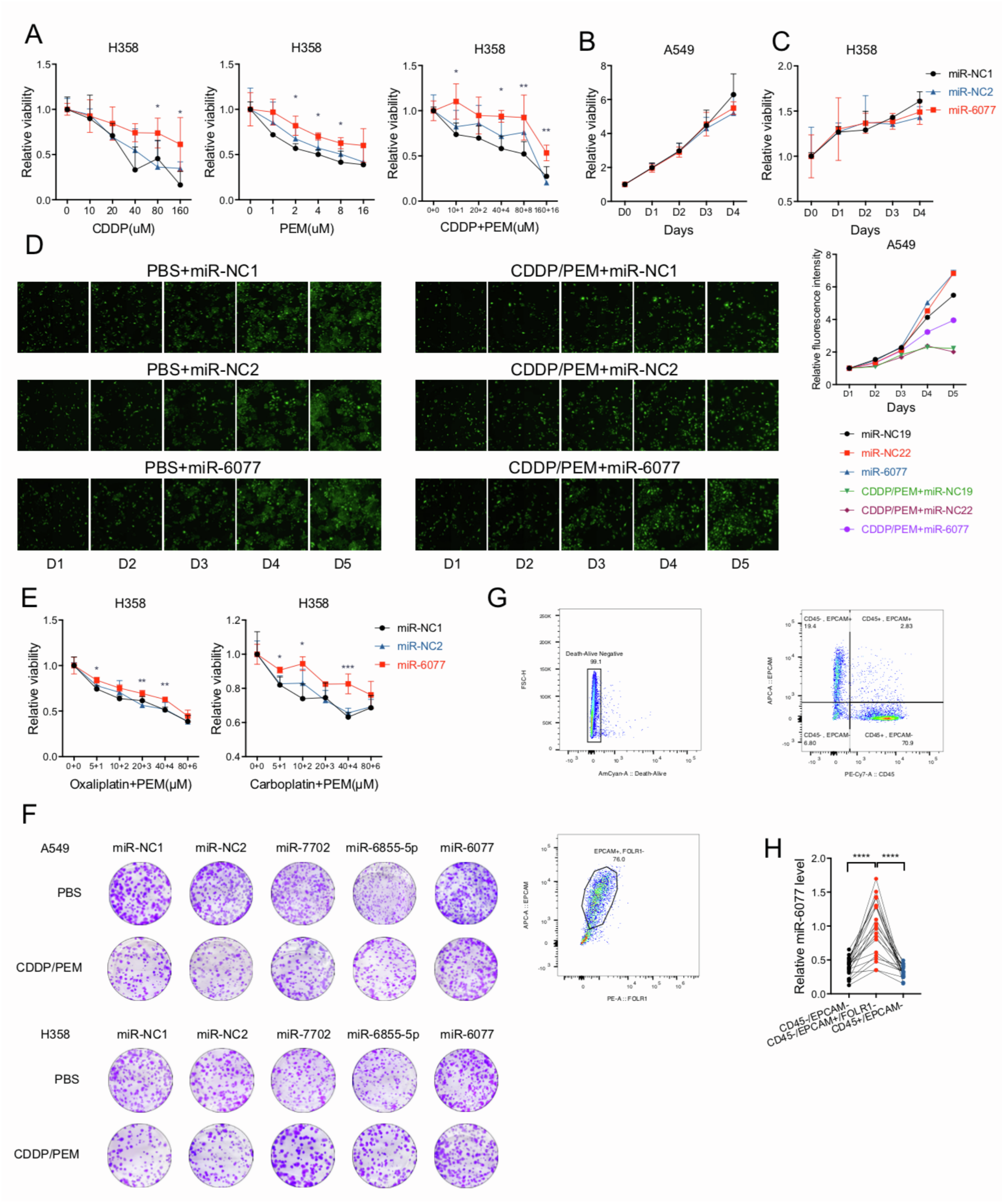

Supplementary Figure 1. Ectopic overexpression of miR-6077 confers resistance to CDDP/PEM in LUAD. **(A)** Dose-toxicity curves showing the cell viability of H358 cells transfected with miR-NC1, miR-NC2, and miR-6077 upon CDDP or PEM treatments at the indicated concentrations for 48 h respectively. **(B, C)** Growth curves showing the proliferating rates of A549 **(B)** and H358 cells **(C)** transfected with miR-NC1, miR-NC2, and miR-6077. **(D)** High-Content Analysis and quantification of GFP-

overexpressing A549 cells transfected with miR-NC1, miR-NC2, and miR-6077 shows the dynamic change of cell proliferation in five days in the presence of PBS or CDDP (10 μM)/PEM (1 μM). (bottom). **(E)** Viability of H358 cells transfected with miR-NC1, miR-NC2, and miR-6077 upon treatments with PEM and oxaliplatin or carboplatin at the indicated concentrations for 48 h respectively. **(F)** Colony formation ability of A549 and H358 cells transfected with different miRNAs upon treatments with PBS or CDDP /PEM for 14 days. **(G)** LUAD patients' surgical specimen were dissected and lymphocytes (CD45+/EPCAM-), tumor cells (CD45-/EPCAM+/FOLR-), other non-tumoral cells (CD45-/EPCAM-), and were identified and sorted by flow cytometry according to the expression of marker genes. **(H)** Relative expression levels of miR-6077 in different cell types. Data are presented as mean ± s.d., n = 3 independent repeats. Unpaired, two-tailed t-test; *$p < 0.05$, **$p < 0.01$, ***$p < 0.001$, ****$p < 0.0001$, ns, not significant.

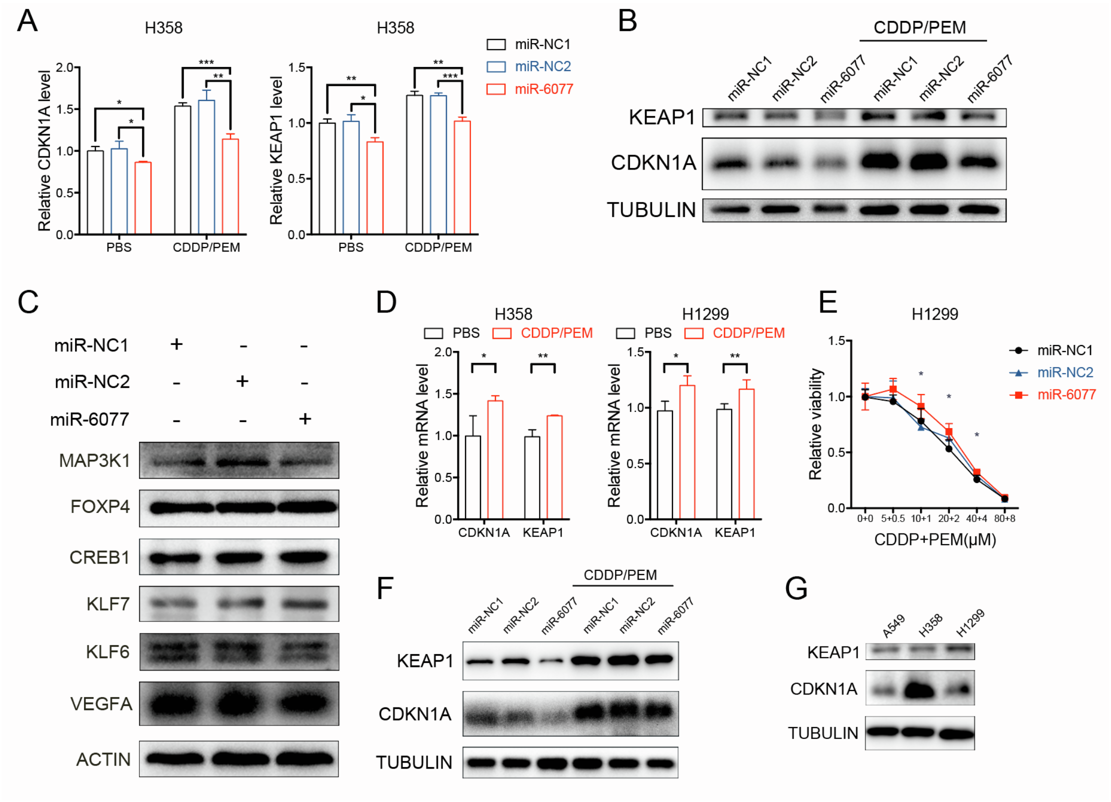

Supplementary Figure 2. CDKN1A and KEAP1 are direct targets of miR-6077 in different LUAD cell lines. **(A, B)** qRT-PCR and western blotting assays showing the mRNA **(A)** and protein **(B)** levels of CDKN1A and KEAP1 in H358 pre-transfected with miR-NC1, miR-NC2, and miR-6077 after PBS or CDDP (20 μM)/PEM (2 μM) treatments for 48 h. **(C)** The protein levels of other six potential downstream targets of miR-6077 in A549 cells transfected with miR-NC1, miR-NC2, and miR-6077. **(D)** qRT-PCR showing the relative expression levels of CDKN1A and KEAP1 in H358 and H1299 cells upon treatments with PBS or CDDP (20 μM)/PEM (2 μM) for 48 h. **(E)** Dose-toxicity curves showing the cell viability of H1299 transfected with miR-NC1, miR-NC2, and miR-6077 upon CDDP/PEM treatments at the indicated concentrations for 48 h. **(F)** The protein levels of CDKN1A and KEAP1 in H1299 transfected with miR-NC1, miR-NC2, and miR-6077 upon treatments with PBS or CDDP (20 μM)/PEM

(2 μM) for 48 h. Data are presented as mean ± s.d., n = 3 independent repeats. Unpaired, two-tailed t-test; *p < 0.05, **p < 0.01, ***p < 0.001, ****p < 0.0001, ns, not significant.

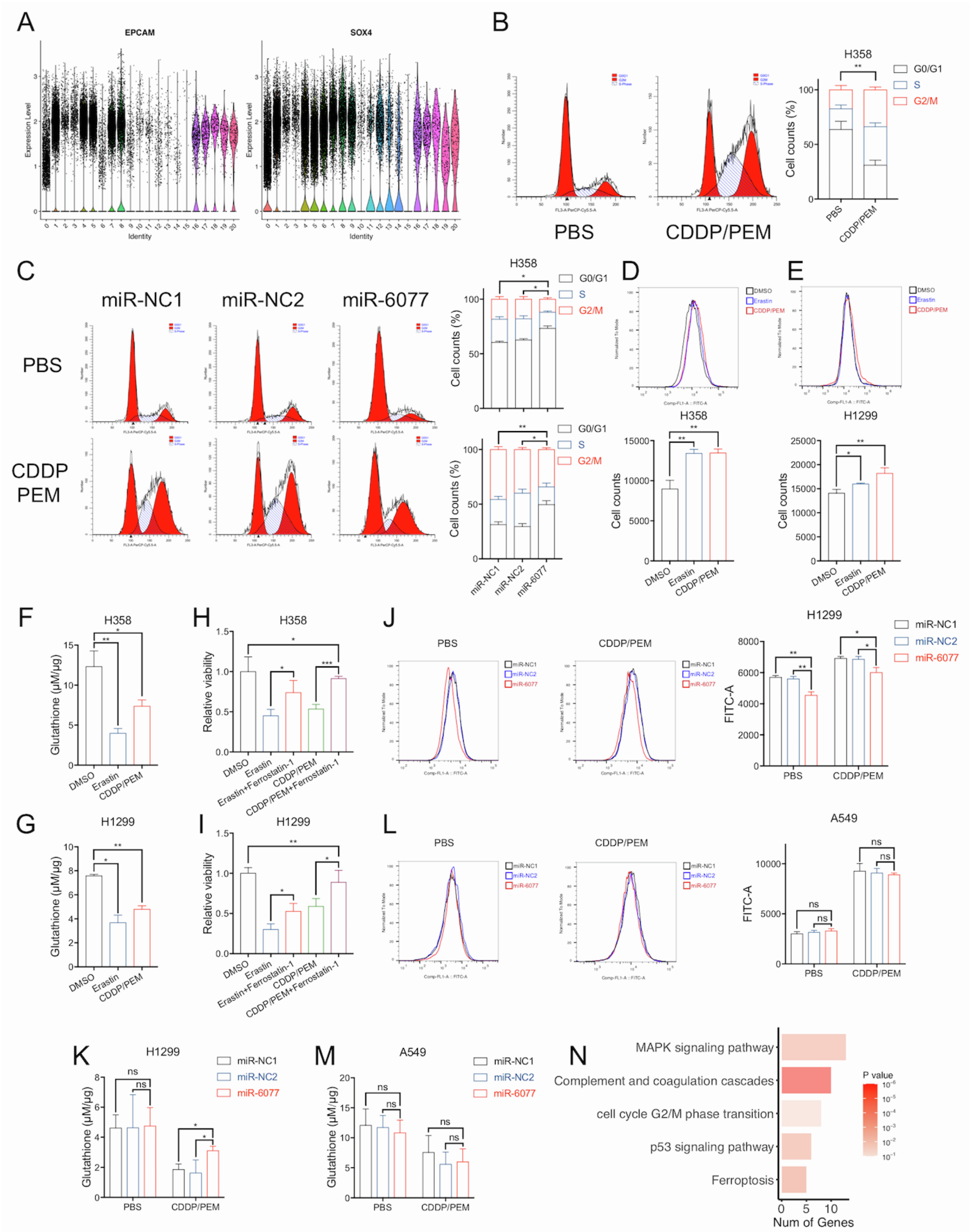

Supplementary Figure 3. miR-6077 protects LUAD cells from cell cycle arrest and ferroptosis induced by CDDP/PEM treatment in different LUAD cell lines. **(A)** Violin plots displaying the expression of well-known representative markers of tumor cells in LUAD patients' surgical sample. The y-axis shows the normalized read count. **(B)** Cell cycle analyses of H358 cells treated with PBS or CDDP (20 μM)/PEM (2 μM) for 48h.

(C) Cell cycle analyses of H358 transfected with miR-NC1, miR-NC2, and miR-6077 upon treatments with PBS or CDDP (20 μM)/PEM (2 μM) for 48 h. **(D, E)** Lipid peroxidation in H358 **(D)** and H1299 **(E)** cells treated with PBS or CDDP (20 μM)/PEM (2 μM) for 48 h. **(F, G)** Relative GLUTATHIONE levels in H358 **(F)** and H1299 **(G)** cells treated with PBS or CDDP (20 μM)/PEM (2 μM) for 48h. **(H, I)** Viability of H358 **(H)** and H1299 **(I)** cells treated as indicated for 48 h. **(J, L)** Lipid peroxidation in H1299 **(J)** and A549 **(L)** transfected with miR-NC1, miR-NC2, and miR-6077 upon treatments with PBS or CDDP (20 μM)/PEM (2 μM) for 48h. **(K, M)** Relative GLUTATHIONE levels in H1299 **(K)** and A549 **(M)** transfected with miR-NC1, miR-NC2, and miR-6077 upon treatments with PBS or CDDP (20 μM)/PEM (2 μM) for 48 h. **(N)** Functional enrichment analysis of the differentially expressed genes between A549 cells transfected with miR-NC and miR-6077 upon CDDP/PEM treatment for 48 h. Data are presented as mean ± s.d., n = 3 independent repeats. Unpaired, two-tailed t-test; *p < 0.05, **p < 0.01, ***p < 0.001, ****p < 0.0001, ns, not significant.

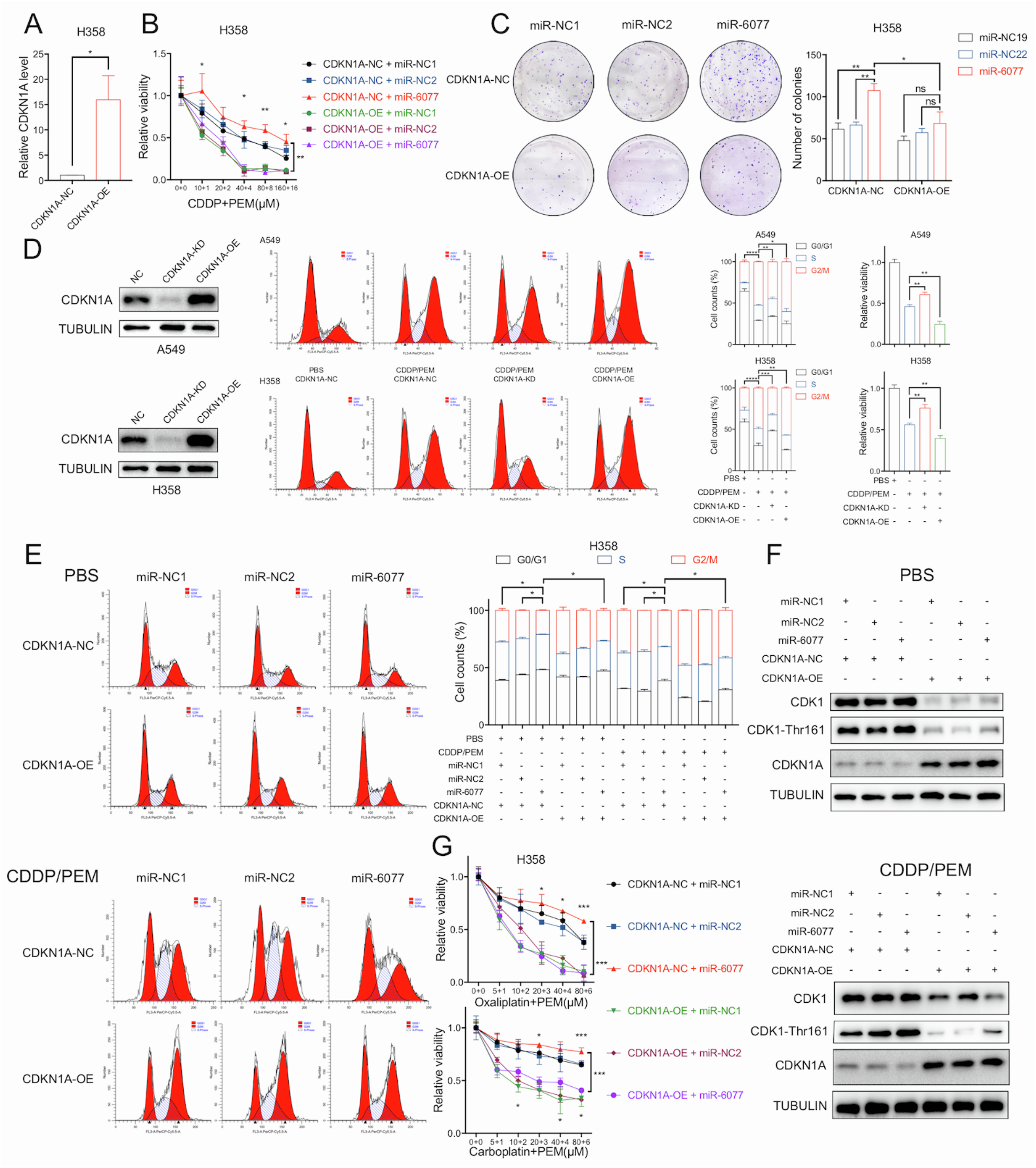

Supplementary Figure 4. CDKN1A overexpression restores cell cycle arrest in different LUAD cells. **(A)** qRT-PCR assay confirming the ectopic overexpression of CDKN1A in H358 cells. **(B)** Dose-toxicity curves showing the viability of H358 cells (with or without CDKN1A overexpression) transfected with miR-NC1, miR-NC2, and miR-6077 upon CDDP/PEM treatments at the indicated concentrations for 48 h. **(C, E, F)** Colony formation ability **(C)**, cell cycle proportion **(E)**, and the protein levels of CDKN1A and its downstream cell cycle regulators **(F)** in H358 cells (with or without

CDKN1A overexpression) transfected with miR-NC1, miR-NC2, and miR-6077 upon treatments with PBS or CDDP (20 μM)/PEM (2 μM) for 14 days **(C)** and 48 h **(E, F)** respectively. **(D)** The impact of CDKN1A-KD and OE on cell cycle proportion and cell's sensitivity to CDDP/PEM in A549 (10 μM CDDP /1 μM PEM) and H358 (20 μM CDDP /2 μM PEM). **(G)** Dose-toxicity curves showing the viability of H358 cells (with or without CDKN1A overexpression) transfected with miR-NC1, miR-NC2, and miR-6077 upon treatments with PEM and oxaliplatin or carboplatin treatments at the indicated concentrations for 48 h. Data are presented as mean ± s.d., n = 3 independent repeats. Unpaired, two-tailed t-test; *$p < 0.05$, **$p < 0.01$, ***$p < 0.001$, ****$p < 0.0001$, ns, not significant.

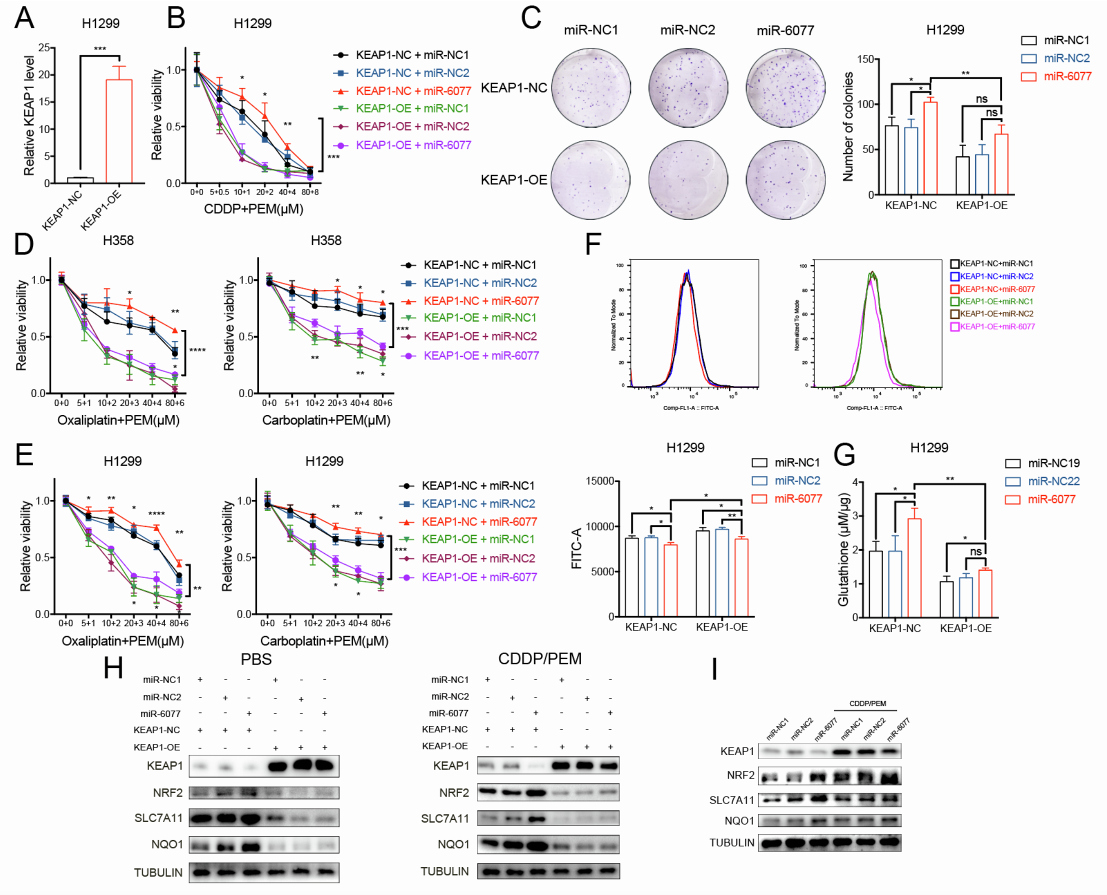

Supplementary Figure 5. KEAP1 overexpression restores ferroptosis in different LUAD cells. **(A)** qRT-PCR assay confirming the ectopic overexpression of KEAP1 in H1299 cells. **(B)** Dose-toxicity curves showing the viability of H1299 cells (with or without KEAP1 overexpression) transfected with miR-NC1, miR-NC2, and miR-6077 upon CDDP/PEM treatments at the indicated concentrations for 48 h. **(C, F, G, H)** Colony formation ability **(C)**, lipid peroxidation **(F)**, relative glutathione levels **(G)**, and the protein levels of KEAP1 and its downstream ferroptosis regulators **(H)** in H1299 cells (with or without KEAP1 overexpression) transfected with miR-NC1, miR-NC2, and miR-6077 upon treatments with PBS or CDDP (20 μM)/PEM (2 μM) for 14 days. **(C)** and 48 h **(F, G, H)** respectively. **(D, E)** Dose-toxicity curves showing the viability of H358 **(D)** and H1299 **(E)** cells (with or without KEAP1 overexpression)

transfected with miR-NC1, miR-NC2, and miR-6077 upon treatments with PEM and oxaliplatin or carboplatin at the indicated concentrations for 48 h. **(I)** Levels of ferroptosis-related proteins in A549-HDR cells with or without miR-6077 overexpression upon treatments with PBS or CDDP (10 μM)/PEM (1 μM) for 48h. Data are presented as mean ± s.d., n = 3 independent repeats. Unpaired, two-tailed t-test; *p < 0.05, **p < 0.01, ***p < 0.001, ****p < 0.0001, ns, not significant.

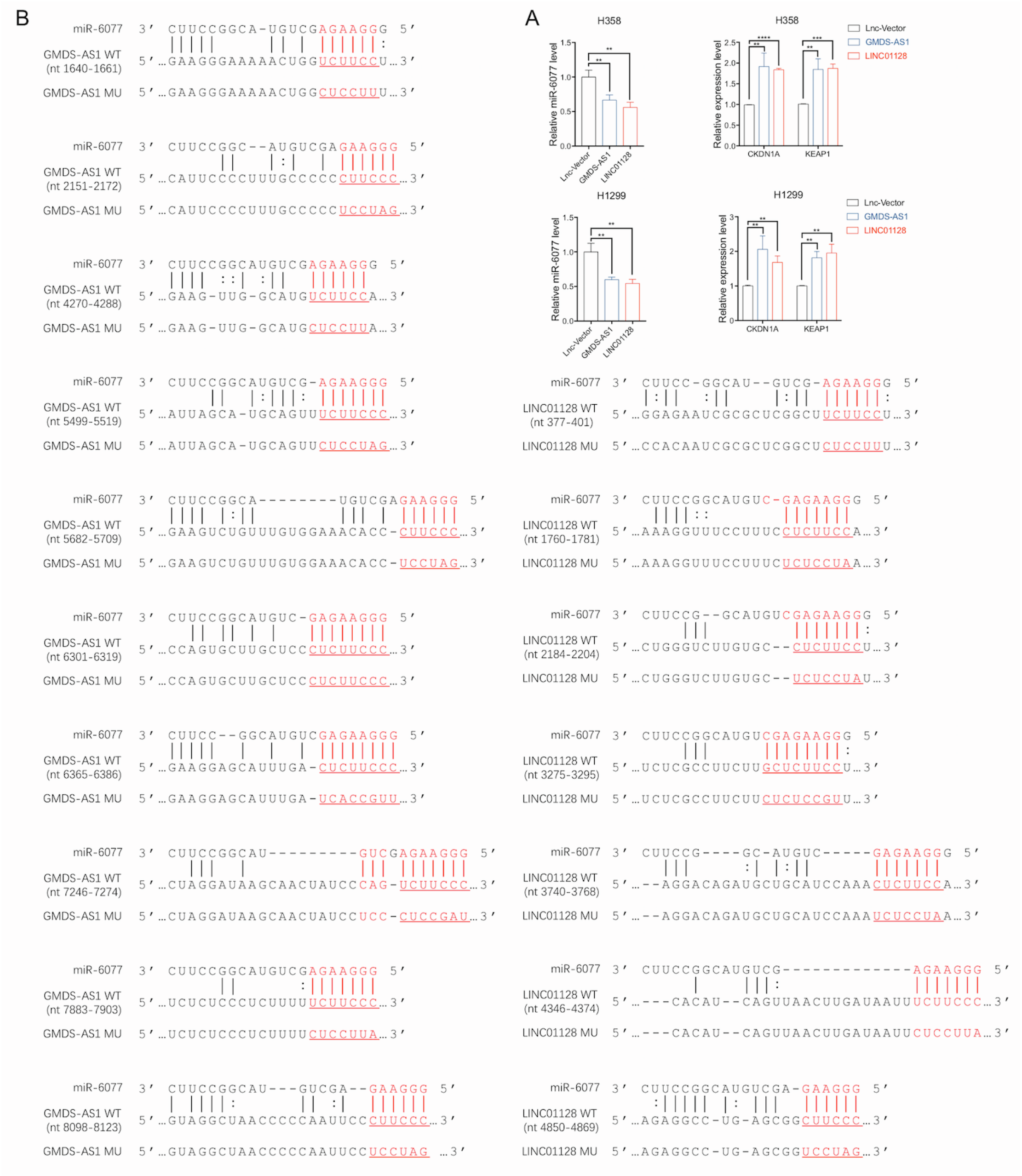

Supplementary Figure 6. Targeting relationship between miR-6077 and GMDS-AS1 /LINC01128. **(A)** Relative expression levels of miR-6077, CDKN1A, and KEAP1 in H358 and H1299 with or without GMDS-AS1/LINC01128 overexpression. **(B)** The predicted target sites of miR-6077 in GMDS-AS1 and LINC01128. Normal and mutant seed regions are highlighted and underlined.

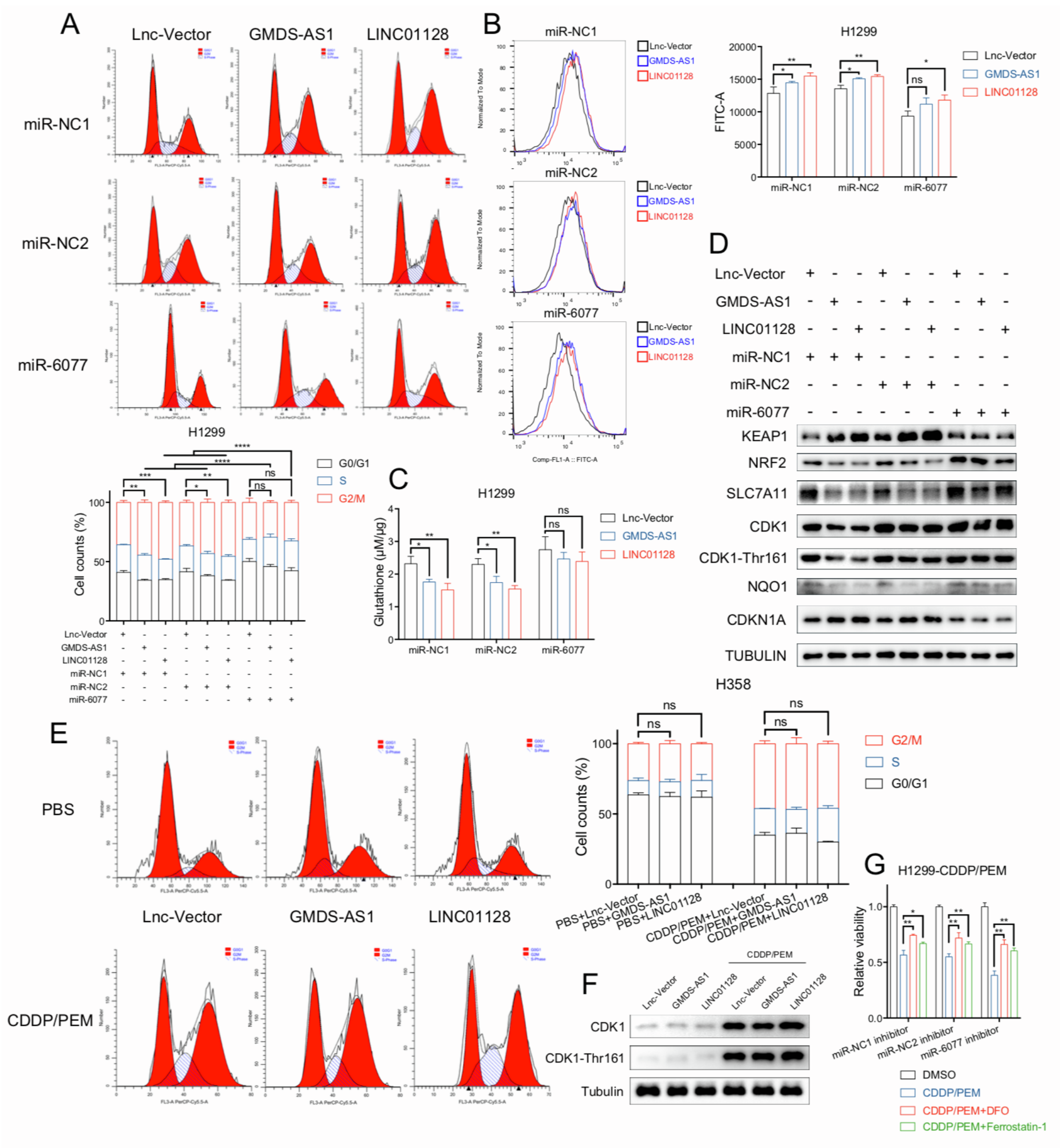

Supplementary Figure 7. Overexpression of GMDS-AS1 and LINC01128 augment the magnitude of CDDP/PEM caused G2/M arrest and lipid peroxidation by inhibiting miR-6077 in different LUAD cells. **(A, B, C, D)** Cell cycle proportion **(A)**, lipid peroxidation levels **(B)**, glutathione levels **(C)**, and the protein levels of CDKN1A/KEAP1 and their downstream molecules **(D)** in GMDS-AS1/LINC01128 overexpressed H1299 cells transfected with miR-NC1, miR-NC2, and miR-6077 upon treatments with PBS or CDDP (20 μM)/PEM (2 μM) for 48 h. **(E, F)** Cell cycle proportion **(E)** and the protein levels of CDK1 **(F)** in GMDS-AS1/LINC01128

overexpressed H358 cells transfected with miR-6077 inhibitor upon treatments with PBS or CDDP (20 μM)/PEM (2 μM) for 48 h. **(G)** Viability of H1299-CDDP/PEM-resistant cells transfected with miR-NC1 inhibitor, miR-NC2 inhibitor, and miR-6077 inhibitor upon treatments as indicated for 48h.
