# Supplementary material for: miR-6077 promotes cisplatin/pemetrexed resistance in lung adenocarcinoma via CDKN1A/cell cycle arrest and KEAP1/ferroptosis pathways
Source: Mol Ther Nucleic Acids. 2022 Mar 28;28:366–86. doi: 10.1016/j.omtn.2022.03.020 (PMC9035384; doi:10.1016/j.omtn.2022.03.020)
Supplement: Document S2. Article plus supplemental information [file mmc2.pdf]

Molecular Therapy
Nucleic Acids
Original Article

miR-6077 promotes cisplatin/pemetrexed resistance in lung adenocarcinoma via CDKN1A/cell cycle arrest and KEAP1/ferroptosis pathways

Guoshu Bi,[1,2,3] Jiaqi Liang,[1,2,3] Mengnan Zhao,[1,2,3] Huan Zhang,[1,2] Xing Jin,[1,2] Tao Lu,[1,2] Yuansheng Zheng,[1,2] Yunyi Bian,[1,2] Zhencong Chen,[1,2] Yiwei Huang,[1,2] Valeria Besskaya,[1,2] Cheng Zhan,[1,2] Qun Wang,[1,2] and Lijie Tan[1,2]

[1]Department of Thoracic Surgery, Zhongshan Hospital, Fudan University, No. 180 Fenglin Rd, Xuhui District, Shanghai 200032, China; [2]Cancer Center, Zhongshan Hospital, Fudan University, Shanghai 200032, China

Lung adenocarcinoma (LUAD) is one of the most common malignancies worldwide. Combination chemotherapy with cisplatin (CDDP) plus pemetrexed (PEM) remains the predominant therapeutic regimen; however, chemoresistance greatly limits its curative potential. Here, through CRISPR-Cas9 screening, we identified miR-6077 as a key driver of CDDP/PEM resistance in LUAD. Functional experiments verified that ectopic overexpression of miR-6077 desensitized LUAD cells to CDDP/PEM in both cell lines and patient-derived xenograft models. Through RNA sequencing in cells and single-cell sequencing of samples from patients with CDDP/PEM treatments, we observed CDDP/PEM-induced upregulation of CDKN1A and KEAP1, which in turn activated cell-cycle arrest and ferroptosis, respectively, thus leading to cell death. Through miRNA pull-down, we identified and validated that miR-6077 targets CDKN1A and KEAP1. Furthermore, we demonstrated that miR-6077 protects LUAD cells from cell death induced by CDDP/PEM via CDKN1A-CDK1-mediated cell-cycle arrest and KEAP1-NRF2-SLC7A11/NQO1-mediated ferroptosis, thus resulting in chemoresistance in multiple LUAD cells both *in vitro* and *in vivo*. Moreover, we found that GMDS-AS1 and LINC01128 sensitized LUAD cells to CDDP/PEM by sponging miR-6077. Collectively, these results imply the critical role of miR-6077 in LUAD's sensitivity to CDDP/PEM, thus providing a novel therapeutic strategy for overcoming chemoresistance in clinical practice.

INTRODUCTION

Lung cancer is one of the most commonly diagnosed cancers (11.4% of total cases), and it remains the leading cause of cancer deaths (18.0% of total cancer deaths),[1] of which lung adenocarcinoma (LUAD) constitutes the majority.[2,3] Currently, surgery combined with perioperative chemotherapy or radiotherapy remains the standard treatment for lung malignancies. As recommended by National Comprehensive Cancer Network (NCCN) guidelines (v3.2021), combination chemotherapy with platinum-based compounds, such as cisplatin (CDDP) plus pemetrexed (PEM), has been the most actively used front-line therapeutic regimen for LUAD for decades. However, the anti-tumor efficiency of CDDP/PEM is usually limited by intrinsic or acquired multidrug resistance (MDR) in patients with LUAD, thus leading to failure in treatment and poorer prognosis.[4] Despite extensive efforts in this area related to tumor cells' ability to regulate drug efflux, DNA damage repair, the cell cycle and apoptosis, and signaling pathways affecting cell fate,[5–7] the crucial factors and mechanisms potentially driving CDDP/PEM MDR remain elusive.

CDDP exerts anti-cancer effects mainly by interacting with DNA to form mostly intrastrand cross-link adducts, which activates a series of signaling pathways and cell death.[8] Although numerous clinical trials have verified the superiority of PEM to other chemotherapeutic agents,[9–11] few studies have explored the mechanisms driving PEM resistance. PEM's cytotoxicity is mediated by inhibition of the activity of the folate-dependent enzyme thymidylate synthase, which is required for *de novo* syntheses of nucleotides, thus resulting in ineffective DNA synthesis and repair, followed by cell-cycle arrest.[7,12] Meanwhile, both CDDP and PEM have been proposed to trigger excessive production of intracellular reactive oxygen species (ROS) by activating the enzyme NADPH oxidase, which catalyzes on-electron reduction of O_2 and generates superoxide, thus damaging nucleic acids, proteins, and lipids.[13–15] Beyond cell apoptosis, recent studies have demonstrated that these ROS-related effects might induce forms of cell death that are independent of DNA damage, such as ferroptosis.[16] Therefore, aberrant alterations in these biological processes may render tumor cells insensitive to CDDP/PEM, and identifying the key molecules involved in these pathways is critical to reverse the cells' chemoresistance.

Received 15 November 2021; accepted 27 March 2022;
https://doi.org/10.1016/j.omtn.2022.03.020.

[3]These authors contributed equally

Correspondence: Cheng Zhan, Department of Thoracic Surgery, Zhongshan Hospital, Fudan University, No. 180 Fenglin Rd, Xuhui District, Shanghai 200032, China.
E-mail: czhan10@fudan.edu.cn
Correspondence: Qun Wang, Department of Thoracic Surgery, Zhongshan Hospital, Fudan University, No. 180 Fenglin Rd, Xuhui District, Shanghai 200032, China.
E-mail: wang.qun@zs-hospital.sh.cn

 © 2022 The Author(s).
This is an open access article under the CC BY license (http://creativecommons.org/licenses/by/4.0/).

A currently expanding field of research is the study of microRNA (miRNA), a class of endogenously expressed, ∼22 nucleotide noncoding RNAs that post-transcriptionally silence gene expression by binding the 3′ untranslated regions (3′ UTRs). miRNAs play indispensable roles in numerous biological processes, including tumorigenesis, apoptosis, and ferroptosis.[17,18] However, knowledge of miRNA involvement in the regulation of tumor cell chemoresistance remains scarce.

The potential function of miR-6077, a newly characterized 21 bp miRNA, in tumorigenesis and progression, and its involvement in LUAD sensitivity to chemotherapy or radiotherapy, has rarely been reported. In this study, through a series of high-throughput screening approaches, including clustered regularly interspaced short palindrome repeats (CRISPR)-Cas9 screening, miRNA pull-down, and RNA sequencing (RNA-seq), we discovered that miRNA-6077 confers CDDP/PEM resistance on LUAD cells by directly targeting CDKN1A and Kelch-like ECH-associated protein 1 (KEAP1), thus protecting the cells from CDDP/PEM-induced cell-cycle arrest and ferroptosis, respectively.

RESULTS

Genome-wide CRISPR-Cas9 screening system reveals miR-6077 as a key regulator of CDDP/PEM sensitivity in LUAD

To gain insight into the genetic modifiers that potentially regulate LUAD cell sensitivity to combination chemotherapy with CDDP plus PEM, we applied a genome-scale CRISPR-Cas9 loss-of function screening assay with a pooled lentiviral single-guide RNA (sgRNA) library targeting 20,060 genes and 1,854 miRNAs. In the primary screen, we transduced the sgRNAs into A549 cells with constitutive Cas9 expression, during which time, by precisely controlling the volume of lentivirus, we ensured that only one or no sgRNA was transduced into each cell, and the cells without any sgRNAs were eliminated by puromycin. Therefore, in each cell, only one gene or miRNA was randomly knocked out. We treated these cells with PBS or CDDP/PEM, thus ensuring that the genes or miRNAs whose deletion significantly sensitized or conferred resistance to CDDP/PEM could be selected from the primary screen. After treatment for 7 or 14 days, the cells were subjected to next-generation RNA-seq to measure the relative abundance of cell populations with different genes or miRNAs knocked out (Figure 1A), in which differential sgRNA expression was evaluated in the form of ΔLogFC between the CDDP/PEM- and the PBS-treated groups. From this primary screen, 11 miRNAs were selected for further exploration because their inactivation caused significant sensitization of A549 cells to CDDP/PEM (Figure 1B, defined as $|logFC| > 2.7$ and $|logFC_{(day\ 14)}| > |logFC_{(day\ 7)}|$). Next, we chose the five candidate miRNAs obtained above that had single spliceosomes and had not been previously reported (Figure 1C). As shown in Figure 1D, miR-6077 emerged as the lead hit from the secondary screen, because its overexpression led to a significant increase in cell viability only in the presence of CDDP/PEM, thus suggesting that miR-6077 might serve as a key regulator of LUAD resistance to CDDP/PEM chemotherapy.

To further verify the screening results, we overexpressed miR-6077 in several LUAD cell lines and examined the cell viability after 48 h exposure to single or combined use of CDDP and PEM with gradient doses. In agreement with the CRISPR-Cas9 screening, the results revealed that miR-6077 significantly desensitized both A549 and H358 cell lines to CDDP/PEM treatment (Figures 1E and S1A) but did not affect their proliferation rate (Figures S1B–S1C). This finding was further validated by high-content analysis, through which we monitored the dynamic change in GFP-overexpressing A549 cells transfected with miR-NCs and miR-6077 in the presence of PBS or CDDP/PEM (Figure S1D).

Similar results were observed when the miR-6077-overexpressing cells were treated with PEM and other platinum-based components, including carboplatin and oxaliplatin (Figures 1F and S1E). In addition, upregulation of miR-6077 significantly enhanced the colony formation potential of LUAD cell lines only in the presence of CDDP/PEM, while miR-7702 and miR-6855-5p, which were identified to contribute to cells' resistance to CDDP/PEM, as well, in the primary and secondary screenings, also exhibited a similar effect in the presence of CDDP/PEM, but not as significantly as miR-6077 (Figures 1G and S1F).

Together, these results corroborated our primary CRISPR-Cas9 screen findings, providing evidence that miR-6077 confers CDDP/PEM resistance to LUAD and may serve as a potential target in LUAD treated with a combination chemotherapy of CDDP plus PEM, as well as other types of platinum.

miR-6077 is associated with CDDP/PEM resistance in patients with LUAD

To further define the clinical relevance of miR-6077, we investigated its expression in tumor samples originally derived from 20 patients with LUAD (cohort A) who had received neoadjuvant CDDP/PEM therapy before tumor resection. According to Response Evaluation Criteria in Solid Tumors (RECIST) edition 1.1 criteria, the patients with LUAD were divided into CDDP/PEM-resistant ($n = 10$) or -sensitive ($n = 10$) groups. Considering the intratumoral heterogeneity, we isolated the tumor cells from bulk tumor tissue through flow cytometry (Figure S1G). As shown in Figure 1H, the miR-6077 levels were significantly higher in tumors from the CDDP/PEM-resistant group than in the sensitive tissues (0.717 ± 0.317 in CDDP/PEM-sensitive group and 1.224 ± 0.258 in the resistant group; fold change = 1.706, $p = 0.002$), whereas in lymphocytes or other non-tumoral cells the levels of miR-6077 were much lower than those in tumor cells (Figure S1H), suggesting the possibility of specifically targeting miR-6077 in tumor cells rather than other cell types. Moreover, when measuring the predictive value of miR-6077 in the assessment of tumor response to CDDP/PEM therapy, we found that the area under the receiver operating characteristic (ROC) curve of the miR-6077 level was 0.850 (Figure 1I). Collectively, these data illustrated that miR-6077 is correlated with CDDP/PEM resistance in patients with LUAD.

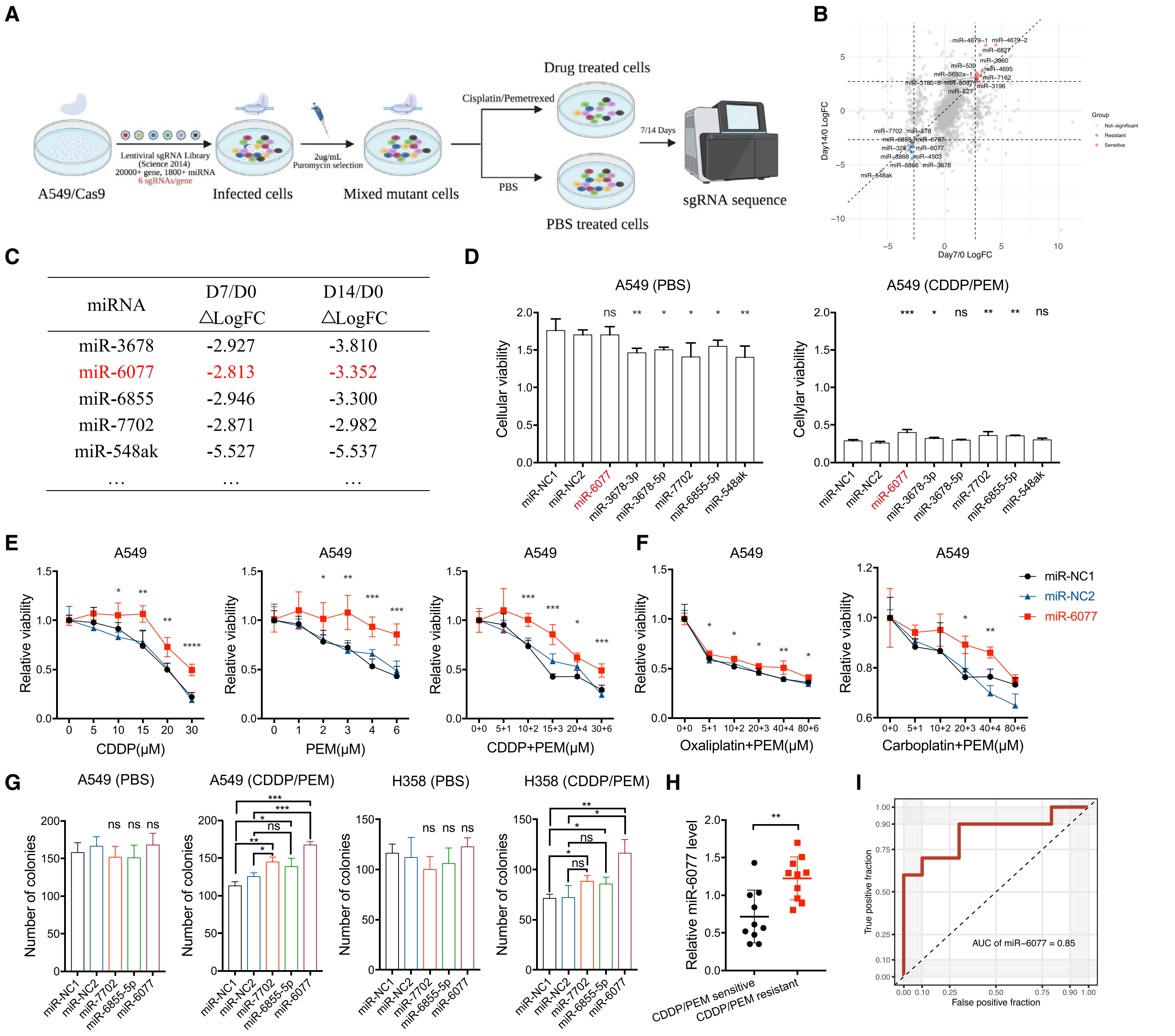

Figure 1. Genome-wide CRISPR-Cas9 screening identifies miR-6077 as a determinant of CDDP/PEM sensitivity in LUAD

(A) Schematic outline of the CRISPR-Cas9 screening workflow in the A549 cell line. (B) Scatterplot showing the top hits in 7- (horizontal axis) or 14-day (vertical axis) CDDP/PEM-treated A549 cells, highlighting miR-6077. Red dots represent miRNAs whose depletion led to chemoresistance, while blue dots represent sensitivity. For presentation purpose, only miRNAs meeting the criteria of $|\log FC| > 2.7$ and $|\log FC_{(day\ 14)}| > |\log FC_{(day\ 7)}|$ are highlighted. (C) CRISPR-Cas9 results of top five miRNAs potentially conferring resistance to CDDP/PEM. (D) Cell viability in miRNA-transfected A549 cells treated with PBS (left) or CDDP (10 μM)/PEM (1 μM) (right) for 48 h. (E) Dose-toxicity curves showing the viability of A549 cells transfected with miR-NC1, miR-NC2, and miR-6077 upon CDDP or PEM treatment at the indicated concentrations for 48 h. (F) Viability of A549 cells transfected with miR-NC1, miR-NC2, and miR-6077 upon treatment with PEM and oxaliplatin or carboplatin at the indicated concentrations for 48 h. (G) Quantification of the colony formation ability of A549 and H358 cells transfected with miR-NC1, miR-NC2, and miR-6077 upon treatment with PBS or CDDP for 14 days. (H) Relative expression levels of miR-6077 in CDDP/PEM-sensitive or -resistant tumor tissues obtained from LUAD patients. (I) ROC curve of miR-6077 exhibiting its predicting value when assessing LUAD patients' response to CDDP/PEM therapy. Data are presented as the mean ± SD, n = 3 independent repeats. Unpaired, two-tailed t test; *$p < 0.05$, **$p < 0.01$, ***$p < 0.001$, ****$p < 0.0001$; ns, not significant.

CDKN1A and KEAP1 are direct targets of miR-6077

To gain further insight into miR-6077's mechanism of action in the regulation of LUAD chemoresistance, we used a pull-down assay followed by RNA-seq to search for the candidate genes whose 3′ UTR binds miR-6077 in the A549 cell line. We also predicted the potential targets of miR-6077 on the basis of the publicly available bioinformatic database miRWalk (Figure 2A).[19] The influence of inactivation of candidate genes by CRISPR-Cas9 on cell resistance to

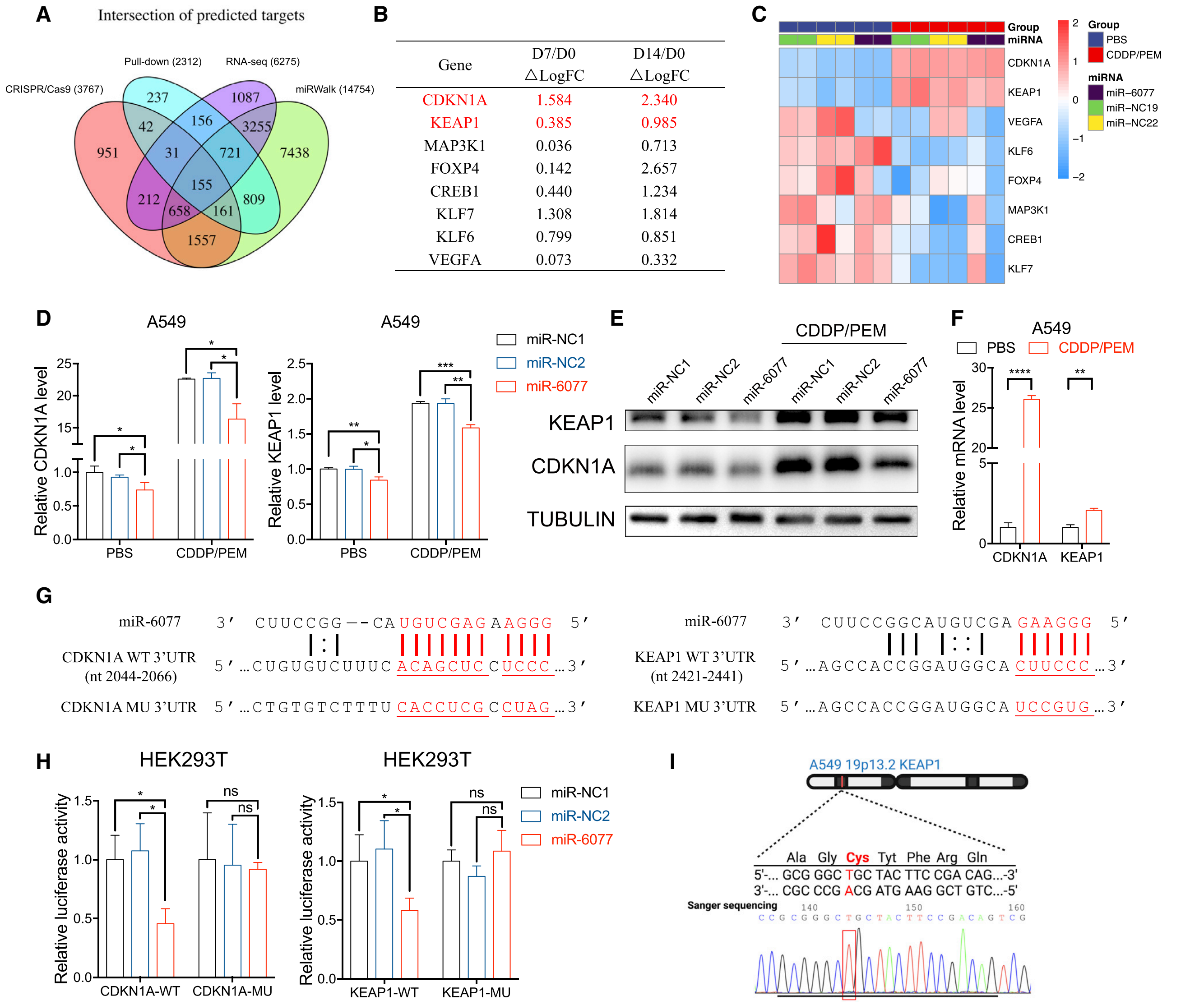

Figure 2. CDKN1A and KEAP1 are direct targets of miR-6077

(A) Venn plot showing the intersection of predicted targets of miR-6077. (B) The CRISPR-Cas 9 results of top eight predicted targets of miR-6077 whose depletion potentially confers resistance to CDDP/PEM. (C) Heatmap showing the relative expression levels of the eight predicted targets of miR-6077 in A549 cells transfected with miR-NC1, miR-NC2, and miR-6077 upon treatment with PBS or CDDP (10 μM)/PEM (1 μM) for 48 h. The results were obtained from RNA-seq. (D and E) Quantitative real-time PCR and western blotting assays showing the mRNA (D) and protein (E) levels of CDKN1A and KEAP1 in A549 cells transfected with miR-NC1, miR-NC2, and miR-6077 upon treatment with PBS or CDDP (10 μM)/PEM (1 μM) for 48 h. (F) Quantitative real-time PCR showing the relative expression levels of CDKN1A and KEAP1 in A549 cells upon treatment with PBS or CDDP (10 μM)/PEM (1 μM) for 48 h. (G) The predicted target sites of miR-6077 in the 3′ UTR of CDKN1A and KEAP1. Normal and mutant seed regions are highlighted and underlined. (H) Luciferase reporter plasmids containing wild- or mutant-type CDKN1A (left) and KEAP1 (right) were co-transfected into HEK293T cells with miR-NC1, miR-NC2, or miR-6077. Bar plots exhibit the luciferase activity of the transfected cells. (I) Sanger sequencing confirming the G333C inactivating mutation within the first Kelch domain (KLD) of KEAP1 in A549 cells. Data are presented as the mean ± SD, n = 3 independent repeats. Unpaired, two-tailed t test; *p < 0.05, **p < 0.01, ***p < 0.001; ns, not significant.

CDDP/PEM described above was also considered (Figures 1A, 1B, and 2B). Furthermore, next-generation RNA-seq was performed in A549 transfected with miR-6077 or miR-NC mimics in the absence or presence of CDDP/PEM, and the genes dramatically dysregulated after CDDP/PEM treatment were considered to be involved in the regulation of LUAD sensitivity to the combination therapy (Figure 2C). On the basis of this evidence from both structural and functional assays, we screened CDKN1A and KEAP1 for further experimental validation (Figures 2A–2C). As shown in Figures 2D, 2E, S2A, and S2B, the overexpression of miR-6077 significantly impeded

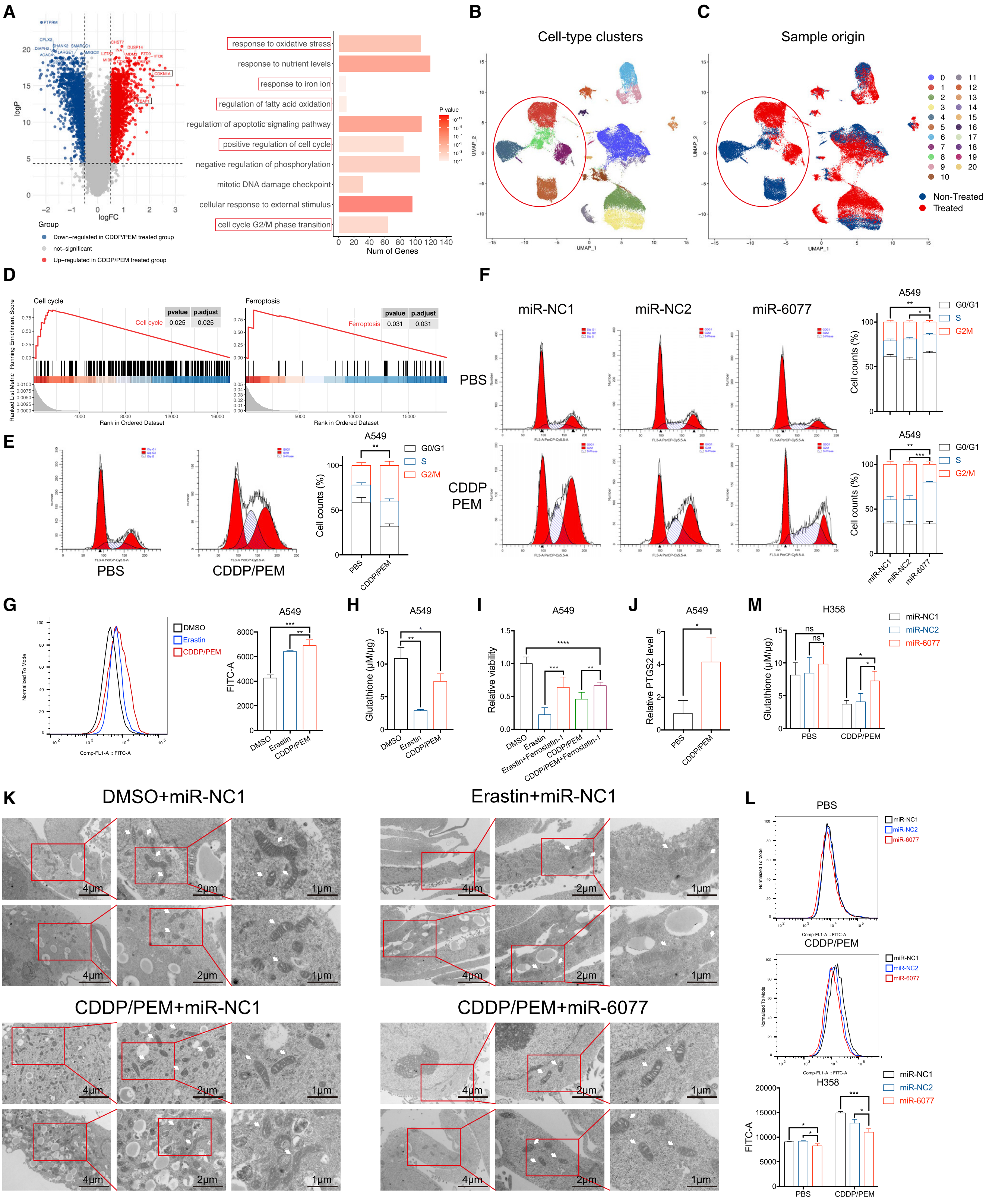

(legend on next page)

the expression of CDKN1A and KEAP1 at both the mRNA and the protein level in LUAD cell lines. Furthermore, the levels of six other potential targets identified through the screening process—MAP3K1, FOXP4, CREB1, KLF7, KLF6, and VEGFA—were also tested by western blotting, but the miR-6077 did not influence their expression (Figure S2C). Moreover, because the CDDP/PEM treatment resulted in a dramatic upregulation of CDKN1A and KEAP1 in LUAD cell lines (Figures 2C, 2E, 2F, S2B, and S2D), we also examined the inhibitory effects of miR-6077 on the two target genes in the presence of CDDP/PEM. Similar results were observed, thus implying that the CDKN1A and KEAP1 enhancement caused by CDDP/PEM treatment was partly abrogated by miR-6077 (Figures 2D, 2E, S2A, and S2B).

To further investigate whether CDKN1A and KEAP1 are truly direct targets of miR-6077, we conducted dual-luciferase reporter assays by constructing plasmids with a firefly and *Renilla* reporter containing either the wild-type or the mutant 3′ UTR of CDKN1A and KEAP1 (Figure 2G). Co-transfection of the plasmids and miR-6077 in 293T cells significantly attenuated the luciferase activity of the reporter vectors containing the wild-type 3′ UTR of both CDKN1A and KEAP1, whereas the mutation in the seed sequence of the predicted miR-6077 binding position blocked this suppressive effect (Figure 2H). Collectively, these findings indicated that CDKN1A and KEAP1 are direct downstream targets of miR-6077.

Notably, the A549 cell line is characterized by a G333C inactivating mutation within the first Kelch domain (KLD) of KEAP1, thus preventing us from exploring the actual effects of miR-6077 on cell resistance to CDDP/PEM via targeting of KEAP1. We validated the existence of this mutation by Sanger sequencing (Figure 2I). Therefore, in the subsequent analyses, all the KEAP1-related assays were also performed in the H1299 cell line, which has wild-type KEAP1. We thus validated the association between miR-6077 and CDDP/PEM resistance as well as its suppression of CDKN1A and KEAP1 in H1299 (Figures S2E and S2F). The two target genes were all expressed in the cell lines used in this study (Figure S2G).

miR-6077 reverses cell-cycle arrest and ferroptosis induced by CDDP/PEM

We further assessed the downstream cellular signaling pathways through which miR-6077 confers CDDP/PEM resistance via CDKN1A and KEAP1 inhibition. On the basis of the differentially expressed gene (DEG) identification between A549 cells treated with CDDP/PEM or left untreated, and subsequent functional enrichment analysis, we observed that in addition to the CDKN1A and KEAP1 activation (Figure 2C), a series of genes involved in cell-cycle- and ferroptosis-related biological pathways, including cell-cycle G2/M phase transition, regulation of fatty acid oxidation, and response to oxidative stress, were significantly dysregulated after CDDP/PEM treatment (Figure 3A).

We also validated this finding in tumor specimens derived from eight patients with LUAD who had or had not received preoperative CDDP/PEM treatment. To eliminate the intratumoral heterogeneity derived from other non-cancerous cells located in the tumor microenvironment,[20,21] we used single-cell (sc) RNA-seq to precisely depict the transcriptive alterations induced by CDDP/PEM therapy. After standardized quality control, 38,599 and 32,372 cells from treated and untreated patients with LUAD, respectively, were included in subsequent analysis. On the basis of the CellMarker dataset and our previous studies, tumor cells (marked by EPCAM and SOX4) were isolated from other cell populations through dimensionality reduction and unsupervised clustering analysis (Figures 3B and S3A). Having confirmed the sample type of origin (treated or untreated) of the cell populations, we then performed gene set enrichment analysis (GSEA) between groups and again observed significant enrichment of cell-cycle- and ferroptosis-related pathways (Figures 3C and 3D). These results from both traditional RNA-seq and scRNA-seq indicated that the two pathways were activated by CDDP/PEM treatment in LUAD cells.

On one hand, numerous studies support that the induction of CDKN1A by p53 after DNA damage caused by radiotherapy or chemotherapy, such as platinum and PEM, inhibits both the expression and the function of cyclin-dependent kinases (CDKs), thus resulting in cell-cycle arrest and subsequent proliferation inhibition or even cell death, whereas the genetic alterations enabling tumor cells to overcome the cell-cycle arrest desensitize the cells to cytotoxic drugs.[22–25] In agreement with findings from previous studies, we observed dramatically aberrant cell-cycle progression characterized by G2/M arrest in A549 and H358 after CDDP/PEM exposure (Figures 3E and S3B). However, this effect was rescued by ectopic expression of miR-6077 (Figures 3F and S3C); these results implied that miR-6077 protects CDDP/PEM-treated LUAD cells against

Figure 3. miR-6077 protects LUAD cells from cell-cycle arrest and ferroptosis induced by CDDP/PEM treatment

(A) Volcano plot (left) and bar plot (right) showing the differentially expressed genes in A549 cells treated with PBS or CDDP (10 μM)/PEM (1 μM) for 48 h and corresponding Gene Ontology (GO) functional enrichment analyses, respectively. (B) The uniform manifold approximation and projection (UMAP) plots visualizing the cell-type clusters of the 70,971 high-quality cells based on the expression of known marker genes. (C) The sample origin of the 70,971 cells. (D) Gene set enrichment analysis showing the activation of cell-cycle- and ferroptosis-related signaling pathways when analyzing the differentially expressed genes between the cell populations derived from patients receiving or not receiving neoadjuvant CDDP/PEM treatment. (E) Cell-cycle analyses of A549 cells treated with PBS or CDDP (10 μM)/PEM (1 μM) for 48 h. (F) Cell-cycle analyses of A549 cells transfected with miR-NC1, miR-NC2, and miR-6077 upon treatment with PBS or CDDP (10 μM)/PEM (1 μM) for 48 h. (G and H) Lipid peroxidation (G) and relative glutathione levels (H) in A549 cells treated with PBS or CDDP (10 μM)/PEM (1 μM) for 48 h. (I) Viability of A549 cells treated as indicated for 48 h. (J) Relative expression levels of PTGS2 in A549 cells treated with PBS or CDDP (10 μM)/PEM (1 μM) for 48 h. (K) Transmission electron microscopy images of H1299 cells subjected to indicated treatments (white arrowheads indicate mitochondria). (L and M) Lipid peroxidation (L) and relative glutathione levels (M) in H358 transfected with miR-NC1, miR-NC2, and miR-6077 upon treatment with PBS or CDDP (20 μM)/PEM (2 μM) for 48 h. Data are presented as the mean ± SD, n = 3 independent repeats. Unpaired, two-tailed t test; *p < 0.05, **p < 0.01, ***p < 0.001, ****p < 0.0001; ns, not significant.

cell-cycle arrest, thus leading to chemoresistance and cell survival. Interestingly, in the NC groups in which LUAD cells were exposed to PBS rather than CDDP/PEM, miR-6077 overexpression also slightly decreased the G2/M percentage, thereby further emphasizing the role of miR-6077 in the regulation of cell-cycle progression.

On the other hand, given that CDDP/PEM induces cytotoxicity partially through generation of ROS and depletion of GSH, both of which are central molecules in ferroptosis,[26,27] we determined that CDDP and PEM induce ferroptosis in cancer cells. As expected, we observed accumulation of lipid peroxidation and GSH depletion, the hallmarks of ferroptosis, in CDDP/PEM-treated A549 cell lines (Figures 3G and 3H). Furthermore, the inhibiting effects of CDDP/PEM on tumor cell proliferation and viability were partially reversed by ferrostatin-1, a specific ferroptosis inhibitor (Figure 3I). These findings extended to other types of LUAD cell lines, including H358 and H1299 (Figures S3D–S3I). Moreover, ferroptosis induction is accompanied by the increased expression of corresponding marker genes such as PTGS2. Concordantly, CDDP/PEM exposure led to PTGS2 upregulation only in A549 cells, and not in H358 and H1299, in which its expression was naturally too low to be detected (Figure 3J).[28] Finally, transmission electron microscopy revealed that after CDDP/PEM treatment, cells exhibited typical features of ferroptosis (Figure 3K), characterized by shrunken mitochondria with enhanced membrane density.[26] Nevertheless, the above-described biochemical and morphological alterations were significantly attenuated by miR-6077 in H358 and H1299, but not in A549 cells (Figures 3L, 3M, and S3J–S3M). This phenomenon might have been due to the G333C inactivating mutation within the first KLD of KEAP1 in A549 cell lines.

Together, these results revealed that, beyond classic forms of cell death, such as apoptosis and necrosis, ferroptosis also accounts for the cytotoxicity induced by CDDP/PEM, and miR-6077 partially abrogates this effect and desensitizes LUAD cells to CDDP/PEM treatment. In addition to the DEG analysis between CDDP/PEM-treated and untreated cells, we compared the DEGs between miR-6077- and miR-NC-treated cells and noted that G2/M transition and ferroptosis pathways were enriched (Figure S3N). Because CDKN1A and KEAP1 are key molecules in the regulation of cell cycle and ferroptosis, respectively, it is reasonable to hypothesize that these two pathways mediated the CDDP/PEM resistance induced by miR-6077 and its direct targets CDKN1A and KEAP1.

miR-6077 targets CDKN1A, thus overcoming G2/M arrest and conferring CDDP/PEM resistance

To determine the mediating role of CDKN1A in miR-6077-induced G2/M-arrest attenuation and subsequent CDDP/PEM resistance, we stably overexpressed or knocked down CDKN1A in LUAD cell lines (Figures 4A, 4E, S4A, S4D, and S4F). Cytotoxicity and colony formation assays demonstrated that CDKN1A overexpression rescued the CDDP/PEM desensitization effect induced by miR-6077 (Figures 4B, 4C, S4B, and S4C). In the absence of CDDP/PEM, ectopically overexpressed CDKN1A promoted G2/M arrest, as determined by flow cytometry assays. Furthermore, after CDDP/PEM treatment for 48 h, miR-6077 attenuated the G2/M arrest caused by chemotherapy drugs, but this protective effect was dramatically abolished by CDKN1A upregulation (Figures 4D and S4E). Notably, CDKN1A has been proposed to be required for sustained G2/M arrest after activation of the DNA damage checkpoint, not only because it suppresses the expression of CDK1, which plays an essential role in the modulation of G2/M transition, but also because it blocks the activating phosphorylation of CDK1 on Thr161.[23,24,29] Therefore, we examined the levels of CDK1 and p-CDK1-Thr161 by western blotting. As expected, miR-6077's inhibitory effect on CDKN1A resulted in upregulation of both CDK1 and its active form p-CDK1-Thr161, whereas this effect was rescued by CDKN1A restoration (Figure 4F). Similar results were observed in H358 cells (Figure S4F). Furthermore, the effects of CDKN1A restoration also sensitized cells to carboplatin and oxaliplatin when administered in combination with PEM (Figures 4F and S4G). Together, our findings indicated that miR-6077 desensitizes LUAD cells to CDDP/PEM in a CDKN1A-dependent manner, as well as CDKN1A's downstream cell-cycle modulation.

miR-6077 targets KEAP1, thus decreasing ferroptosis and conferring CDDP/PEM resistance

Previous studies have provided adequate evidence that the KEAP1/nuclear factor erythroid 2-related factor (NRF2) antioxidative signaling pathway is a key negative regulator of ferroptosis in tumor cells through the transcriptional activation of genes involved in ROS and iron metabolism, such as SLC7A11 and NQO1,[30–32] thus supporting our hypothesis of KEAP1's potential function in promoting CDDP/PEM-induced ferroptosis and the acquisition of chemoresistance. To validate this possibility, we restored KEAP1 expression in LUAD cell lines (Figures 5A and S5A) and observed that the KEAP1 upregulation abolished miR-6077's influence by inhibiting cell viability and colony formation ability in the presence of CDDP/PEM (Figures 5B, 5C, S5B, and S5C), as well as neutralizing miR-6077's protection against ferroptosis caused by CDDP/PEM. The results from cytotoxicity assays extended to oxaliplatin and carboplatin (Figures S5D and S5E). Both the C11-BODIPY staining assays, which measured the accumulation of lipid peroxidation, and the GSH depletion experiments supported this conclusion (Figures 5D, 5E, S5F, and S5G). Moreover, as exhibited by western blotting, miR-6077 induced the expression of NRF2, a transcriptive factor, and its downstream target genes SLC7A11 and NQO1, both of which contribute to ferroptosis resistance, whereas this effect was markedly abrogated by KEAP1 restoration (Figures 5F and S5H). However, in the PBS group, the change in NRF2 and its downstream factors was not significant after miR-6077 transfection in the presence of ectopically overexpressed KEAP1. This phenomenon might be explained by KEAP1-dependent NRF2 degradation. In such conditions, the cells were not subjected to external stress, so the NRF2 level was maintained at a quite low level. Furthermore, when we ectopically overexpressed KEAP1 in the LUAD cells, the NRF2 level was further suppressed, and the impact of miR-6077 was abrogated by the rescue of KEAP1. Therefore, the "double inhibition" on NRF2 made it difficult

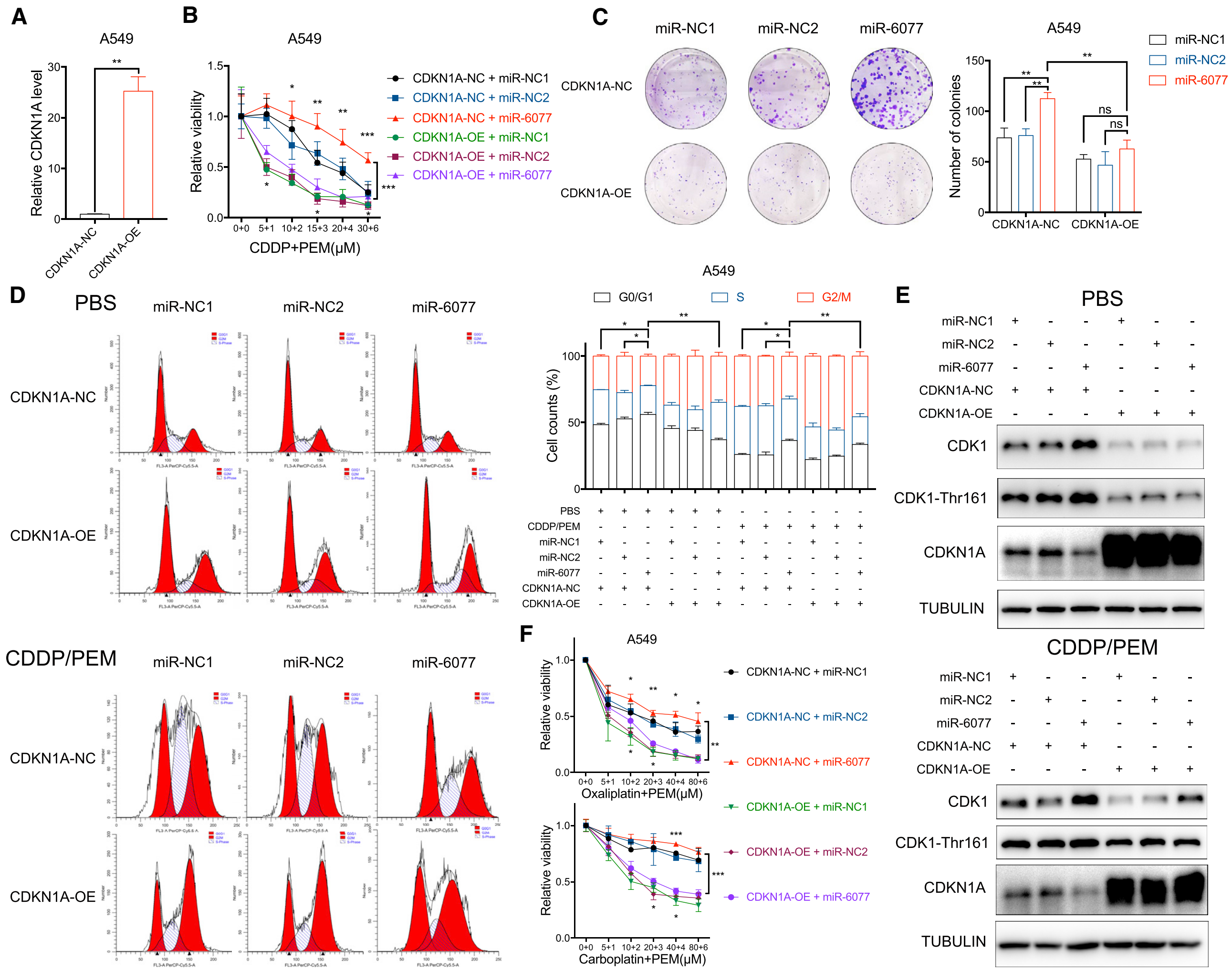

Figure 4. miR-6077 protects LUAD cells from CDDP/PEM-induced cell-cycle arrest by directly targeting CDKN1A

(A) Quantitative real-time PCR assay confirming the ectopic overexpression of CDKN1A in A549 cells. (B) Dose-toxicity curves showing the viability of A549 cells (with or without CDKN1A overexpression) transfected with miR-NC1, miR-NC2, and miR-6077 upon CDDP/PEM treatment at the indicated concentrations for 48 h. (C–E) Colony formation ability (C), cell-cycle proportion (D), and protein levels of CDKN1A and its downstream cell-cycle regulators (E) in A549 cells (with or without CDKN1A overexpression) transfected with miR-NC1, miR-NC2, and miR-6077 upon treatment with PBS or CDDP (10 μM)/PEM (1 μM) for 14 days (C) or 48 h (D and E). (F) Dose-toxicity curves showing the viability of A549 cells (with or without CDKN1A overexpression) transfected with miR-NC1, miR-NC2, and miR-6077 upon treatment with PEM and oxaliplatin or carboplatin at the indicated concentrations for 48 h. Data are presented as the mean ± SD, n = 3 independent repeats. Unpaired, two-tailed t test; *p < 0.05, **p < 0.01, ***p < 0.001, ****p < 0.0001; ns, not significant.

for us to observe significant NRF2 alteration caused by miR-6077 in such conditions.

We did not observe the effect of miR-6077 on KEAP1-mediated ferroptosis after the stimulation of CDDP/PEM in A549 because of the G333C inactivating mutation. Therefore, to further verify the effects of KEAP1 mutation status on miR-6077-induced CDDP/PEM resistance, we designed an sgRNA specifically targeting the sequence around the mutated nucleotides and a repair template containing homology arms flanking the site of alteration, in which several nucleotides were substituted according to the wobble pairing rules, thereby avoiding accidental excision by sgRNA (Figure 5G). The sgRNA and the template sequence were co-transfected into A549 cells in the presence of Cas9. After clonal expansion, the efficiency of Cas9 cleavage and homology-directed repair (HDR)-mediated target modification was validated by both enzyme digestion reactions and Sanger sequencing (Figure 5G), thus indicating that the mutated KEAP1 in A549 had been successfully substituted by the wild-type sequence via Cas9-mediated genome engineering. In the edited A549 cells, similar assays were conducted, and we observed that miR-6077 induced upregulation of NRF2 and its downstream factors secondary to KEAP1 inhibition, just as we have observed in H1299 and H358, as well as exhibiting a protective effect against CDDP/PEM-induced ferroptosis (Figure S5I).

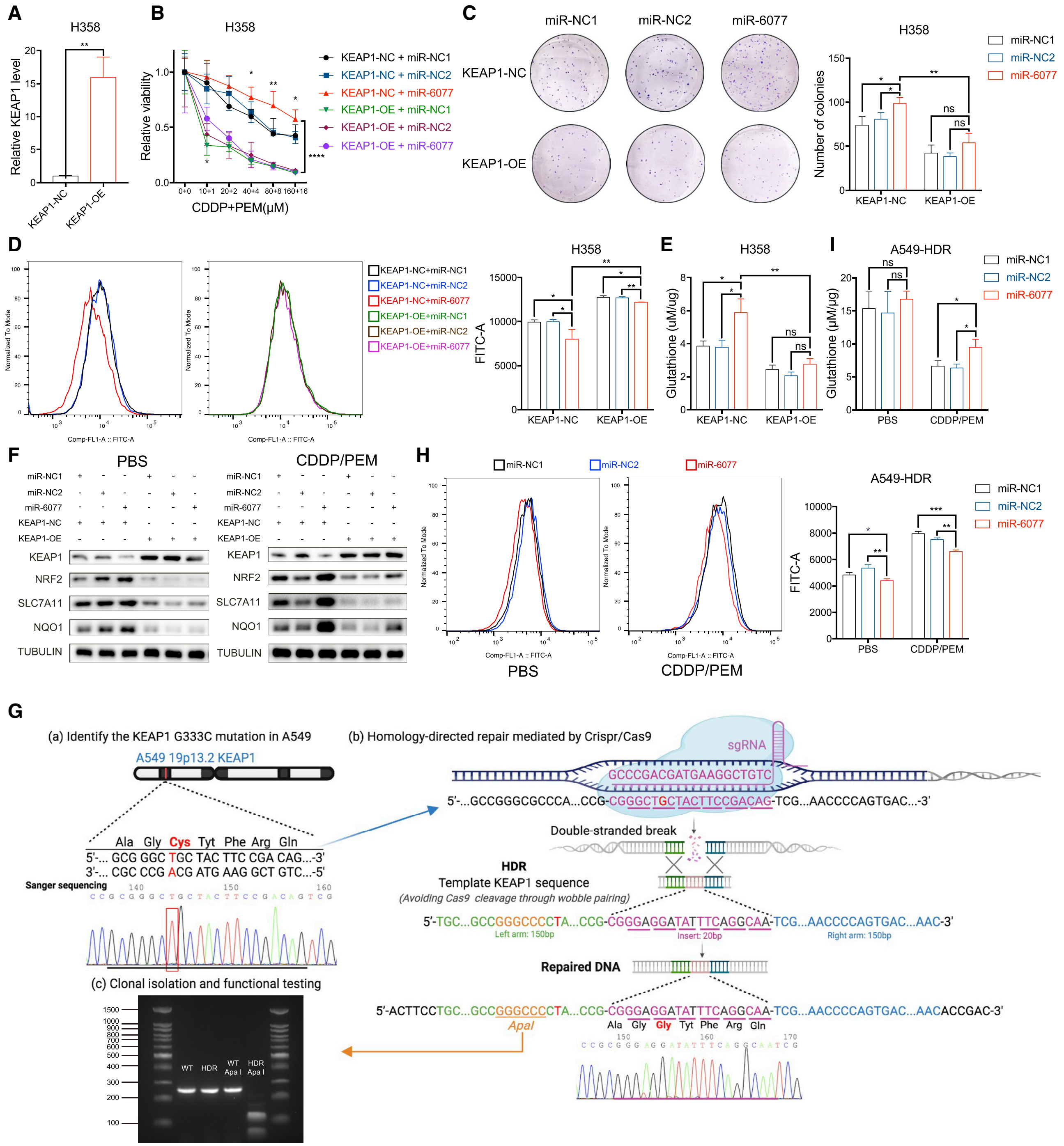

Figure 5. miR-6077 protects LUAD cells from CDDP/PEM-induced ferroptosis by directly targeting KEAP1

(A) Quantitative real-time PCR assay confirming the ectopic overexpression of KEAP1 in H358 cells. (B) Dose-toxicity curves showing the viability of H358 cells with or without KEAP1 overexpression transfected with miR-NC1, miR-NC2, and miR-6077 upon CDDP/PEM treatment at the indicated concentrations for 48 h. (C–F) Colony formation ability (C), lipid peroxidation (D), relative glutathione levels (E), and protein levels of KEAP1 and its downstream ferroptosis regulators (F) in H358 cells (with or without KEAP1 overexpression) transfected with miR-NC1, miR-NC2, and miR-6077 upon treatment with PBS or CDDP (20 μM)/PEM (2 μM) for 14 days (C) or 48 h (D–F). (G) Schematic outline of the CRISPR-Cas9-mediated homology-directed repair (HDR) in A549 cells. The mutated KEAP1 was replaced by a wild-type sequence. Agarose gel electrophoresis confirming the successful HDR in A549 cells, and the introduced repair template containing homology arms was digested by ApaI enzyme as expected. (H and I) Lipid peroxidation (H) and relative glutathione levels (I) in A549-HDR cells transfected with miR-NC1, miR-NC2, and miR-6077 upon treatment with PBS or CDDP (20 μM)/PEM (2 μM) for 48 h. Data are presented as the mean ± SD, n = 3 independent repeats. Unpaired, two-tailed t test; *p < 0.05, **p < 0.01, ***p < 0.001, ****p < 0.0001; ns, not significant.

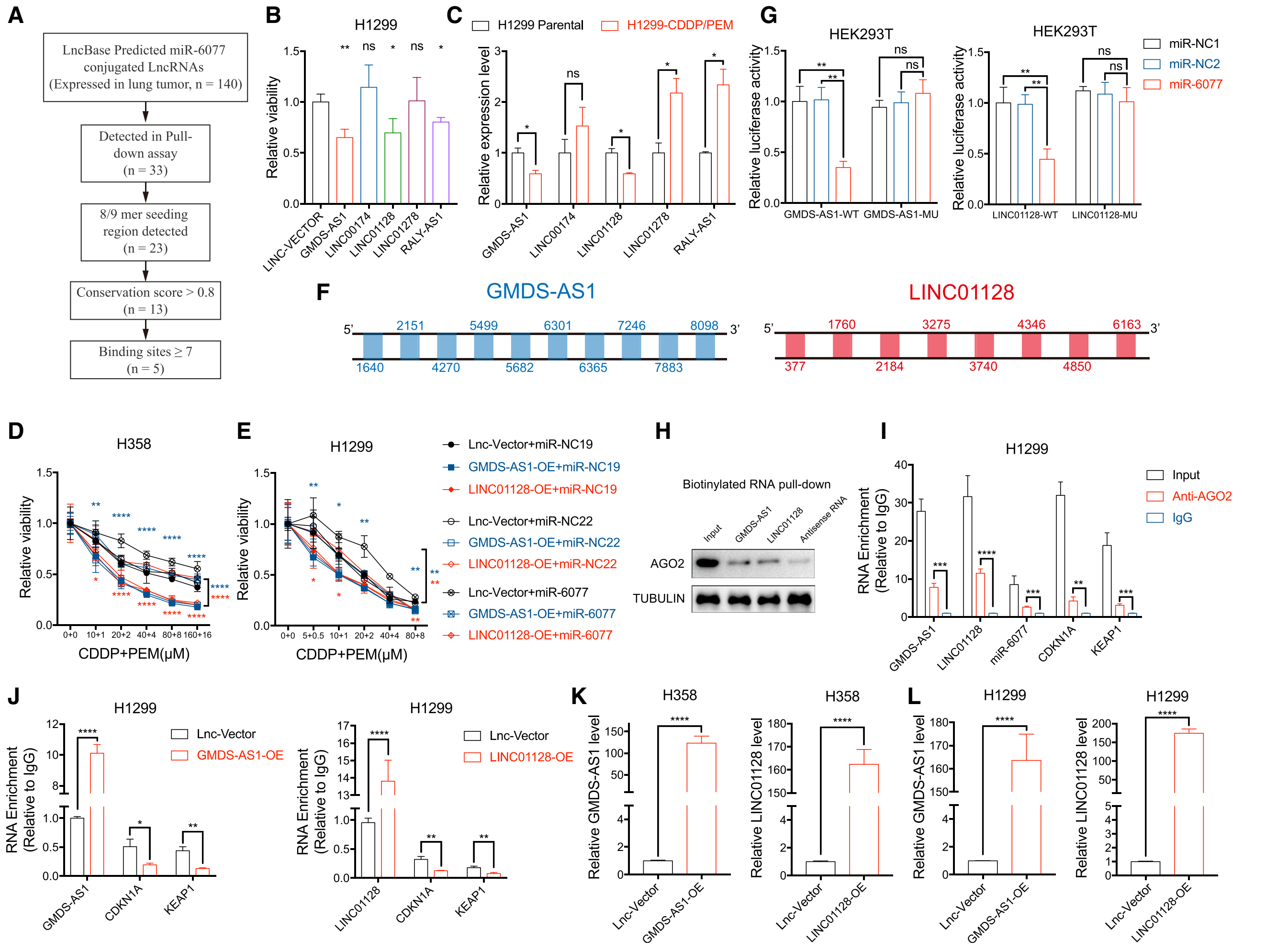

Figure 6. GMDS-AS1 and LINC01128 sensitize LUAD to CDDP/PEM and function as sponges for miR-6077 in LUAD

(A) Flowchart displaying the screening process of lncRNAs potentially interacting with miR-6077. (B) Bar plot showing that the overexpression of selected lncRNAs leads to altered sensitivity to CDDP (20 μM)/PEM (2 μM) treatment in H1299. (C) Relative expression levels of selected lncRNAs in H1299 and H1299-CDDP/PEM-resistant cells. (D and E) Dose-toxicity curves showing the viability of H358 (D) and H1299 (E) cells (with or without GMDS-AS1/LINC01128 overexpression) transfected with miR-NC1, miR-NC2, and miR-6077 upon CDDP/PEM treatment at the indicated concentrations for 48 h. Blue asterisks represent the significance of comparison between GMDS-AS1-overexpressing H1299 transfected with miR-NC and with miR-6077, while red asterisks represent that of LINC01128-overexpressing H1299. (F) The predicted target sites of miR-6077 in GMDS-AS1 and LINC01128. (G) Luciferase reporter plasmids containing wild- or mutant-type GMDS-AS1 (left) and LINC01128 (right) were co-transfected into HEK293T cells with miR-NC1, miR-NC2, or miR-6077. Bar plots exhibit the luciferase activity of the transfected cells. (H) RNA pull-down followed by western blotting showing a possible interaction between Ago2 and GMDS-AS1 or LINC01128. (I) RIP assay of the enrichment of GMDS-AS1, LINC01128, miR-6077, CDKN1A, and KEAP1 transcripts on Ago2 relative to IgG in H1299 cells. (J) RIP assay showing that overexpression of GMDS-AS1 or LINC01128 disrupts the interactions between miR-6077 and CDKN1A or KEAP1. Data are presented as the mean ± SD, n = 3 independent repeats. (K and L) Quantitative real-time PCR assay confirming the ectopic overexpression of GMDS-AS1 and LINC01128 in H358 (K) and H1299 (L) cells. Unpaired, two-tailed t test; *p < 0.05, **p < 0.01, ***p < 0.001, ****p < 0.0001; ns, not significant.

Collectively, from two perspectives, our findings demonstrated that miR-6077 confers LUAD cell CDDP/PEM resistance by inhibiting KEAP1, thus protecting cells against ferroptosis.

GMDS-AS1 and LINC01128 sensitize LUAD to CDDP/PEM by acting as a sponge for miR-6077

To further explore the upstream regulating mechanisms of miR-6077, considering that long non-coding RNAs (lncRNAs) can act as sponges for miRNAs, we screened five candidate lncRNAs by integrating the results from our miRNA pull-down and the online predictive tool LncBase (http://carolina.imis.athena-innovation.gr/diana_tools/web/index.php). The inclusion criteria and screening process are summarized in Figure 6A. Next, the potential functions of the candidates in the regulation of chemosensitivity were preliminarily assessed with cytotoxicity assays and qPCR, in which GMDS-AS1 and LINC01128 were selected for subsequent experiments, because they not only diminished H1299 resistance to CDDP/PEM but also exhibited lower expression in H1299-CDDP/PEM-resistant

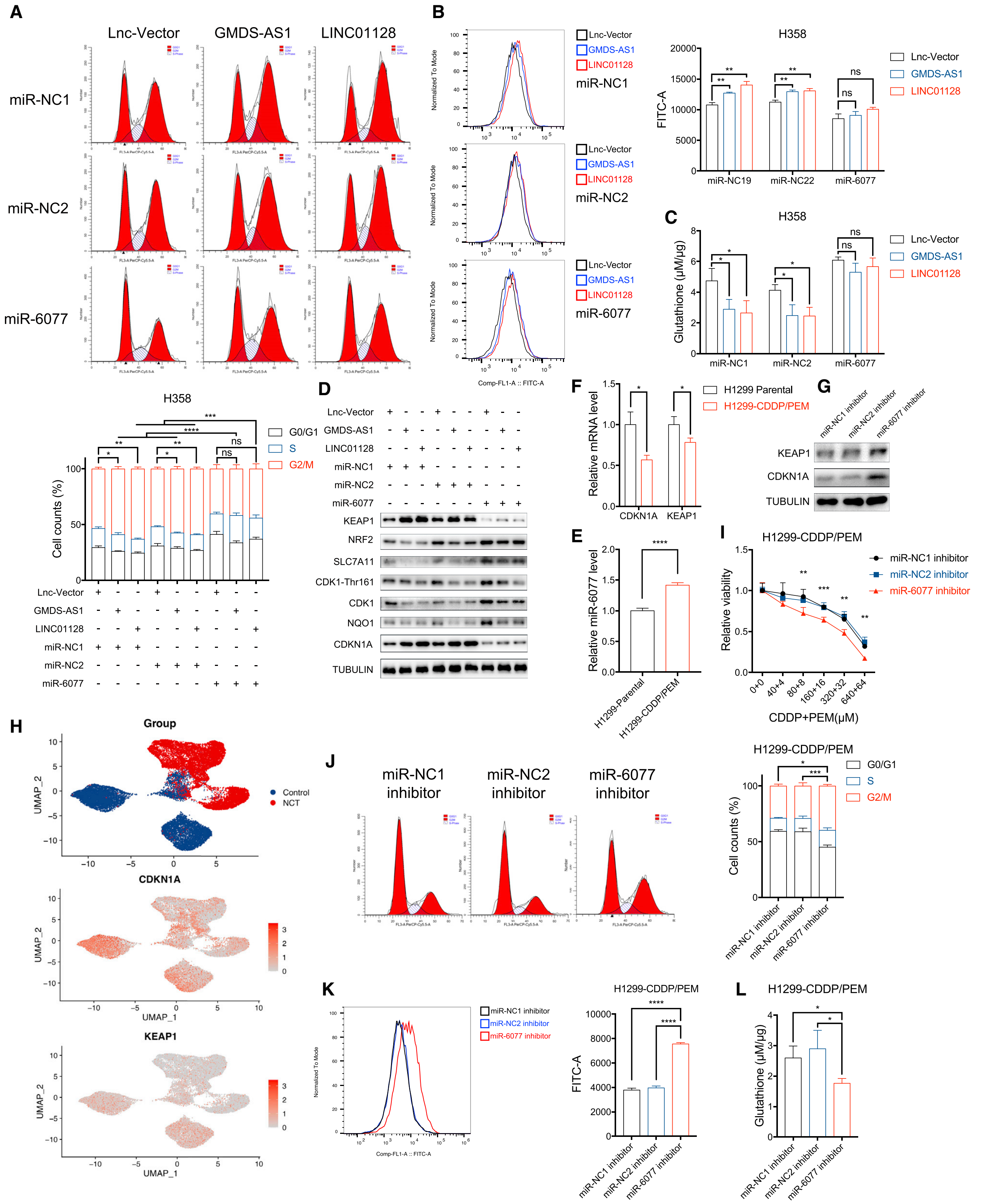

(legend on next page)

cells (Figures 6B and 6C). The results from cytotoxicity assays extended to H358, as well as other doses of CDDP/PEM (Figures 6D and 6E).

We found that the upregulation of GMDS-AS2 and LINC01128 decreased miR-6077 expression but increased CDKN1A and KEAP1 in different LUAD cell lines (Figure S6A), suggesting that the two lncRNAs may sensitize cells to CDDP/PEM at least partially by inhibiting miR-6077 expression in LUAD. Next, we tried to demonstrate the regulating relationship between the two lncRNAs and miR-6077 from the perspective of molecular structure. First, we constructed luciferase reporters containing wild-type or mutant GMDS-AS1 and LINC01128, to verify whether the two lncRNAs directly bound miR-6077 as a sponge. In the mutant sequences, all potential target sites were replaced to eliminate unexpected conjugation as thoroughly as possible (Figures 6F and S6B). Both the wild-type and the mutant plasmids were introduced into 293T cells together with the miR-6077 mimic. As expected, only the wild-type GMDS-AS1 and LINC01128 significantly attenuated the luciferase activity, whereas the mutant groups did not (Figure 6G). This finding elucidated that GMDS-AS1 and LINC01128 directly bind miR-6077 through their recognition sites. Moreover, to clarify the two lncRNAs' roles in the miR-6077-CDKN1A/KEAP1 axis, we performed lncRNA pull-down followed by western blotting. The results indicated possible interactions between lncRNAs and argonaute (Ago2), a key component of the RNA-induced silencing complex (RISC), which is involved in miRNA-mediated mRNA repression (Figure 6H). Supporting this conclusion, RNA binding protein immunoprecipitation (RIP) experiments were performed on H1299 cell extracts with antibodies specifically against Ago2. As shown in Figures 6I and 6J, GMDS-AS1, LINC01128, miR-6077, CDKN1A, and KEAP1 interacted with Ago2, and ectopic overexpression of the two lncRNAs resulted in enhanced enrichment on Ago2, but substantially decreased enrichment on CDKN1A and KEAP1. Collectively, GMDS-AS1 and LINC01128 serve as sponges and compete with downstream CDKN1A/KEAP1 for miR-6077-containing RISCs, thus preventing degradation of their mRNAs.

On the basis of the above findings, we stably introduced GMDS-AS1 and LINC01128 viral vectors into H358 and H1299 cells to explore the actual pathophysiological significance; the overexpression efficiency was validated by qPCR (Figures 6K and 6L). After exposure to CDDP/PEM, the overexpression of the two lncRNAs markedly increased the magnitude of G2/M arrest, lipid peroxidation, and GSH depletion, generating effects opposite those of miR-6077 (Figures 7A–7C and S7A–S7C). Likewise, in addition to CDKN1A and KEAP1 upregulation, transfection of the two lncRNAs dramatically decreased the levels of signaling molecules involved in the regulation of G2/M transition and ferroptosis, as discussed above (Figures 7D and S7D). In contrast, these phenomena were partially abrogated by ectopic restoration of miR-6077, whereas the effects of the two lncRNAs decreased significantly in the absence of miR-6077 (Figures 7A–7D and S7A–S7F). These results demonstrated that GMDS-AS1 and LINC01128 function by targeting miR-6077 as competing endogenous RNAs regulating CDKN1A and KEAP1 expression, thereby stimulating cell-cycle arrest in G2/M phase or ferroptosis when the LUAD cells were treated with CDDP/PEM and facilitating chemoresistance.

Knockdown of miR-6077 sensitizes LUAD to CDDP/PEM

When comparing the expression levels of miR-6077 in chemosensitive H1299 and chemoresistant H1299-CDDP/PEM cell lines, we observed upregulation in the latter (Figure 7E). In agreement with our results from tumor samples from patients with LUAD (Figure 1H), this finding further verified that miR-6077 is a feature of tumor cells that have developed CDDP/PEM resistance. Because its direct targets, CDKN1A and KEAP1, exhibited opposite trends in H1299-CDDP/PEM (Figure 7F), we transfected H1299-CDDP/PEM cells with miROFF-6077 inhibitor and observed the upregulation of CDKN1A and KEAP1 (Figure 7G). Furthermore, our findings from scRNA-seq supported this result. As shown in Figure 7H, the cell populations derived from CDDP/PEM-treated groups were characterized by greater expression of CDKN1A and KEAP1 in comparison with those that did not receive neoadjuvant chemotherapy. Because tumor samples were resected 2–4 weeks after neoadjuvant therapy, we inferred that the residual tumor cells were somewhat more resistant to CDDP/PEM than the untreated cells, given that the sensitive cells had been eliminated by the combination chemotherapy. Therefore, the upregulation of CDKN1A and KEAP1 in the treated groups could be explained by the long-term killing effect of CDDP/PEM chemotherapy, which led to a "natural selection" of the CDDP/PEM-resistant LUAD cells.

Moreover, in both the absence and the presence of miR-6077 inhibitors, the ferroptosis inhibitors could partially but not completely rescue CDDP/PEM-induced cell death in H1299-CDDP/PEM cells (Figure S7G). This phenomenon further suggested that, in addition to traditional programmed cell death mechanisms such as apoptosis

Figure 7. Overexpression of GMDS-AS1 and LINC01128 augments the magnitude of CDDP/PEM-caused G2/M arrest and lipid peroxidation by inhibiting miR-6077, and inhibiting miR-6077 results in chemosensitivity to CDDP/PEM in LUAD cells

(A–D) Cell-cycle analyses (A), lipid peroxidation (B), glutathione levels (C), and protein levels of CDKN1A/KEAP1 and their downstream molecules (D) in GMDS-AS1/LINC01128-overexpressing H358 cells transfected with miR-NC1, miR-NC2, and miR-6077 upon treatment with PBS or CDDP (20 μM)/PEM (2 μM) for 48 h. (E and F) Relative expression levels of miR-6077 (E) and CDKN1A and KEAP1 (F) in H1299 and H1299-CDDP/PEM-resistant cells. (G) Western blotting assay showing the protein levels of CDKN1A and KEAP1 in H1299-CDDP/PEM-resistant cells transfected with miR-NC1, miR-NC2 inhibitor, and miR-6077 inhibitor upon CDDP (20 μM)/PEM (2 μM) treatment for 48 h. (H) Single-cell sequencing data displaying the sample origin (top) and expression levels of CDKN1A (middle) and KEAP1 (bottom) of tumor cells derived from patients receiving or not receiving neoadjuvant CDDP/PEM treatment. (I–L) Dose-toxicity curves (I), cell-cycle analyses (J), lipid peroxidation levels (K), and relative glutathione levels (L) in H1299-CDDP/PEM-resistant cells transfected with miR-NC1, miR-NC2, and miR-6077 upon PBS or CDDP (20 μM)/PEM (2 μM) treatment for 48 h; *p < 0.05, **p < 0.01, ***p < 0.001, ****p < 0.0001; ns, not significant.

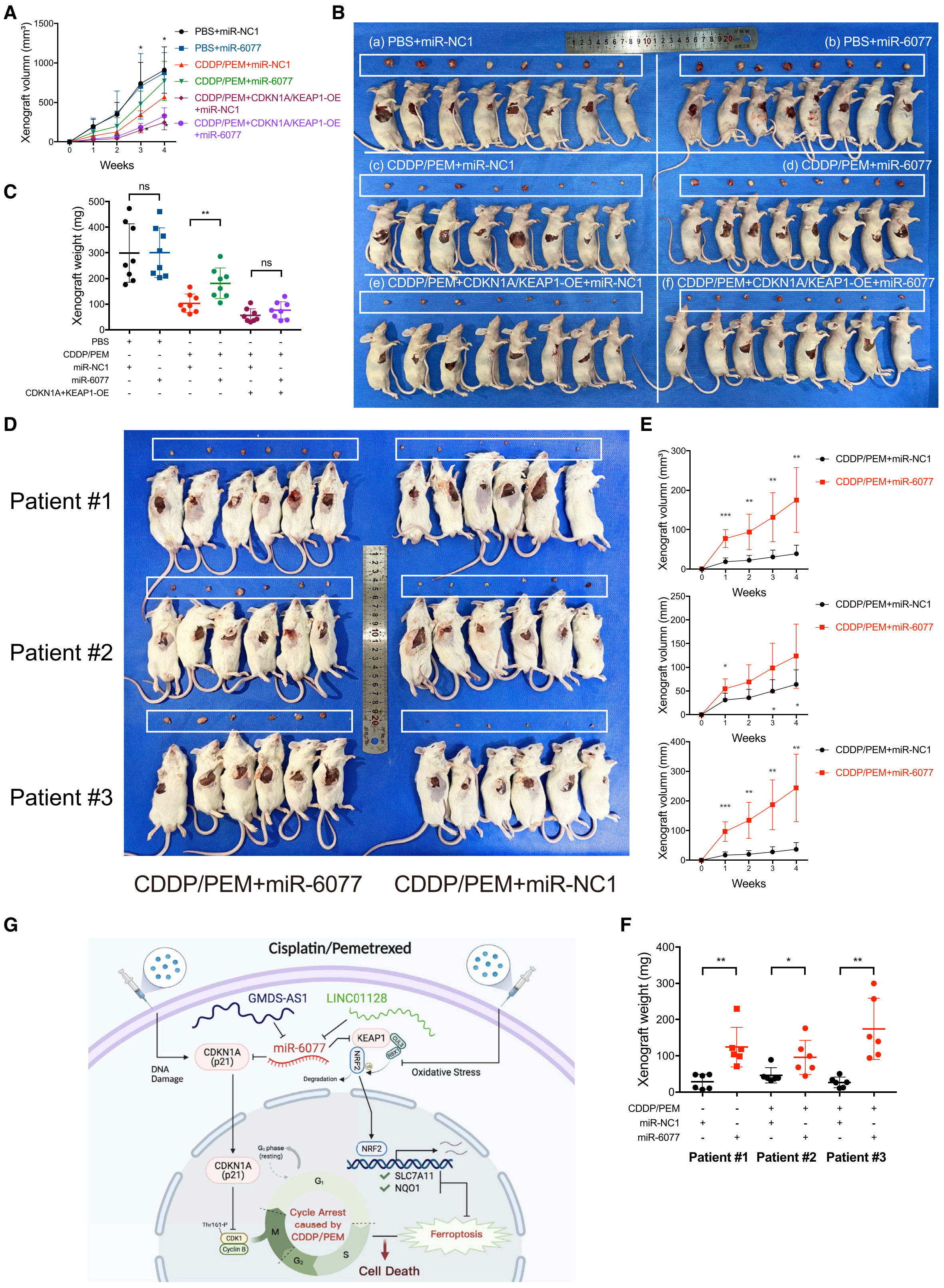

(legend on next page)

and necroptosis, ferroptosis also contributes to CDDP/PEM death. Meanwhile, knockdown of miR-6077 by the inhibitor decreased the chemoresistance in H1299-CDDP/PEM and aggravated cell-cycle arrest and ferroptosis induced by chemotherapy (Figures 7I–7L). These results are in line with our observations described above, and demonstrated that miR-6077 contributes to LUAD resistance to CDDP/PEM by regulating cell-cycle progression and ferroptosis by directly binding to CDKN1A and KEAP1.

miR-6077 promotes LUAD cell tolerance to CDDP/PEM *in vivo*

We next functionally validated the findings obtained above *in vivo*. Subcutaneously transplanted xenograft tumors derived from H1299 cells with intratumoral injection of miR-6077 agomir and intraperitoneal treatment with CDDP/PEM exhibited a dramatically lower tumor growth rate and tumor mass compared with those without CDDP/PEM treatment or with those treated with CDDP/PEM without miR-6077 administration (Figures 8A–8C). Meanwhile, we examined the effects of intratumoral injection of miR-6077 in H1299 cells stably overexpressing both CDKN1A and KEAP1. As shown in Figures 8A–8C, a significant reduction in tumor weight and proliferation rate was observed in the overexpressing cells after CDDP/PEM treatment, which diminished the protective effect generated by miR-6077. Together, these findings demonstrated that miR-6077 promotes LUAD cell tolerance to CDDP/PEM *in vivo* by targeting CDKN1A and KEAP1.

Furthermore, we also developed a patient-derived xenograft (PDX) model from three patients with LUAD and evaluated the effects of intratumorally injected miR-6077 or miR-NC on chemoresistance. The PDX mice with miR-6077 ectopic overexpression exhibited higher resistance to CDDP/PEM, thus suggesting that miR-6077 also confers chemoresistance in the microenvironment of human LUAD samples and may serve as a promising therapeutic target (Figures 8D–8F).

DISCUSSION

Chemotherapeutic agents such as CDDP and PEM are the mainstay of treatment for advanced LUAD. However, understanding the mechanism underlying the acquisition of chemoresistance, the major cause of treatment failure and tumor relapse, remains preliminary. In this research, among a panel of CRISPR-Cas9-screened miRNAs potentially associated with the CDDP/PEM desensitization phenotype, we studied the effects of miR-6077 and its mechanism of action in depth. A recent study has proposed that miR-6077 sensitizes LUAD cells to anlotinib by repressing the activation of GLUT1,[33] whereas its involvement in LUAD response and sensitivity to CDDP/PEM has never been reported. Mechanistically, on the basis of miRNA pull-down and RNA-seq, we observed that miR-6077 confers LUAD resistance to CDDP/PEM combination chemotherapy by directly binding the 3′ UTRs of CDKN1A and KEAP1, thereby mediating CDDP/PEM-induced cell death by initiating cell-cycle arrest and ferroptosis, respectively. Moreover, discovery of the two lncRNAs that serve as sponges for miR-6077, GMDS-AS1, and LINC01128 further contributed to an integrated understanding of the miR-6077-mediated signaling pathway.

Several studies have used loss- or gain-of-function screens to identify potential key molecules, including genes, miRNAs, lncRNAs, and circRNAs, that regulate tumor cell sensitivity to various chemotherapies.[34–36] However, most of them have utilized only short hairpin RNA or focused on small subsets of genes and have obtained distinct conclusions. The RNA-guided CRISPR-associated nuclease Cas9 provides an effective means of introducing targeted loss-of-function mutations at specific sites in the genome using the guiding effect of sgRNA, which can be generated and modified at large scale through array-based oligonucleotide library synthesis. The potential of this novel technique for pooled genome-scale functional screening has been verified in numerous studies in recent years.[37–40] Herein, we implemented an unbiased genome-scale CRISPR-Cas9 screening strategy to systematically capture the breadth of genetic features tightly associated with the LUAD response to CDDP/PEM, thus providing inspiration for future studies in this area. Through comprehensive functional experiments, we revealed that CDDP/PEM-resistant cells or tumor tissues are characterized by augmented miR-6077 expression, and it confers on LUAD cell lines resistance to CDDP/PEM both *in vitro* and *in vivo*, as well as leading to poorer prognosis and tumor relapse in patients with LUAD. These results are in line with the findings derived from CRISPR-Cas9 screening, thus further implicating the critical role of miR-6077 in this biological process (Figure 8G).

Emerging evidence indicates the induction of CDKN1A by p53 upon DNA damage caused by chemotherapy or radiotherapy in different types of cancer, which is in line with our results from RNA-seq and western blotting.[22,24,41–43] However, the exact function of CDKN1A in the regulation of cancer cell sensitivity to treatments that exert cytotoxicity by interfering with the stability and synthesis of genetic materials varies among tumor types.[18,29,44–46] This variation may be explained by the participation of CDKN1A as a master effector in multiple tumor-suppressor pathways that are tightly intertwined, such as cell-cycle arrest, apoptosis, senescence, and DNA repair.[24]

Figure 8. Ectopic overexpression of miR-6077 in H1299 cells confers resistance to CDDP/PEM *in vivo*

(A–C) Growth curves (A), image (B), and weights (C) of the xenograft tumors of sacrificed nude mice treated as indicated in each group (n = 8). H1299 cells with or without CDKN1A/KEAP1 overexpression were used to form the xenograft tumor subcutaneously. (D–F) Tumor cells derived from three LUAD patients were subcutaneously injected into NSG mice to form the PDX models, and the animals were treated with CDDP/PEM and miR-agomir as indicated for each group (n = 6). Growth image (D), curves (E), and weights (F) of the PDX tumors are shown. (G) A schematic diagram indicating the mechanism by which miR-6077 confers LUAD chemoresistance by mitigating CDDP/PEM-induced G2/M arrest and ferroptosis by specifically targeting CDKN1A and KEAP1, respectively. CDDP plus PEM is the predominant therapeutic regimen for LUAD, whereas chemoresistance greatly limits its curative potential. We found that miR-6077 promotes chemoresistance by targeting CDKN1A-mediated cell-cycle arrest and KEAP1-mediated ferroptosis. Our discovery provides a novel therapeutic strategy for clinical practice.

Through bioinformatic analysis based on both RNA-seq and scRNA-seq, we observed the activation of several biological processes involved in cell-cycle progression. It is widely accepted that CDKN1A induces cell-cycle arrest in different phases and finally leads to growth inhibition by restraining the expression and function of various CDKs,[24,42,47] notably causing G2/M arrest by blocking the activating Thr161 phosphorylation on CDK1, also known as Cdc2, a kinase required for entry into mitosis.[23] As implied by our observations, the hypothesis that CDKN1A suppresses tumors by triggering G2/M cell-cycle arrest in response to chemo-stimuli was verified by our cytotoxic and cell-cycle tests, as well as corresponding rescue assays. Collectively, on the basis of the above-mentioned studies and the direct targeting relationship between miR-6077 and CDKN1A, our findings demonstrated that miR-6077 desensitizes LUAD cells to CDDP/PEM by targeting CDKN1A and thus attenuating the consequent G2/M arrest. However, several researchers believe that cell-cycle arrest caused by elevated CDKN1A provides sufficient time for DNA repair and enables cell survival.[48] Therefore, when assessing the role of CDKN1A-induced cell-cycle arrest in cancer cell sensitivity, the balance between DNA repair and proliferation inhibition should be considered.

Ferroptosis refers to an iron-dependent form of regulated cell death induced by unrestricted lipid peroxidation and subsequent plasma membrane rupture.[26,27] Like other forms of regulated cell death, ferroptosis is tightly associated with multiple physical and pathological processes in both normal and cancer cells. For example, Lei et al. have indicated that ionizing radiation causes ferroptosis through not only the production of ROS and lipid peroxidation, but also the induction of ACSL4 expression.[16] Given that, in addition to DNA damage described above, CDDP and PEM exert their cytotoxicity by triggering the excessive generation of ROS,[13–15,49] our research further confirmed that CDDP/PEM treatment may induce ferroptosis, a conclusion partially consistent with findings from a recent study on CDDP's role in the deletion of glutathione.[50] Furthermore, studies increasingly suggest that ferroptosis affects the efficacy of chemotherapy and radiotherapy,[26,51,52] thus raising the intriguing question of whether deficient ferroptosis might result in resistance to the combination chemotherapy of CDDP plus PEM in patients with LUAD.

The role of the KEAP1-NRF2 pathway in the regulation of redox and metabolic homeostasis has been explored for decades.[31] Recently, its critical role in the manipulation of ferroptosis has drawn attention. Generally, NRF2 is a key regulator of the antioxidant response and drug detoxification. Under homeostasis, low levels of NRF2 are maintained by KEAP1, which recruits a cullin 3-containing E3 ubiquitin ligase complex to NRF2, thus resulting in proteasome-mediated degradation.[53,54] Under oxidative and toxic stress conditions, however, NRF2 is released from KEAP1 and translocated to the nucleus, where it heterodimerizes with partner proteins such as Maf and subsequently initiates the transcription of a series of ferroptosis-related genes, such as SLC7A11, and genes encoding antioxidant response elements, such as NQO1,[30] by binding to their promoter regions, thus protecting cells against the cytotoxic effects of chemotherapy. Several studies have demonstrated that KEAP1/NRF2 signaling plays an essential role in tumor cell sensitivity to chemotherapy, because NRF2 activation or KEAP1 dysfunction caused by deletion or epigenetic modifications increases resistance to CDDP, particularly in LUAD, in which genes involved in the KEAP1/NEF2 pathway are mutated in 22% of patients.[55–57] Notably, the A549 cell line, which is widely used in lung tumor research, is characterized by a loss-of-function mutation at G333C. Therefore, it is reasonable to make the deduction that tumor cell response to CDDP-based chemotherapy might be overcome by targeting the KEAP1/NRF2-mediated disturbance of ferroptosis, as presented and verified in this study. Our findings indicated that LUAD cell resistance to CDDP/PEM and mitigated ferroptosis, driven by ectopically expressed miR-6077, was rescued by restoration of the direct target KEAP1. Furthermore, with the assistance of CRISPR-Cas9-mediated HDR, we replaced the mutated KEAP1 in A549 cell lines with the wild type, and observed the expected effects of miR-6077 on ferroptosis-related markers, which were absent in the KEAP1-mutated genetic background. Notably, no researchers have previously integrated these three key elements, referring to ferroptosis, KEAP1, and CDDP/PEM resistance, altogether, while our study filled this gap.

lncRNAs have been well characterized to function as miRNA sponges in the manipulation of diverse cellular processes.[58,59] In the present study, we confirmed that GMDS-AS1 and LINC01128 are primarily located in the cytoplasm and compete with downstream CDKN1A or KEAP1 for miR-6077-containing RISCs. Previous studies have reported the indispensable roles of these two lncRNAs in tumor development and progression in different tumor types.[60–62] On the basis of the above-mentioned binding between miR-6077 and these two lncRNAs, as well as the functional experiments measuring cytotoxicity, cell-cycle percentage, and ferroptosis, we determined that GMDS-AS1 and LINC01128 sensitize LUAD cells to CDDP/PEM by negatively regulating the miR-6077-CDKN1A/KEAP1 axis.

In summary, this study provides the first reported comprehensive evidence that miR-6077 drives resistance to CDDP/PEM and is a prognostic biomarker for patients with LUAD receiving combination chemotherapy. We elucidated that specific targeting of CDKN1A and KEAP1 is the molecular mechanism through which miR-6077 mitigates G2/M arrest and ferroptosis induced by exposure to CDDP/PEM. Furthermore, GMDS-AS1 and LINC01128 function as miRNA sponges, thereby generating effects opposite those of miR-6077 in the regulation of LUAD chemoresistance. Therefore, these findings have significant implications regarding our understanding of the development of MDR of LUAD, and targeting the diverse molecules described above may provide novel therapeutic strategies for LUAD insensitive to CDDP/PEM treatment.

MATERIALS AND METHODS

Cell lines

All cells were cultured in a 37°C incubator in a humidified 5% CO_2 atmosphere. The human LUAD cell line A549, H358, and human embryonic kidney 293 (HEK293T) cells were cultured in high-glucose

Dulbecco's modified Eagle's medium (DMEM; Hyclone, Logan, UT, USA) supplemented with 10% fetal bovine serum (Every Green, Zhejiang, China) and 100 U/mL penicillin/streptomycin/amphotericin B (Sangon Biotech, Shanghai, China). The LUAD cell line H1299 was maintained in RPMI 1640 medium (Hyclone, Logan, UT, USA) supplemented with 10% fetal bovine serum (Every Green, Zhejiang, China) and 100 U/mL penicillin/streptomycin/amphotericin B (Sangon Biotech). The CDDP/PEM-resistant subline H1299-CDDP/PEM was established in our laboratory by culturing H1299 cells in gradually increasing concentrations of CDDP/PEM (up to 2 and 0.2 μM, respectively) and was cultured in complete RPMI 1640 medium containing 2 μM CDDP and 0.2 μM PEM for the maintenance of resistance. All primary cell lines were purchased from the Chinese Academy of Science Cell Bank as we previously described.[63]

Compounds

CDDP (T1564), PEM (T6226), oxaliplatin (T0164), carboplatin (T1058), erastin (T1765), ferrostatin-1 (T6500), and deferoxamine (DFO; T1637) were purchased from Topscience (USA). The first three compounds were dissolved in PBS (Beyotime, Shanghai, China) and the last three in DMSO (Beyotime), according to their solubility, and then stored at −20°C.

CRISPR-Cas9 screening

The RNA-guided CRISPR-Cas9 knockout screening system was adapted from the sequences published by Zhang and colleagues,[37] which contain a pooled genome-wide sgRNA library targeting 20,060 protein-coding genes and 1,854 miRNAs (six independent sgRNAs per gene). The library of sgRNAs was obtained from Genechem (Shanghai, China). For subsequent genome-wide screening, the optimal volumes of lentivirus (MOI), puromycin, and CDDP/PEM were determined in the A549 cell line through multi-point dose-response assays in advance.

After cell culture and amplification, 1×10^8 A549 cells were resuspended in 50 mL fresh medium containing 2% Hitrans G P transfection reagent (Genechem) and lentivirus sgRNA library at an MOI of 0.8, and seeded in a 245 × 245 mm cell dish (Corning, NY, USA). The next day, the culture medium was replaced with fresh medium. At 72 h after transduction, 1.2 μg/mL puromycin was added into the dish. After puromycin selection for 48 h, 3×10^7 cells were trypsinized and harvested, which represented day 0 (D0), indicating the baseline level of sgRNA. The remaining cells were seeded into new dishes at the density of 3×10^7 cells per dish. After 24 h, the cells were continuously exposed to 2 μM CDDP plus 0.2 μM PEM or PBS. Cells were cultured and passaged as needed to ensure a confluence lower than 90%. After 7 or 14 days of treatment, at least 3×10^7 cells from each group were harvested for subsequent processing.

Next, genomic DNA from the five groups (D0, D7-PBS, D7-CDDP/PEM, D14-PBS, and D14-CDDP/PEM) was extracted with a TIANamp Genomic DNA kit (TIANGEN, Beijing, China) and quantified according to the manufacturer's protocol. PCR amplification was performed with 2× Seeley Max Master Mix (Seeley, Shanghai, China) with primers specific to the genome-integrated lentiviral vector backbone sequence. The PCR products were purified and preprocessed (conjugation with primers specific to Illumina sequencing) with a NEBNext Ultra DNA Library Prep Kit for Illumina (NEB, USA), qualified by Agilent 2100 (Agilent Technologies, Palo Alto, CA, USA), and quantified with Qubit 2.0 (Invitrogen, Carlsbad, CA, USA). Finally, the samples were subjected to paired-end sequencing on the Illumina HiSeq platform (Illumina, San Diego, CA, USA). Raw sequencing reads were processed and analyzed in R (R Foundation for Statistical Computing, Vienna, Austria). The *limma* package was used to evaluate differential sgRNA enrichment before (D0) and after (D7 or D14) treatment, shown as $\log_2$ (fold change). ΔlogFC indicates [$\mathrm{logFC}_{(\mathrm{CDDP/PEM})} - \mathrm{logFC}_{(\mathrm{PBS})}$] for D7 or D14.

miRNA mimic/inhibitor transfection

The sequences of miRNA mimics (RiboBio, Guangzhou, China) and inhibitors (RiboBio) are listed in Table S1. We confirmed that the miR-NCs, which were derived from cel-miR-293b-5p and cel-miR-67-3p, did not specifically target any mRNAs in the human genome. The cells were seeded into six-well plates at 60%–80% confluence. The next day, the medium was replaced with fresh medium containing miRNA mimics (150 μM) or inhibitors (200 μM) and Lipo8000 (Beyotime) as the transfection reagent. The cells were harvested for subsequent analyses 48 h after transfection.

Cell viability assays

For cytotoxicity assays, 5,000 cells per well were seeded in quintuplicate in 96-well plates and incubated for 24 h. Cells were treated with different doses of chemotherapy drugs for 48 h as required. For cell proliferation assays, the cells were seeded at a density of 1,000 cells per well and incubated for 0, 24, 48, 72, 96, and 120 h at 37°C. Cell viability was measured with a CellTiter-Lumi kit (Beyotime) according to the manufacturer's instructions. For high-content analysis, GFP-overexpressing A549 cells were treated as indicated, and the cell proliferation was dynamically monitored according to corresponding fluorescence intensity using Celigo cytometer (Cyntellect, San Diego, CA, USA) equipped with a 4-megapixel CCD camera with an F-theta scan lens.

Colony formation assays

Cells were seeded into six-well plates at a density of 500 cells for A549 and 3,000 cells for H1299 and H358 per well in logarithmic growth phase. After being cultured in complete culture medium for 14 days, the cells were fixed with 4% methanol for 30 min and stained with 1% crystal violet.

Quantitative real-time PCR

RNA extraction and quantitative real-time PCR were performed as previously described.[63] Total RNA was extracted from tissues or cells with TRIzol reagent (TIANGEN), and cDNA was synthesized with a Hifair II First-Strand cDNA Synthesis Kit (gDNA Digester Plus, YEASEN, China). qPCR was performed with a Hifair III One-Step RT-qPCR SYBR Green Kit (YEASEN), and triplicate samples were

run on an ABI QuantStudio 5 real-time PCR system (Thermo Fisher, USA). The threshold cycle (Ct) values for each gene were normalized to those of GAPDH as an endogenous calibrator, and the $2^{-\Delta\Delta Ct}$ method was used for quantitative analysis. The primers used were synthesized by Sangon Biotech and are listed in Table S2. To confirm the expression of miR-6077, we isolated RNA with a miRcute miRNA Isolation Kit (Qiagen, Germantown, MD, USA), and the reverse transcription and qPCR were performed with a miRcute Plus miRNA First-Strand cDNA Kit and miRcute Plus miRNA qPCR Kit (SYBR Green, Qiagen), respectively. Small nuclear RNA (U6) was used as an endogenous calibrator.

Patients and tumor specimens

In total, tumor samples of 31 patients with LUAD were obtained from the Department of Thoracic Surgery, Zhongshan Hospital, Fudan University, 20 of which were used to assess the correlation between patients' resistance to CDDP/PEM and miR-6077 levels (denoted cohort A; all patients received treatment with standard combination chemotherapy of CDDP plus PEM before surgery), and 8 were subjected to 10× scRNA-seq (denoted cohort B; 4 received preoperative CDDP/PEM and 4 did not). The remaining 3 were used to establish a PDX model. The patients underwent curative resection during 2020–2021. All patients provided written informed consent to conduct genomic studies in accordance with the ethical principles of the Declaration of Helsinki. All pulmonary resections were performed by experienced thoracic surgeons in our institution, and resected tumors were all labeled in the operating theater and reviewed by at least two qualified pathologists to confirm the diagnosis of LUAD through hematoxylin and eosin-stained sections and immunochemical analysis. Patients in cohort A were further defined as sensitive (complete response or partial response) or resistant (stable disease or progressive disease) according to RECIST edition 1.1. The baseline information for the patients involved in this study is summarized in Table S3. This study was approved by the Ethics Committee of Zhongshan Hospital, Fudan University (B2022–180).

Fluorescence-activated cell sorting

The tumor cells were isolated from the bulk samples obtained from patients in cohort A by fluorescence-activated cell sorting, as described in our previous publication.[64] Briefly, after surgical resection, samples were immediately collected and then dissociated into a single-cell suspension with a Tumor Dissociation Kit (Miltenyi Biotec, Gladbach, Germany). Single cells were resuspended and incubated with 20 μg/mL human IgG (Sigma-Aldrich, St. Louis, MO, USA) for 15 min to block non-specific antibody binding. Subsequently, cells were incubated with fluorescently labeled primary antibody (Table S4) for 30 min on ice, and this was followed by stained cell quantification and sorting on a FACSAria III instrument (BD Biosciences, USA). The sorted cells were subsequently subjected to qPCR.

Pull-down assays with biotinylated miRNA

The capture of miR-6077-bound competing endogenous RNAs in pull-down assays was performed as previously described.[65] The biotin-labeled miR-6077 mimic probe was synthesized by RiboBio. A549 cells were transfected with biotinylated miR-6077 (150 nM) and harvested, lysed, and sonicated at 24 h after transfection. The biotin-coupled RNA complex was pulled down by incubating the cell lysates with streptavidin-coated magnetic beads (Beyotime) on a rotator at 37°C for 1 h. Next, the bound RNA was washed and purified with a RNeasy Mini Kit (Qiagen) and then subjected to quantitative real-time PCR or RNA-seq.

RNA sequencing and bioinformatic analysis

RNA obtained from miR-6077-transfected cells or pull-down assays was subjected to library construction (performed with Agilent2100/2200 and Qubit instruments as described above) and sequencing (Illumina). We used TopHat (v.2.0.13)[66] and hisat2[67] to map the clean reads to each gene and normalized the raw data to Fragments Per Kilobase of exon model per Million mapped fragments (FPKM) for subsequent analyses. Bioinformatics analyses were performed as previously described.[68,69] DEGs were identified with the *limma* package, which implements an empirical Bayesian approach to estimate gene expression changes by using the moderated t test.[70] $|\log FC| > 0.5$ and $p < 0.05$ were considered cutoff criteria to screen for DEGs. Functional enrichment analyses of the detected DEGs were performed with the *clusterProfiler* package. Gene Ontology (GO) and Kyoto Encyclopedia of Genes and Genomes (KEGG) terms were identified with a cutoff of $p < 0.05$. We also identified pathways that were up- or downregulated in preoperatively CDDP/PEM-treated or untreated patients with GSEA.[71] Gene sets for analysis were obtained from the MSigDB database: M14052 for cell-cycle G2/M phase transition and M39768 for ferroptosis.

Western blotting

Western blotting was performed according to standard procedures as previously described.[63] Proteins were extracted from cells with RIPA buffer (Beyotime) with protease and phosphatase inhibitor cocktail (Beyotime) and quantified with an Enhanced BCA Protein Assay Kit (Beyotime). Protein concentrations were determined with a Bicinchoninic Acid Protein Assay Kit (YEASEN) and were then boiled in 5× SDS-PAGE loading buffer (EpiZyme, Shanghai, China) for 10 min at 100°C. Next, proteins were separated by SDS-PAGE and transferred to polyvinylidene fluoride membranes (Merck-Millipore, Burlington, MA, USA) (constant current 0.25–0.30 A, 70–90 min). The membranes were blocked with 5% non-fat milk for 1 h and then incubated with specific primary antibodies for 12 h at 4°C. After the membranes were washed three times with Tris-buffered saline-Tween solution, the secondary antibody dilutions were incubated with the membranes at room temperature for 1 h. Finally, the protein bands were visualized with a Moon chemiluminescence reagent kit (Beyotime). Tubulin served as the internal reference. All antibodies used in this research are listed in Table S4.

Dual-luciferase reporter assays

The 3′ UTR sequences of CDKN1A and KEAP1, as well as GMDS-AS1 and LINC01128, or the corresponding mutated sequences on the predicted target sites, were cloned into the phy-811@7 dual luciferase reporter vector (Hanyin Technology, Shanghai, China). All

constructs were verified by direct sequencing. HEK293T cells were seeded on a polylysine-treated 24-well plate at 60%–80% confluence. After 24 h, the cells were co-transfected with 200 nM miR-6077 mimics and 400 ng of the wild-type or mutant plasmids constructed as above with Lipo8000 (Beyotime) as the transfection reagent. At 48 h after transfection, the cells were collected, and dual-luciferase reporter assays were conducted with a Luciferase Reporter Gene Assay Kit (Beyotime), as directed by the manufacturer. Luciferase activity was detected with a Microplate spectrophotometer (Bio-Rad, Hercules, CA, USA).

Single-cell sequencing

The detailed information for the eight LUAD tumor samples (cohort B) obtained from our institute is summarized in Table S2. Tissue processing, scRNA-seq, and data analyses were performed as previously described.[72,73] Briefly, after surgical resection, samples were immediately collected, dissociated into single-cell suspensions, and subjected to scRNA-seq on the Illumina sequencing platform. The sequencing data analyses, including quality control, data normalization, highly variable feature selection, scaling, dimension reduction, and uniform manifold approximation and projection (UMAP), were performed with the *Seurat* package according to standard procedures.[74] We annotated the separated cell populations according to the CellMarker dataset and our previous studies.[72,73] Finally, the clusters representing tumor cells were extracted and labeled with their sample type of origin for downstream analysis.

Cell-cycle analysis

The cells were seeded in six-well plates at 50%–60% confluence and treated as indicated 24 h after plating. After 48 h, the cells were harvested and fixed in 70% ice-cold ethanol overnight and then stained with propidium iodide in the presence of RNase A (Beyotime). Fluorescence intensity was measured with an Accuri 6 cytometer (BD Biosciences). The percentages of cells in G0/G1, S, and G2/M phases were analyzed in ModFit LT software (Verity Software House, Topsham, ME, USA).

Lipid peroxidation assays

After incubation with various treatments for 48 h, the cells were harvested and washed with PBS. Next, the cells were incubated in fresh medium containing 4 μM BODIPY 581/591 C11 dye (Thermo Fisher, USA) for lipid peroxidation measurements at 37°C in a humidified 5% CO_2 atmosphere. After 30 min of incubation, the cells were washed with PBS, and the lipid peroxidation levels were assessed with an Accuri 6 cytometer. The results were analyzed in FlowJo software (TreeStar, Woodburn, OR, USA).

Determination of reduced GSH levels

The treated cells were harvested to determine cell number, and nearly 6×10^5 live cells from each sample were transferred to new tubes, washed in PBS, and centrifuged at 1,200 rpm at 4°C for 5 min. The cell pellets were resuspended in 60 μL protein removal solution, thoroughly mixed, and incubated at −196°C (liquid nitrogen) and 37°C sequentially twice for fast freezing and thawing and then incubated at 4°C for 5 min and centrifuged at $10,000 \times g$ for 10 min. The supernatant was extracted to determine the amount of GSH in the sample. This assay was conducted with a GSH and GSSG Assay Kit (Beyotime) according to the manufacturer's protocol.

Transmission electron microscopy

Treated cells cultured in 6-cm dishes were fixed with a solution containing 2.5% glutaraldehyde. After being washed in 0.1 M phosphate buffer (pH 7.4) three times, cells were postfixed with phosphate buffer containing 1% osmic acid, followed by washing in 0.1 M phosphate buffer (pH 7.4) another three times. After dehydration and embedding, samples were incubated in a 60°C oven for 48 h. Ultrathin sections were prepared and stained with lead citrate and uranyl acetate. After drying overnight, the sections were examined with a Hitachi transmission electron microscope (Hitachi, Japan).

Homology-directed repair with the CRISPR-Cas9 system

This assay was performed according to the protocol provided by Ran et al.[75] Briefly, sgRNA specifically targeting the sequence around the mutated nucleotides was generated with the Crispick online tool (https://portals.broadinstitute.org/gppx/crispick/public). The final sequences of the sgRNA and repair template are listed in Table S5. sgRNA, repair template, and Cas9 expression plasmid were synthesized by Genechem. For subsequent selection, a puromycin-resistance gene was introduced into the plasmid encoding Cas9, and an ApaI restriction enzyme cutting site was introduced into the sequence of the repair template.

First, A549 cells were plated in six-well plates at 4×10^5 cells per well and transfected with siRNAs for Ku70, Lig4, and XRCC4 on the next day to inhibit non-homologous end joining, thus increasing the efficiency of the HDR.[76–78] The siRNAs were purchased from Hanyin Technology (Shanghai, China), and the sequences are listed in Table S5. After 24 h, the transfected A549 cells were further simultaneously co-transfected with the three plasmids containing the abovementioned sgRNA, repair template, and Cas9 and then selected with puromycin. Lipo8000 was used as the transfection reagent in these two steps.

At 48 h after transfection and selection, cells were isolated through serial dilution to form the clonal cell populations. After 2 weeks' expansion, to assess the efficiency of Cas9 cleavage and HDR-mediated target modification, we extracted genomic DNA from the transfected cell populations and amplified it using the Taq PCR Master Mix (BBI Life Sciences, Shanghai, China). The PCR products were incubated with ApaI enzyme (Takara, Kusatsu, Japan) overnight for thorough digestion. Next, the undigested and digested genomic DNA was separated with 2% agarose gel electrophoresis (BIOWEST, France) with TAE (Sangon Biotech) running buffer at 120 V for 30 min. The bands were examined via UV irradiation, and the clonal cell populations exhibiting multiple digested bands were subjected to Sanger sequencing (Sangon Biotech) for final confirmation of the effective HDR-mediated target modification. The cell clones in which the mutated

KEAP1 was successfully replaced by the repair template were maintained for further selection.

Lentivirus transduction

For the establishment of cell lines with stable CDKN1A or KEAP1 overexpression, the lentivirus vectors for CDKN1A, KEAP1, GMDS-AS1, and LINC01128, and corresponding negative control sequences, were obtained from Hanyin Technology. A total of 5×10^4 cells were seeded into 12-well plates. After 24 h, lentivirus was added at an MOI of 10, cells were cultured in complete medium containing 5 μg/mL polybrene for 12–16 h, and the culture medium was replaced with fresh medium. At 72 h after transduction, the cells were subsequently propagated in selection medium containing 2.5 μM puromycin (Hanyin Technology).

RNA immunoprecipitation assays

RIP assays were performed with a Magna RIP RNA-Binding Protein Immunoprecipitation Kit (Millipore, MA, USA) according to the manufacturer's instructions. Briefly, the cells were harvested and lysed with RIP lysis buffer and then incubated with RIP buffer supplemented with magnetic beads conjugated with human antibodies to Ago2 and normal mouse IgG (negative control; Millipore) overnight at 4°C. Next, the co-precipitated RNA was purified as directed by the manufacturer and analyzed by qPCR.

Cell-line-derived nude mouse tumor xenograft model

All animal studies were conducted in compliance with the policies of the animal ethics committee of the Fudan University. Four-week-old male BALB/c nude mice were purchased from the Laboratory Animal Center of Shanghai Medical college and maintained under pathogen-free conditions. For each nude mouse, 4×10^6 H1299 cells subjected to various treatments were resuspended in 100 μL PBS and subcutaneously injected into the right flank. One week after implantation, cohorts of tumor-bearing mice were treated with cycles of miR-agomir and CDDP/PEM every 4 days. On the first days of the administration cycles (D1/4), 50 μL of the miR-6077 or control agomir (2 nmol) was intratumorally injected. The chemotherapeutic drugs were administered as 3 mg/kg CDDP and 0.3 mg/kg PEM subperitoneally on the second day of the cycle (D2/4). Tumor sizes were measured with Vernier calipers weekly, and the tumor volume was calculated as $(\text{length} \times \text{width}^2)/2$. The xenograft tumors were harvested for subsequent immunohistochemistry 4 weeks after implantation.

For the establishment of a PDX model, three LUAD tumor samples were cut into 3- to 4-mm pieces and subcutaneously transplanted within 4 h after surgical removal into 6-week-old female severely immunodeficient M-NSG mice (Model Organisms, Shanghai, China). When the tumor size exceeded 1,000 mm^3, the animals were sacrificed, and the tumors were removed. Next, the dissected xenografts were mechanically and enzymatically disaggregated into single-cell suspensions, as described above. Patient-derived cells (5×10^6) were implanted into the right flanks of M-NSG mice and treated with miR-agomir and CDDP/PEM as described above.

Statistical analysis

All experiments were performed in at least triplicates. Unpaired Student's t tests were performed to compare continuous variables between two groups. The results are presented as means, and the error bars represent the standard deviation unless stated otherwise. All statistical analyses were conducted in GraphPad Prism software (7.0) and R software. The p values were all two-tailed, and $p < 0.05$ was considered significant: *$p < 0.05$, **$p < 0.01$, ***$p < 0.001$, ****$p < 0.0001$; ns, not significant.

SUPPLEMENTAL INFORMATION

Supplemental information can be found online at https://doi.org/10.1016/j.omtn.2022.03.020.

ACKNOWLEDGMENTS

This work was supported by the Natural Science Foundation of Shanghai, China (22ZR1411900), the Special Foundation for Supporting Biomedical Technology of Shanghai, China (22S11900300), and the Training Programme for the Talents of Zhongshan Hospital, Fudan University (2021ZSYQ24). We thank International Science Editing (https://www.internationalscienceediting.com) for editing the manuscript.

AUTHOR CONTRIBUTIONS

B.G., L.J., Z.M., Z.C., and W.Q. designed the research; B.G., L.J., Z.M., Z.H., J.X., L.T., Z.Y., and B.Y. carried out the experiments; B.G., L.J., Z.M., C.Z., H.Y., and V.B. analyzed and interpreted the results; B.G., L.J., and Z.M. wrote the manuscript; Z.C., W.Q., and T.L. reviewed the paper and contributed to funding acquisition.

DECLARATION OF INTERESTS

The authors declare no competing interests.

REFERENCES

1. Sung, H., Ferlay, J., Siegel, R.L., Laversanne, M., Soerjomataram, I., Jemal, A., and Bray, F. (2021). Global cancer statistics 2020: GLOBOCAN estimates of incidence and mortality worldwide for 36 cancers in 185 countries. CA Cancer J. Clin. *71*, 209–249.

2. Barta, J.A., Powell, C.A., and Wisnivesky, J.P. (2019). Global epidemiology of lung cancer. Ann. Glob. Health *85*, 8.

3. Lu, T., Yang, X., Huang, Y., Zhao, M., Li, M., Ma, K., Yin, J., Zhan, C., and Wang, Q. (2019). Trends in the incidence, treatment, and survival of patients with lung cancer in the last four decades. Cancer Manag. Res. *11*, 943–953.

4. Arbour, K.C., and Riely, G.J. (2019). Systemic therapy for locally advanced and metastatic non-small cell lung cancer: a review. JAMA *322*, 764–774.

5. Fennell, D.A., Summers, Y., Cadranel, J., Benepal, T., Christoph, D.C., Lal, R., Das, M., Maxwell, F., Visseren-Grul, C., and Ferry, D. (2016). Cisplatin in the modern era: the backbone of first-line chemotherapy for non-small cell lung cancer. Cancer Treat. Rev. *44*, 42–50.

6. Gonen, N., and Assaraf, Y.G. (2012). Antifolates in cancer therapy: structure, activity and mechanisms of drug resistance. Drug Resist. Updat. *15*, 183–210.

7. Liang, J., Lu, T., Chen, Z., Zhan, C., and Wang, Q. (2019). Mechanisms of resistance to pemetrexed in non-small cell lung cancer. Transl. Lung Cancer Res. *8*, 1107–1118.

8. Siddik, Z.H. (2003). Cisplatin: mode of cytotoxic action and molecular basis of resistance. Oncogene *22*, 7265–7279.

9. Rodrigues-Pereira, J., Kim, J.-H., Magallanes, M., Lee, D.H., Wang, J., Ganju, V., Martínez-Barrera, L., Barraclough, H., van Kooten, M., and Orlando, M. (2011). A randomized phase 3 trial comparing pemetrexed/carboplatin and docetaxel/carboplatin as first-line treatment for advanced, nonsquamous non-small cell lung cancer. J. Thorac. Oncol. *6*, 1907–1914.

10. Cheng, Y., Murakami, H., Yang, P.-C., He, J., Nakagawa, K., Kang, J.H., Kim, J.-H., Wang, X., Enatsu, S., Puri, T., et al. (2016). Randomized phase II trial of Gefitinib with and without pemetrexed as first-line therapy in patients with advanced nonsquamous non-small-cell lung cancer with activating epidermal growth factor receptor mutations. J. Clin. Oncol. *34*, 3258–3266.

11. Kreuter, M., Vansteenkiste, J., Fischer, J.R., Eberhardt, W., Zabeck, H., Kollmeier, J., Serke, M., Frickhofen, N., Reck, M., Engel-Riedel, W., et al. (2013). Randomized phase 2 trial on refinement of early-stage NSCLC adjuvant chemotherapy with cisplatin and pemetrexed versus cisplatin and vinorelbine: the TREAT study. Ann. Oncol. *24*, 986–992.

12. Buqué, A., Muhialdin, J.S., Muñoz, A., Calvo, B., Carrera, S., Aresti, U., Sancho, A., Rubio, I., and López-Vivanco, G. (2012). Molecular mechanism implicated in Pemetrexed-induced apoptosis in human melanoma cells. Mol. Cancer *11*, 25.

13. Itoh, T., Terazawa, R., Kojima, K., Nakane, K., Deguchi, T., Ando, M., Tsukamasa, Y., Ito, M., and Nozawa, Y. (2011). Cisplatin induces production of reactive oxygen species via NADPH oxidase activation in human prostate cancer cells. Free Radic. Res. *45*, 1033–1039.

14. Lu, C.-S., Lin, C.-W., Chang, Y.-H., Chen, H.-Y., Chung, W.-C., Lai, W.-Y., Ho, C.-C., Wang, T.-H., Chen, C.-Y., Yeh, C.-L., et al. (2020). Antimetabolite pemetrexed primes a favorable tumor microenvironment for immune checkpoint blockade therapy. J. Immunother. Cancer *8*, e001392.

15. Ozer, U., Barbour, K.W., Clinton, S.A., and Berger, F.G. (2015). Oxidative stress and response to thymidylate synthase-targeted antimetabolites. Mol. Pharmacol. *88*, 970–981.

16. Lei, G., Zhang, Y., Koppula, P., Liu, X., Zhang, J., Lin, S.H., Ajani, J.A., Xiao, Q., Liao, Z., Wang, H., et al. (2020). The role of ferroptosis in ionizing radiation-induced cell death and tumor suppression. Cell Res. *30*, 146–162.

17. Luo, M., Wu, L., Zhang, K., Wang, H., Zhang, T., Gutierrez, L., O'Connell, D., Zhang, P., Li, Y., Gao, T., et al. (2018). miR-137 regulates ferroptosis by targeting glutamine transporter SLC1A5 in melanoma. Cell Death Differ. *25*, 1457–1472.

18. Wang, H., Zhu, L.J., Yang, Y.C., Wang, Z.X., and Wang, R. (2014). MiR-224 promotes the chemoresistance of human lung adenocarcinoma cells to cisplatin via regulating G(1)/S transition and apoptosis by targeting p21(WAF1/CIP1). Br. J. Cancer *111*, 339–354.

19. Dweep, H., Sticht, C., Pandey, P., and Gretz, N. (2011). miRWalk–database: prediction of possible miRNA binding sites by "walking" the genes of three genomes. J. Biomed. Inform. *44*, 839–847.

20. Katsumata, S., Aokage, K., Miyoshi, T., Tane, K., Nakamura, H., Sugano, M., Kojima, M., Fujii, S., Kuwata, T., Ochiai, A., et al. (2018). Differences of tumor microenvironment between stage I lepidic-positive and lepidic-negative lung adenocarcinomas. J. Thorac. Cardiovasc. Surg. *156*, 1679–1688.e2.

21. Azizi, E., Carr, A.J., Plitas, G., Cornish, A.E., Konopacki, C., Prabhakaran, S., Nainys, J., Wu, K., Kiseliovas, V., Setty, M., et al. (2018). Single-cell map of diverse immune phenotypes in the breast tumor microenvironment. Cell *174*, 1293–1308.e36.

22. Wu, Q., Qin, S.-K., Teng, F.-M., Chen, C.-J., and Wang, R. (2010). Lobaplatin arrests cell cycle progression in human hepatocellular carcinoma cells. J. Hematol. Oncol. *3*, 43.

23. Smits, V.A., Klompmaker, R., Vallenius, T., Rijksen, G., Mäkela, T.P., and Medema, R.H. (2000). p21 inhibits Thr161 phosphorylation of Cdc2 to enforce the G2 DNA damage checkpoint. J. Biol. Chem. *275*, 30638–30643.

24. Abbas, T., and Dutta, A. (2009). p21 in cancer: intricate networks and multiple activities. Nat. Rev. Cancer *9*, 400–414.

25. Li, J., Pan, C., Boese, A.C., Kang, J., Umano, A.D., Magliocca, K.R., Yang, W., Zhang, Y., Lonial, S., Jin, L., et al. (2020). DGKA provides platinum resistance in ovarian cancer through activation of c-JUN-WEE1 signaling. Clin. Cancer Res. *26*, 3843–3855.

26. Chen, X., Kang, R., Kroemer, G., and Tang, D. (2021). Broadening horizons: the role of ferroptosis in cancer. Nat. Rev. Clin. Oncol. *18*, 280–296.

27. Stockwell, B.R., Friedmann Angeli, J.P., Bayir, H., Bush, A.I., Conrad, M., Dixon, S.J., Fulda, S., Gascon, S., Hatzios, S.K., Kagan, V.E., et al. (2017). Ferroptosis: a regulated cell death nexus linking metabolism, redox biology, and disease. Cell *171*, 273–285.

28. Dempster, J.M., Rossen, J., Kazachkova, M., Pan, J., Kugener, G., Root, D.E., and Tsherniak, A. (2019). Extracting biological insights from the project achilles genome-scale CRISPR screens in cancer cell lines. Preprint at bioRxiv. https://doi.org/10.1101/720243.

29. Hwang, J.R., Kim, W.Y., Cho, Y.-J., Ryu, J.-Y., Choi, J.-J., Jeong, S.Y., Kim, M.-S., Kim, J.H., Paik, E.S., Lee, Y.-Y., et al. (2020). Chloroquine reverses chemoresistance via upregulation of p21 and autophagy inhibition in ovarian cancer. Cell Death Dis. *11*, 1034.

30. Sun, X., Ou, Z., Chen, R., Niu, X., Chen, D., Kang, R., and Tang, D. (2016). Activation of the p62-Keap1-NRF2 pathway protects against ferroptosis in hepatocellular carcinoma cells. Hepatology *63*, 173–184.

31. Rojo de la Vega, M., Chapman, E., and Zhang, D.D. (2018). NRF2 and the hallmarks of cancer. Cancer Cell *34*, 21–43.

32. Fan, Z., Wirth, A.K., Chen, D., Wruck, C.J., Rauh, M., Buchfelder, M., and Savaskan, N. (2017). Nrf2-Keap1 pathway promotes cell proliferation and diminishes ferroptosis. Oncogenesis *6*, e371.

33. Ma, D.-B., Qin, M.-M., Shi, L., and Ding, X.-M. (2019). MicroRNA-6077 enhances the sensitivity of patients-derived lung adenocarcinoma cells to anlotinib by repressing the activation of glucose transporter 1 pathway. Cell. Signal. *64*, 109391.

34. Huang, W., Yang, Y., Wu, J., Niu, Y., Yao, Y., Zhang, J., Huang, X., Liang, S., Chen, R., Chen, S., et al. (2020). Circular RNA cESRP1 sensitises small cell lung cancer cells to chemotherapy by sponging miR-93-5p to inhibit TGF-β signalling. Cell Death Differ. *27*, 1709–1727.

35. Zeng, F., Wang, Q., Wang, S., Liang, S., Huang, W., Guo, Y., Peng, J., Li, M., Zhu, W., and Guo, L. (2020). Linc00173 promotes chemoresistance and progression of small cell lung cancer by sponging miR-218 to regulate Etk expression. Oncogene *39*, 293–307.

36. Huang, X., Li, Z., Zhang, Q., Wang, W., Li, B., Wang, L., Xu, Z., Zeng, A., Zhang, X., Zhang, X., et al. (2019). Circular RNA AKT3 upregulates PIK3R1 to enhance cisplatin resistance in gastric cancer via miR-198 suppression. Mol. Cancer *18*, 71.

37. Shalem, O., Sanjana, N.E., Hartenian, E., Shi, X., Scott, D.A., Mikkelson, T., Heckl, D., Ebert, B.L., Root, D.E., Doench, J.G., et al. (2014). Genome-scale CRISPR-Cas9 knockout screening in human cells. Science *343*, 84–87.

38. Cong, L., Ran, F.A., Cox, D., Lin, S., Barretto, R., Habib, N., Hsu, P.D., Wu, X., Jiang, W., Marraffini, L.A., et al. (2013). Multiplex genome engineering using CRISPR/Cas systems. Science *339*, 819–823.

39. Konermann, S., Brigham, M.D., Trevino, A.E., Joung, J., Abudayyeh, O.O., Barcena, C., Hsu, P.D., Habib, N., Gootenberg, J.S., Nishimasu, H., et al. (2015). Genome-scale transcriptional activation by an engineered CRISPR-Cas9 complex. Nature *517*, 583–588.

40. Zeng, H., Castillo-Cabrera, J., Manser, M., Lu, B., Yang, Z., Strande, V., Begue, D., Zamponi, R., Qiu, S., Sigoillot, F., et al. (2019). Genome-wide CRISPR screening reveals genetic modifiers of mutant EGFR dependence in human NSCLC. eLife *8*, e50223.

41. Kuang, Y., Kang, J., Li, H., Liu, B., Zhao, X., Li, L., Jin, X., and Li, Q. (2021). Multiple functions of p21 in cancer radiotherapy. J. Cancer Res. Clin. Oncol. *147*, 987–1006.

42. Karimian, A., Ahmadi, Y., and Yousefi, B. (2016). Multiple functions of p21 in cell cycle, apoptosis and transcriptional regulation after DNA damage. DNA Repair (Amst) *42*, 63–71.

43. Rong, J.-J., Hu, R., Song, X.-M., Ha, J., Lu, N., Qi, Q., Tao, L., You, Q.-D., and Guo, Q.-L. (2010). Gambogic acid triggers DNA damage signaling that induces p53/p21(Waf1/CIP1) activation through the ATR-Chk1 pathway. Cancer Lett. *296*, 55–64.

44. Chan, Q.K.Y., Lam, H.M., Ng, C.F., Lee, A.Y.Y., Chan, E.S.Y., Ng, H.K., Ho, S.M., and Lau, K.M. (2010). Activation of GPR30 inhibits the growth of prostate cancer cells through sustained activation of Erk1/2, c-jun/c-fos-dependent upregulation of p21, and induction of G(2) cell-cycle arrest. Cell Death Differ. *17*, 1511–1523.

45. Koster, R., di Pietro, A., Timmer-Bosscha, H., Gibcus, J.H., van den Berg, A., Suurmeijer, A.J., Bischoff, R., Gietema, J.A., and de Jong, S. (2010). Cytoplasmic

p21 expression levels determine cisplatin resistance in human testicular cancer. J. Clin. Invest. *120*, 3594–3605.

46. Lv, M., Zhuang, X., Zhang, Q., Cheng, Y., Wu, D., Wang, X., and Qiao, T. (2020). Acetyl-11-keto-beta-boswellic acid enhances the cisplatin sensitivity of non-small cell lung cancer cells through cell cycle arrest, apoptosis induction, and autophagy suppression via p21-dependent signaling pathway. Cell Biol. Toxicol. *37*, 209–228.
47. Ashwell, S., and Zabludoff, S. (2008). DNA damage detection and repair pathways–recent advances with inhibitors of checkpoint kinases in cancer therapy. Clin. Cancer Res. *14*, 4032–4037.
48. Abraham, R.T. (2001). Cell cycle checkpoint signaling through the ATM and ATR kinases. Genes Dev. *15*, 2177–2196.
49. Bandyopadhyay, U., Das, D., and Banerjee, R.K. (1999). Reactive oxygen species: oxidative damage and pathogenesis. Curr. Sci. *77*, 658–666.
50. Guo, J., Xu, B., Han, Q., Zhou, H., Xia, Y., Gong, C., Dai, X., Li, Z., and Wu, G. (2018). Ferroptosis: a novel anti-tumor action for cisplatin. Cancer Res. Treat. *50*, 445–460.
51. Roh, J.-L., Kim, E.H., Jang, H.J., Park, J.Y., and Shin, D. (2016). Induction of ferroptotic cell death for overcoming cisplatin resistance of head and neck cancer. Cancer Lett. *381*, 96–103.
52. Wang, Y., Zhao, G., Condello, S., Huang, H., Cardenas, H., Tanner, E.J., Wei, J., Ji, Y., Li, J., Tan, Y., et al. (2021). Frizzled-7 identifies platinum-tolerant ovarian cancer cells susceptible to ferroptosis. Cancer Res. *81*, 384–399.
53. Ma, Q. (2013). Role of nrf2 in oxidative stress and toxicity. Annu. Rev. Pharmacol. Toxicol. *53*, 401–426.
54. Cullinan, S.B., Gordan, J.D., Jin, J., Harper, J.W., and Diehl, J.A. (2004). The Keap1-BTB protein is an adaptor that bridges Nrf2 to a Cul3-based E3 ligase: oxidative stress sensing by a Cul3-Keap1 ligase. Mol. Cell. Biol. *24*, 8477–8486.
55. Tian, Y., Wu, K., Liu, Q., Han, N., Zhang, L., Chu, Q., and Chen, Y. (2016). Modification of platinum sensitivity by KEAP1/NRF2 signals in non-small cell lung cancer. J. Hematol. Oncol. *9*, 83.
56. The Cancer Genome Atlas Research Network (2014). Comprehensive molecular profiling of lung adenocarcinoma. Nature *511*, 543–550.
57. Imielinski, M., Berger, A.H., Hammerman, P.S., Hernandez, B., Pugh, T.J., Hodis, E., Cho, J., Suh, J., Capelletti, M., Sivachenko, A., et al. (2012). Mapping the hallmarks of lung adenocarcinoma with massively parallel sequencing. Cell *150*, 1107–1120.
58. Wang, S., Han, C., Liu, T., Ma, Z., Qiu, M., Wang, J., You, Q., Zheng, X., Xu, W., Xia, W., et al. (2021). FAM83H-AS1 is a noncoding oncogenic driver and therapeutic target of lung adenocarcinoma. Clin. Transl. Med. *11*, e316.
59. Zhang, L., Niu, H., Ma, J., Yuan, B.-Y., Chen, Y.-H., Zhuang, Y., Chen, G.-W., Zeng, Z.-C., and Xiang, Z.-L. (2019). The molecular mechanism of LncRNA34a-mediated regulation of bone metastasis in hepatocellular carcinoma. Mol. Cancer *18*, 120.
60. Zhao, M., Xin, X.-F., Zhang, J.-Y., Dai, W., Lv, T.-F., and Song, Y. (2020). LncRNA GMDS-AS1 inhibits lung adenocarcinoma development by regulating miR-96-5p/CYLD signaling. Cancer Med. *9*, 1196–1208.
61. Yao, Q., and Chen, T. (2020). LINC01128 regulates the development of osteosarcoma by sponging miR-299-3p to mediate MMP2 expression and activating Wnt/β-catenin signalling pathway. J. Cell. Mol. Med. *24*, 14293–14305.
62. Li, H., Tian, X., Wang, P., Hu, J., Qin, R., Xu, R., Liu, K., Hao, J., and Tian, N. (2020). LINC01128 resisted acute myeloid leukemia through regulating miR-4260/NR3C2. Cancer Biol. Ther. *21*, 615–622.
63. Bi, G., Zhu, D., Bian, Y., Huang, Y., Zhan, C., Yang, Y., and Wang, Q. (2021). Knockdown of GTF2E2 inhibits the growth and progression of lung adenocarcinoma via RPS4X *in vitro* and *in vivo*. Cancer Cell Int. *21*, 181.
64. Chen, Z., Yang, X., Bi, G., Liang, J., Hu, Z., Zhao, M., Li, M., Lu, T., Zheng, Y., Sui, Q., et al. (2020). Ligand-receptor interaction atlas within and between tumor cells and T cells in lung adenocarcinoma. Int. J. Biol. Sci. *16*, 2205–2219.
65. Tan, S.M., and Lieberman, J. (2016). Capture and identification of miRNA targets by biotin pulldown and RNA-seq. Methods Mol. Biol. *1358*, 211–228.
66. Trapnell, C., Pachter, L., and Salzberg, S.L. (2009). TopHat: discovering splice junctions with RNA-Seq. Bioinformatics *25*, 1105–1111.
67. Kim, D., Langmead, B., and Salzberg, S.L. (2015). HISAT: a fast spliced aligner with low memory requirements. Nat. Methods *12*, 357–360.
68. Bi, G., Bian, Y., Liang, J., Yin, J., Li, R., Zhao, M., Huang, Y., Lu, T., Zhan, C., Fan, H., et al. (2021). Pan-cancer characterization of metabolism-related biomarkers identifies potential therapeutic targets. J. Transl. Med. *19*, 219.
69. Bi, G., Chen, Z., Yang, X., Liang, J., Hu, Z., Bian, Y., Sui, Q., Li, R., Zhan, C., and Fan, H. (2020). Identification and validation of tumor environment phenotypes in lung adenocarcinoma by integrative genome-scale analysis. Cancer Immunol. Immunother. *69*, 1293–1305.
70. Ritchie, M.E., Phipson, B., Wu, D., Hu, Y., Law, C.W., Shi, W., and Smyth, G.K. (2015). Limma powers differential expression analyses for RNA-sequencing and microarray studies. Nucleic Acids Res. *43*, e47.
71. Subramanian, A., Tamayo, P., Mootha, V.K., Mukherjee, S., Ebert, B.L., Gillette, M.A., Paulovich, A., Pomeroy, S.L., Golub, T.R., Lander, E.S., et al. (2005). Gene set enrichment analysis: a knowledge-based approach for interpreting genome-wide expression profiles. Proc. Natl. Acad. Sci. U S A *102*, 15545–15550.
72. Chen, Z., Huang, Y., Hu, Z., Zhao, M., Li, M., Bi, G., Zheng, Y., Liang, J., Lu, T., Jiang, W., et al. (2021). Landscape and dynamics of single tumor and immune cells in early and advanced-stage lung adenocarcinoma. Clin. Transl. Med. *11*, e350.
73. Chen, Z., Zhao, M., Liang, J., Hu, Z., Huang, Y., Li, M., Pang, Y., Lu, T., Sui, Q., Zhan, C., et al. (2021). Dissecting the single-cell transcriptome network underlying esophagus non-malignant tissues and esophageal squamous cell carcinoma. EBioMedicine *69*, 103459.
74. Macosko, E.Z., Basu, A., Satija, R., Nemesh, J., Shekhar, K., Goldman, M., Tirosh, I., Bialas, A.R., Kamitaki, N., Martersteck, E.M., et al. (2015). Highly parallel genome-wide expression profiling of individual cells using nanoliter droplets. Cell *161*, 1202–1214.
75. Ran, F.A., Hsu, P.D., Wright, J., Agarwala, V., Scott, D.A., and Zhang, F. (2013). Genome engineering using the CRISPR-Cas9 system. Nat. Protoc. *8*, 2281–2308.
76. Maruyama, T., Dougan, S.K., Truttmann, M.C., Bilate, A.M., Ingram, J.R., and Ploegh, H.L. (2015). Increasing the efficiency of precise genome editing with CRISPR-Cas9 by inhibition of nonhomologous end joining. Nat. Biotechnol. *33*, 538–542.
77. Jackson, S.P. (2002). Sensing and repairing DNA double-strand breaks. Carcinogenesis *23*, 687–696.
78. Lieber, M.R., Ma, Y., Pannicke, U., and Schwarz, K. (2003). Mechanism and regulation of human non-homologous DNA end-joining. Nat. Rev. Mol. Cell Biol. *4*, 712–720.

OMTN, Volume 28

Supplemental information

miR-6077 promotes cisplatin/pemetrexed resistance in lung adenocarcinoma via CDKN1A/ cell cycle arrest and KEAP1/ferroptosis pathways

Guoshu Bi, Jiaqi Liang, Mengnan Zhao, Huan Zhang, Xing Jin, Tao Lu, Yuansheng Zheng, Yunyi Bian, Zhencong Chen, Yiwei Huang, Valeria Besskaya, Cheng Zhan, Qun Wang, and Lijie Tan

Supplementary Table 1. Oligonucleotides

Name	Source	Sequence
miR-NC1 mimic<br>(micrON mimic NC#19)	RiboBio	Sense: 5'-3':<br>UUUGUACUACACAAAAGUACUG<br>Antisense: 3'-5':<br>AAACAUGAUGUGUUUUCAUGAC
miR-NC2 mimic<br>(micrON mimic NC#22)	RiboBio	Sense: 5'-3':<br>UCACAACCUCCUAGAAAGAGUAGA<br>Antisense: 3'-5':<br>AGUGUUGGAGGAUCUUUCUCAUCU
hsa-miR-6077 mimic<br>(Purchased from)	RiboBio	Sense: 5'-3':<br>GGGAAGAGCUGUACGGCCUUC<br>Antisense: 3'-5':<br>CCCUUCUCGACAUGCCGGAAG
hsa-miR-3678-3p mimic	RiboBio	Sense: 5'-3':<br>CUGCAGAGUUUGUACGGACCGG<br>Antisense: 3'-5':<br>GACGUCUCAAACAUGCCUGGCC
hsa-miR-3678-5p mimic	RiboBio	Sense: 5'-3':<br>UCCGUACAAACUCUGCUGUG<br>Antisense: 3'-5':<br>AGGCAUGUUUGAGACGACAC
hsa-miR-7702 mimic	RiboBio	Sense: 5'-3':<br>CUUAGACUGCCAGACUCCCUGA<br>Antisense: 3'-5':<br>GAAUCUGACGGUCUGAGGGACU
hsa-miR-6855-5p mimic	RiboBio	Sense: 5'-3':<br>UUGGGGUUUGGGGUGCAGACAUUG<br>C<br>Antisense: 3'-5':<br>AACCCCAAACCCCACGUCUGUAACG
hsa-miR-548ak mimic	RiboBio	Sense: 5'-3':<br>AAAAGUAACUGCGGUUUUUGA<br>Antisense: 3'-5':<br>UUUUCAUUGACGCCAAAAACU
miR-NC1 inhibitor<br>(micrOFF NC#19)	RiboBio	Antisense: 3'-5':<br>AAACAUGAUGUGUUUUCAUGAC
miR-NC2 inhibitor<br>(micrOFF NC#22)	RiboBio	Antisense: 3'-5':<br>AGUGUUGGAGGAUCUUUCUCAUCU
hsa-miR-6077 inhibitor	RiboBio	Sense: 5'-3':<br>GGGAAGAGCUGUACGGCCUUC<br>Antisense: 3'-5':<br>CCCUUCUCGACAUGCCGGAAG

Supplementary Table 2. Primers used for PCR and qPCR

Gene	Source	Sequence
CDKN1A	Sangon Biotech	F: TGTCCGTCAGAACCCATGC<br>R: AAAGTCGAAGTTCCATCGCTC
KEAP1 (qPCR)	Sangon Biotech	F: CTGGAGGATCATACCAAGCAGG<br>R: GGATACCCTCAATGGACACCAC
KEAP1 (PCR)	Sangon Biotech	F: ACTCGTTGACGCCGAACTTCC<br>R: GGGTTGTAAGCCTCCAGGTAGC
PTGS2	Sangon Biotech	F: CTGGCGCTCAGCCATACAG<br>R: CGCACTTATACTGGTCAAATCCC
GAPDH	Sangon Biotech	F: AGAAGGCTGGGGCTCATTTG<br>R: AGGGGCCATCCACAGTCTTC
GMDS-AS1	Sangon Biotech	F: AATGCTTTGAGGCCAAGCTA<br>R: TGGGTTCATAAGGGTTGCAT
LINC01128	Sangon Biotech	F: CAGAGGAGCTACGAAGGGAG<br>R: CTGTGGAATCGCTTGGTACG
LINC00174	Sangon Biotech	F: AACCTCTCAGGCTCAAGCAGTC<br>R: GGTCACTTCGTTTCGCAGTCAG
LINC01278	Sangon Biotech	F: AAGAAAGCTTCTGTGCCCCC<br>R: AGCTCAGCAGGCAGAATTGA
RALY-AS1	Sangon Biotech	F: AAGGAACGTGCCACCTAGAC<br>R: GGGGAAGCGATGTGAGTCAT
U6	TIANGEN	F: CTCGCTTCGGCAGCACA<br>R: AACGCTTCACGAATTTGCGT
miR-6077	TIANGEN	F: AACAAGGGGAAGAGCTGTACGG<br>R: CAGTGCAGGGTCCGAGGT

Supplementary Table 3. Characteristics of the 31 NSCLC patients included in this study

ID	Age	Gender	T stage	N stage	Location	Cohort	Group
Patient 1	59	M	2	2	R	A	Resistant
Patient 2	37	M	2	0	R	A	Resistant
Patient 3	58	M	3	0	L	A	Sensitive
Patient 4	49	M	2	0	R	A	Resistant
Patient 5	53	M	1	0	R	A	Sensitive
Patient 6	67	M	2	0	R	A	Sensitive
Patient 7	68	M	2	2	R	A	Resistant
Patient 8	60	W	2	2	L	A	Sensitive
Patient 9	61	M	2	0	R	A	Sensitive
Patient 10	65	M	3	2	L	A	Resistant
Patient 11	59	M	2	0	R	A	Resistant
Patient 12	56	M	2	1	L	A	Sensitive
Patient 13	54	M	2	1	R	A	Sensitive
Patient 14	57	W	3	0	L	A	Sensitive
Patient 15	62	W	2	1	L	A	Resistant
Patient 16	56	M	2	1	R	A	Resistant
Patient 17	62	W	3	1	R	A	Sensitive
Patient 18	59	M	2	1	R	A	Sensitive
Patient 19	60	W	2	0	L	A	Resistant
Patient 20	63	W	3	0	R	A	Sensitive
Patient 21	70	M	3	1	R	B	Treated
Patient 22	60	W	2	2	R	B	Treated
Patient 23	50	M	2	3	L	B	Treated
Patient 24	58	M	1	2	R	B	Treated
Patient 25	66	W	2	2	R	B	Untreated
Patient 26	52	W	2	1	L	B	Untreated
Patient 27	74	M	2	2	R	B	Untreated
Patient 28	47	W	3	2	R	B	Untreated
Patient 29	82	M	2	0	L	PDX #1	Untreated
Patient 30	60	M	2	1	L	PDX #2	Untreated
Patient 31	63	W	2	1	R	PDX #3	Untreated

Supplementary Table 4. Antibodies and dyes used for western blotting and flow cytometry

Protein	Source	Dilution
CDKN1A	Abways, #CY5088	1:1000
KEAP1	Absin, #abs131362	1:1000
TUBULIN	Beyotimes, #AF1216	1:1000
MAP3K1	Santa Cruz Biotechnology (USA), #sc-17820	1:500
FOXP4	Santa Cruz Biotechnology (USA), #sc-390892	1:1000
CREN1	Abways, #CY5426	1:1000
KLF6	Santa Cruz Biotechnology (USA), #sc-365633	1:1000
KLF7	Sigma-Aldrich (USA), #HPA030490	1:2000
VEGFA	Abways, #CY5096	1:500
ACTIN	Beyotime, #AF0003	1:1000
CDK1	Abways, #AB3187	1:100
CDK1-Thr161	Abways, #CY6096	1:500
NRF2	Absin, #abs120634	1:500
SLC7A11	Affinity, #DF12509	1:1000
NQO1	Abways, #CY6710	1:1000
AGO2	Abways, #CY6652	1:500
Dyes	Source	Conjugation
Zombie Aqua Fixable Viability Kit	BioLegend (San Diego, USA), #423101	BV510
EPCAM	BioLegend, #369809	APC
CD45	BioLegend, #368531	PE/Cyanine7
FOLR1	R & D Systems (Minneapolis, USA), #FAB5646P	PE

Supplementary Table 5. Sequences of sgRNA, repair template, and the siRNAs used in HDR

Name	Source	Sequence
sgRNA	Sangon Biotech	CTGTCGGAAGTAGCAGCCCG
Repair template	Sangon Biotech	TGCAGATGCAGCTGCAGAAGTGCG<br>AGATCCTGCAGTCCGACTCCCGCTG<br>CAAGGACTACCTGGTCAAGATCTTC<br>GAGGAGCTCACCCTGCACAAGCCC<br>ACGCAGGTGATGCCCTGCCGGGCC<br>CCTAAGGTGGGCCGCCTGATCTACA<br>CCGCGGGAGGATATTTCAGGCAAT<br>CGCTCAGCTACCTGGAGGCTTACA<br>ACCCCAGTGACGGCACCTGGCTCC<br>GGTTGGCGGACCTGCAGGTGCCGC<br>GGAGCGGCCTGGCCGGCTGCGTGG<br>TGGGCGGGCTGTTGTACGCCGTGG<br>GCGGCAGGAACAACTCGCCCGAC<br>GGCAAC
siKu70-1	RiboBio	UUCAGGUGACUCCUCCAGGTT
siKu70-2	RiboBio	UUCUCUUGGUAACUUUCCCTT
siXRCC4-1	RiboBio	AUAUGUUGGUGAACUGAGATT
siXRCC4-2	RiboBio	UCUUGGGACAGAACCUAAATT
siLig4-1	RiboBio	CGACCUUUUAGACUCAAUUTT
siLig4-2	RiboBio	GCUAGAUGGUGAACGUAUGTT

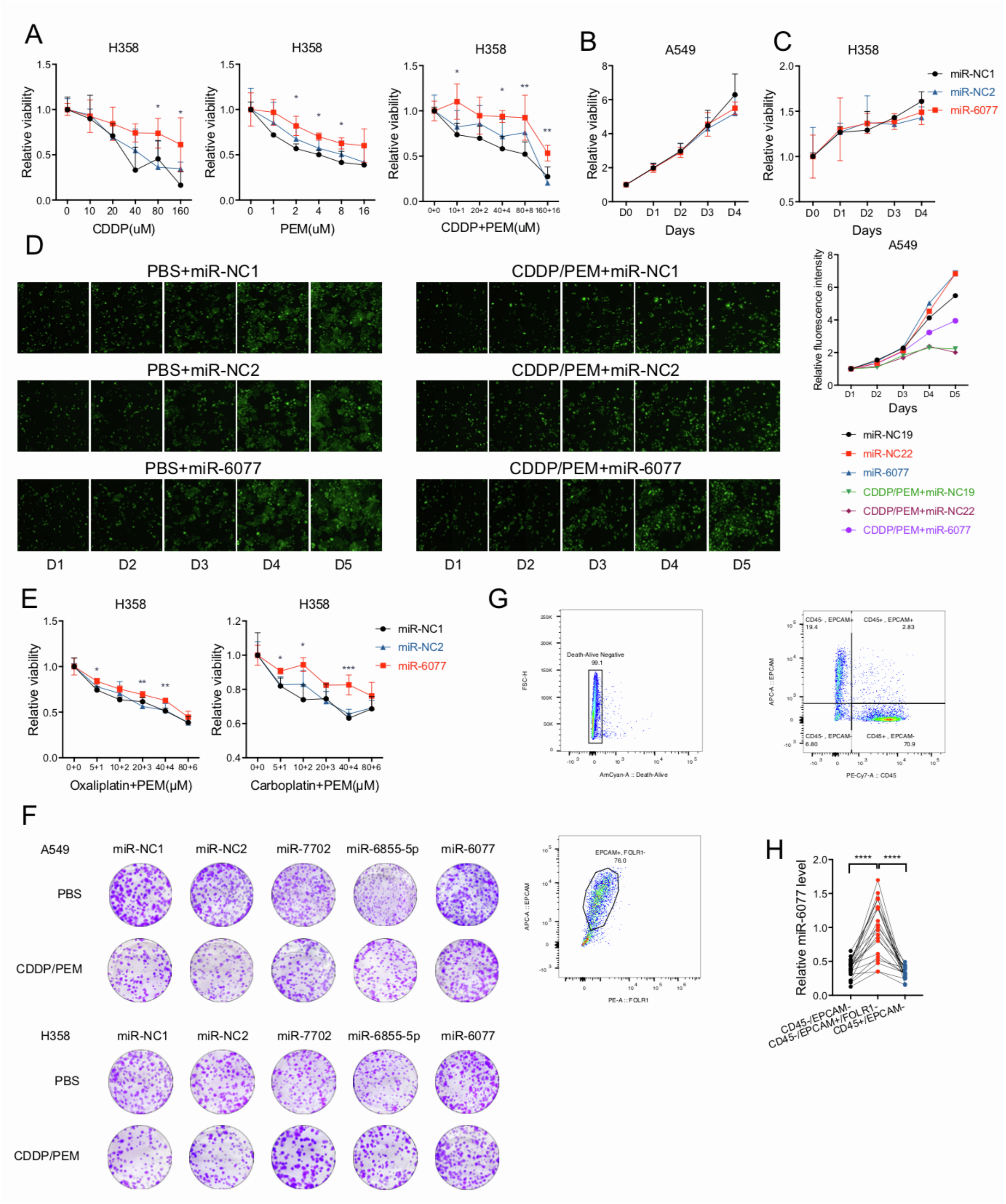

Supplementary Figure 1. Ectopic overexpression of miR-6077 confers resistance to CDDP/PEM in LUAD. **(A)** Dose-toxicity curves showing the cell viability of H358 cells transfected with miR-NC1, miR-NC2, and miR-6077 upon CDDP or PEM treatments at the indicated concentrations for 48 h respectively. **(B, C)** Growth curves showing the proliferating rates of A549 **(B)** and H358 cells **(C)** transfected with miR-NC1, miR-NC2, and miR-6077. **(D)** High-Content Analysis and quantification of GFP-

overexpressing A549 cells transfected with miR-NC1, miR-NC2, and miR-6077 shows the dynamic change of cell proliferation in five days in the presence of PBS or CDDP (10 μM)/PEM (1 μM). (bottom). **(E)** Viability of H358 cells transfected with miR-NC1, miR-NC2, and miR-6077 upon treatments with PEM and oxaliplatin or carboplatin at the indicated concentrations for 48 h respectively. **(F)** Colony formation ability of A549 and H358 cells transfected with different miRNAs upon treatments with PBS or CDDP /PEM for 14 days. **(G)** LUAD patients' surgical specimen were dissected and lymphocytes (CD45+/EPCAM-), tumor cells (CD45-/EPCAM+/FOLR-), other non-tumoral cells (CD45-/EPCAM-), and were identified and sorted by flow cytometry according to the expression of marker genes. **(H)** Relative expression levels of miR-6077 in different cell types. Data are presented as mean ± s.d., n = 3 independent repeats. Unpaired, two-tailed t-test; *$p < 0.05$, **$p < 0.01$, ***$p < 0.001$, ****$p < 0.0001$, ns, not significant.

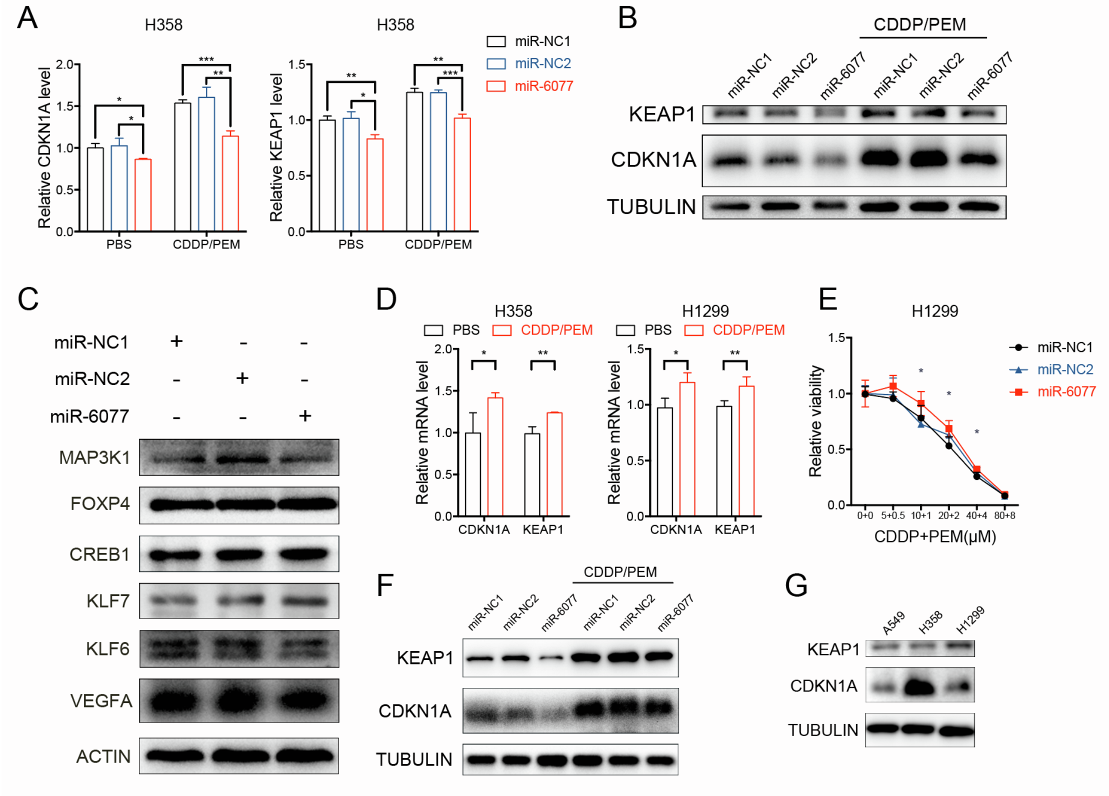

Supplementary Figure 2. CDKN1A and KEAP1 are direct targets of miR-6077 in different LUAD cell lines. **(A, B)** qRT-PCR and western blotting assays showing the mRNA **(A)** and protein **(B)** levels of CDKN1A and KEAP1 in H358 pre-transfected with miR-NC1, miR-NC2, and miR-6077 after PBS or CDDP (20 μM)/PEM (2 μM) treatments for 48 h. **(C)** The protein levels of other six potential downstream targets of miR-6077 in A549 cells transfected with miR-NC1, miR-NC2, and miR-6077. **(D)** qRT-PCR showing the relative expression levels of CDKN1A and KEAP1 in H358 and H1299 cells upon treatments with PBS or CDDP (20 μM)/PEM (2 μM) for 48 h. **(E)** Dose-toxicity curves showing the cell viability of H1299 transfected with miR-NC1, miR-NC2, and miR-6077 upon CDDP/PEM treatments at the indicated concentrations for 48 h. **(F)** The protein levels of CDKN1A and KEAP1 in H1299 transfected with miR-NC1, miR-NC2, and miR-6077 upon treatments with PBS or CDDP (20 μM)/PEM

(2 μM) for 48 h. Data are presented as mean ± s.d., n = 3 independent repeats. Unpaired, two-tailed t-test; *p < 0.05, **p < 0.01, ***p < 0.001, ****p < 0.0001, ns, not significant.

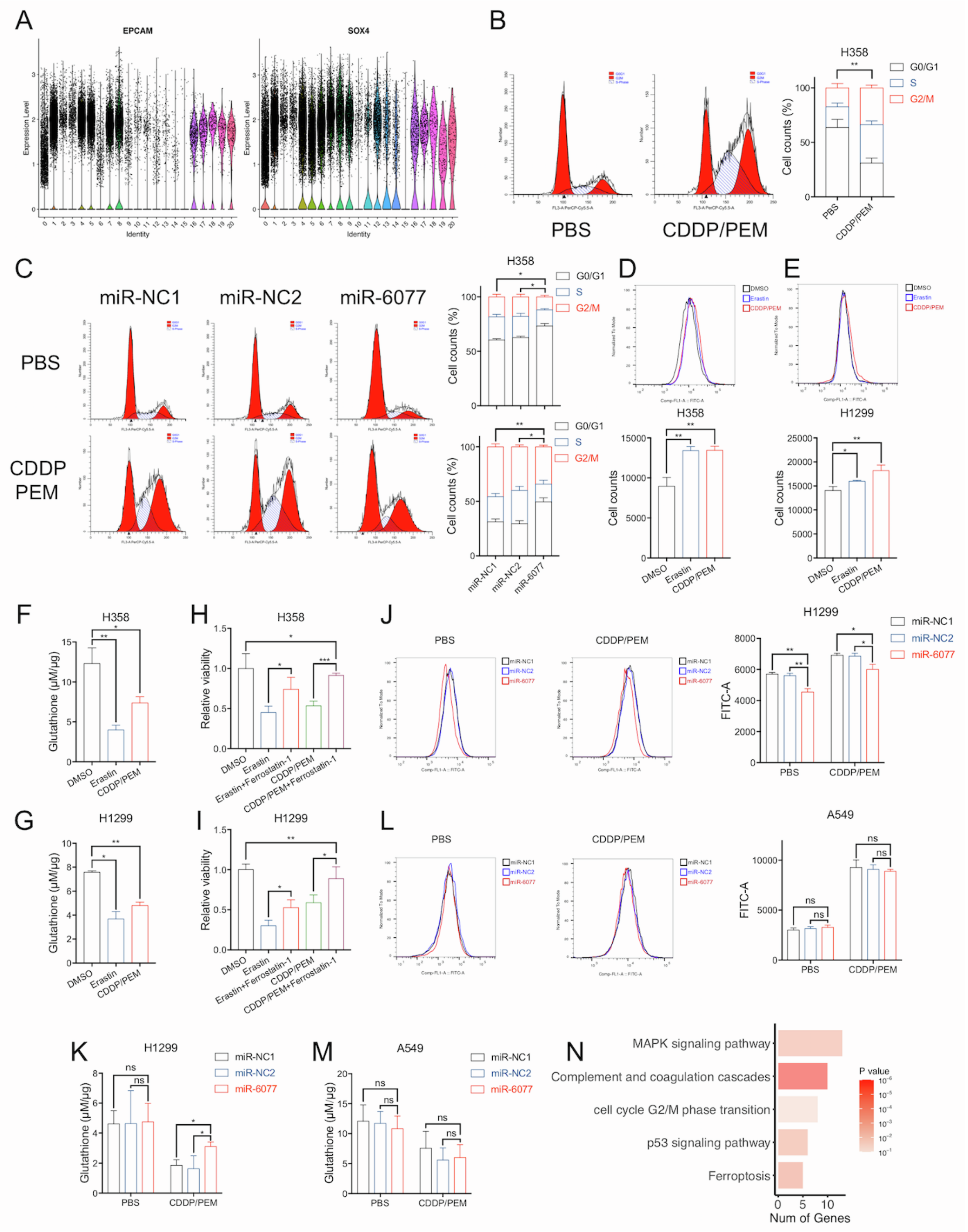

Supplementary Figure 3. miR-6077 protects LUAD cells from cell cycle arrest and ferroptosis induced by CDDP/PEM treatment in different LUAD cell lines. **(A)** Violin plots displaying the expression of well-known representative markers of tumor cells in LUAD patients' surgical sample. The y-axis shows the normalized read count. **(B)** Cell cycle analyses of H358 cells treated with PBS or CDDP (20 μM)/PEM (2 μM) for 48h.

(C) Cell cycle analyses of H358 transfected with miR-NC1, miR-NC2, and miR-6077 upon treatments with PBS or CDDP (20 μM)/PEM (2 μM) for 48 h. **(D, E)** Lipid peroxidation in H358 **(D)** and H1299 **(E)** cells treated with PBS or CDDP (20 μM)/PEM (2 μM) for 48 h. **(F, G)** Relative GLUTATHIONE levels in H358 **(F)** and H1299 **(G)** cells treated with PBS or CDDP (20 μM)/PEM (2 μM) for 48h. **(H, I)** Viability of H358 **(H)** and H1299 **(I)** cells treated as indicated for 48 h. **(J, L)** Lipid peroxidation in H1299 **(J)** and A549 **(L)** transfected with miR-NC1, miR-NC2, and miR-6077 upon treatments with PBS or CDDP (20 μM)/PEM (2 μM) for 48h. **(K, M)** Relative GLUTATHIONE levels in H1299 **(K)** and A549 **(M)** transfected with miR-NC1, miR-NC2, and miR-6077 upon treatments with PBS or CDDP (20 μM)/PEM (2 μM) for 48 h. **(N)** Functional enrichment analysis of the differentially expressed genes between A549 cells transfected with miR-NC and miR-6077 upon CDDP/PEM treatment for 48 h. Data are presented as mean ± s.d., n = 3 independent repeats. Unpaired, two-tailed t-test; *p < 0.05, **p < 0.01, ***p < 0.001, ****p < 0.0001, ns, not significant.

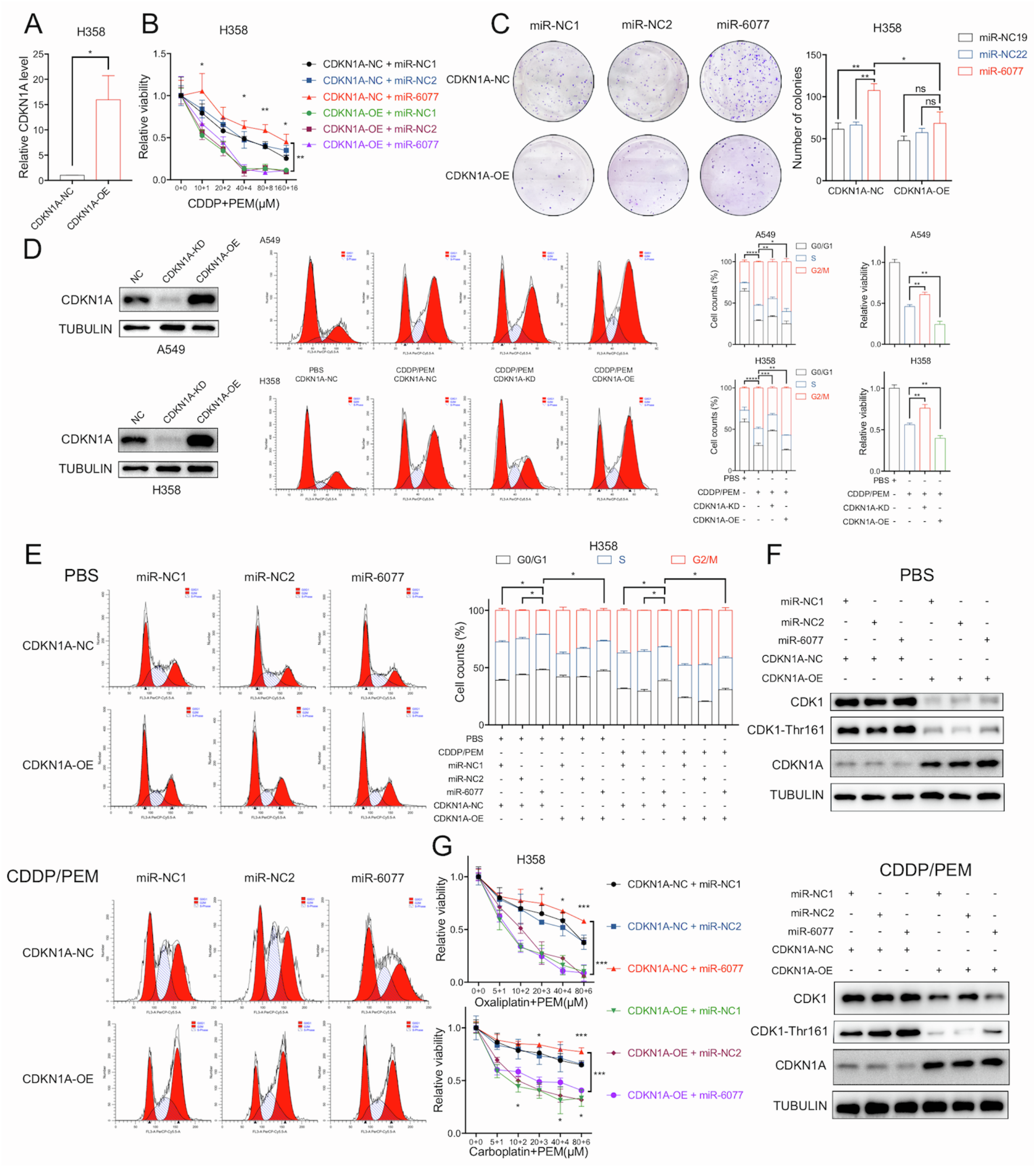

Supplementary Figure 4. CDKN1A overexpression restores cell cycle arrest in different LUAD cells. **(A)** qRT-PCR assay confirming the ectopic overexpression of CDKN1A in H358 cells. **(B)** Dose-toxicity curves showing the viability of H358 cells (with or without CDKN1A overexpression) transfected with miR-NC1, miR-NC2, and miR-6077 upon CDDP/PEM treatments at the indicated concentrations for 48 h. **(C, E, F)** Colony formation ability **(C)**, cell cycle proportion **(E)**, and the protein levels of CDKN1A and its downstream cell cycle regulators **(F)** in H358 cells (with or without

CDKN1A overexpression) transfected with miR-NC1, miR-NC2, and miR-6077 upon treatments with PBS or CDDP (20 μM)/PEM (2 μM) for 14 days **(C)** and 48 h **(E, F)** respectively. **(D)** The impact of CDKN1A-KD and OE on cell cycle proportion and cell's sensitivity to CDDP/PEM in A549 (10 μM CDDP /1 μM PEM) and H358 (20 μM CDDP /2 μM PEM). **(G)** Dose-toxicity curves showing the viability of H358 cells (with or without CDKN1A overexpression) transfected with miR-NC1, miR-NC2, and miR-6077 upon treatments with PEM and oxaliplatin or carboplatin treatments at the indicated concentrations for 48 h. Data are presented as mean ± s.d., n = 3 independent repeats. Unpaired, two-tailed t-test; $*p < 0.05$, $**p < 0.01$, $***p < 0.001$, $****p < 0.0001$, ns, not significant.

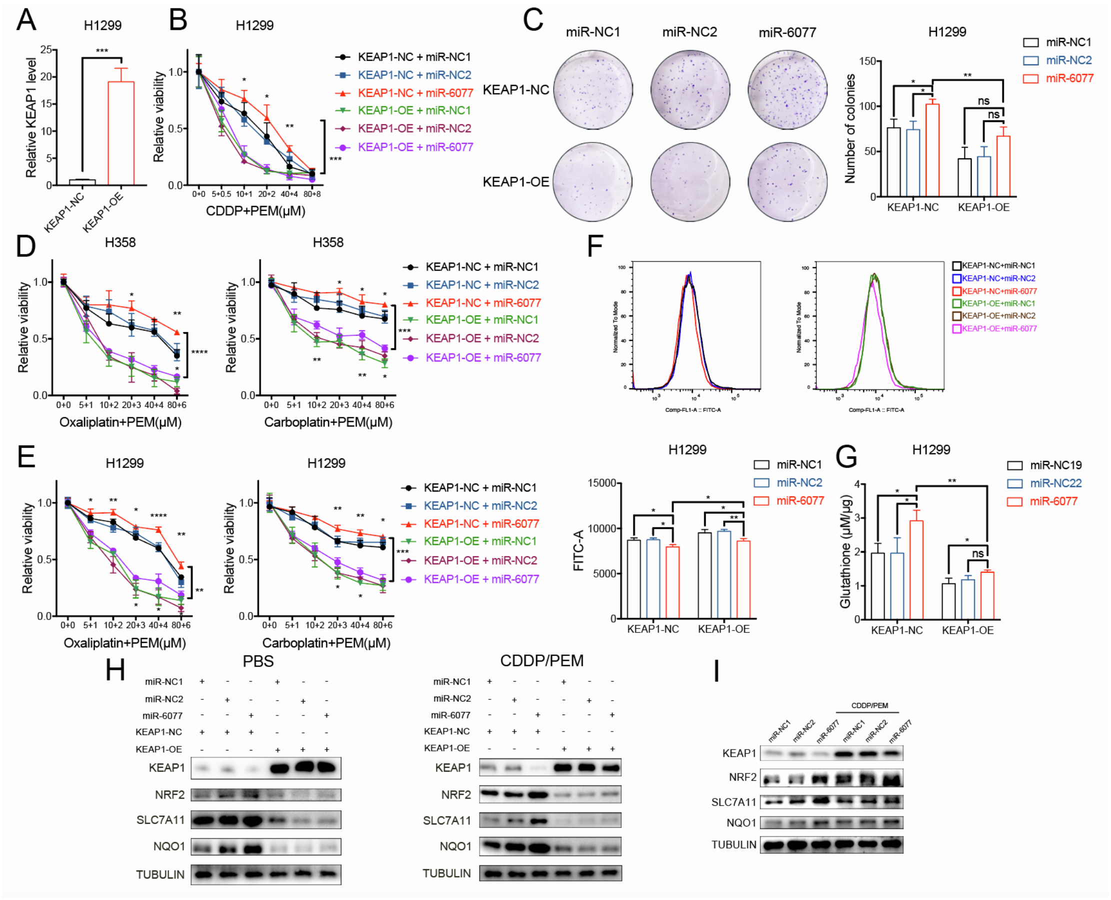

Supplementary Figure 5. KEAP1 overexpression restores ferroptosis in different LUAD cells. **(A)** qRT-PCR assay confirming the ectopic overexpression of KEAP1 in H1299 cells. **(B)** Dose-toxicity curves showing the viability of H1299 cells (with or without KEAP1 overexpression) transfected with miR-NC1, miR-NC2, and miR-6077 upon CDDP/PEM treatments at the indicated concentrations for 48 h. **(C, F, G, H)** Colony formation ability **(C)**, lipid peroxidation **(F)**, relative glutathione levels **(G)**, and the protein levels of KEAP1 and its downstream ferroptosis regulators **(H)** in H1299 cells (with or without KEAP1 overexpression) transfected with miR-NC1, miR-NC2, and miR-6077 upon treatments with PBS or CDDP (20 μM)/PEM (2 μM) for 14 days. **(C)** and 48 h **(F, G, H)** respectively. **(D, E)** Dose-toxicity curves showing the viability of H358 **(D)** and H1299 **(E)** cells (with or without KEAP1 overexpression)

transfected with miR-NC1, miR-NC2, and miR-6077 upon treatments with PEM and oxaliplatin or carboplatin at the indicated concentrations for 48 h. **(I)** Levels of ferroptosis-related proteins in A549-HDR cells with or without miR-6077 overexpression upon treatments with PBS or CDDP (10 μM)/PEM (1 μM) for 48h. Data are presented as mean ± s.d., n = 3 independent repeats. Unpaired, two-tailed t-test; *p < 0.05, **p < 0.01, ***p < 0.001, ****p < 0.0001, ns, not significant.

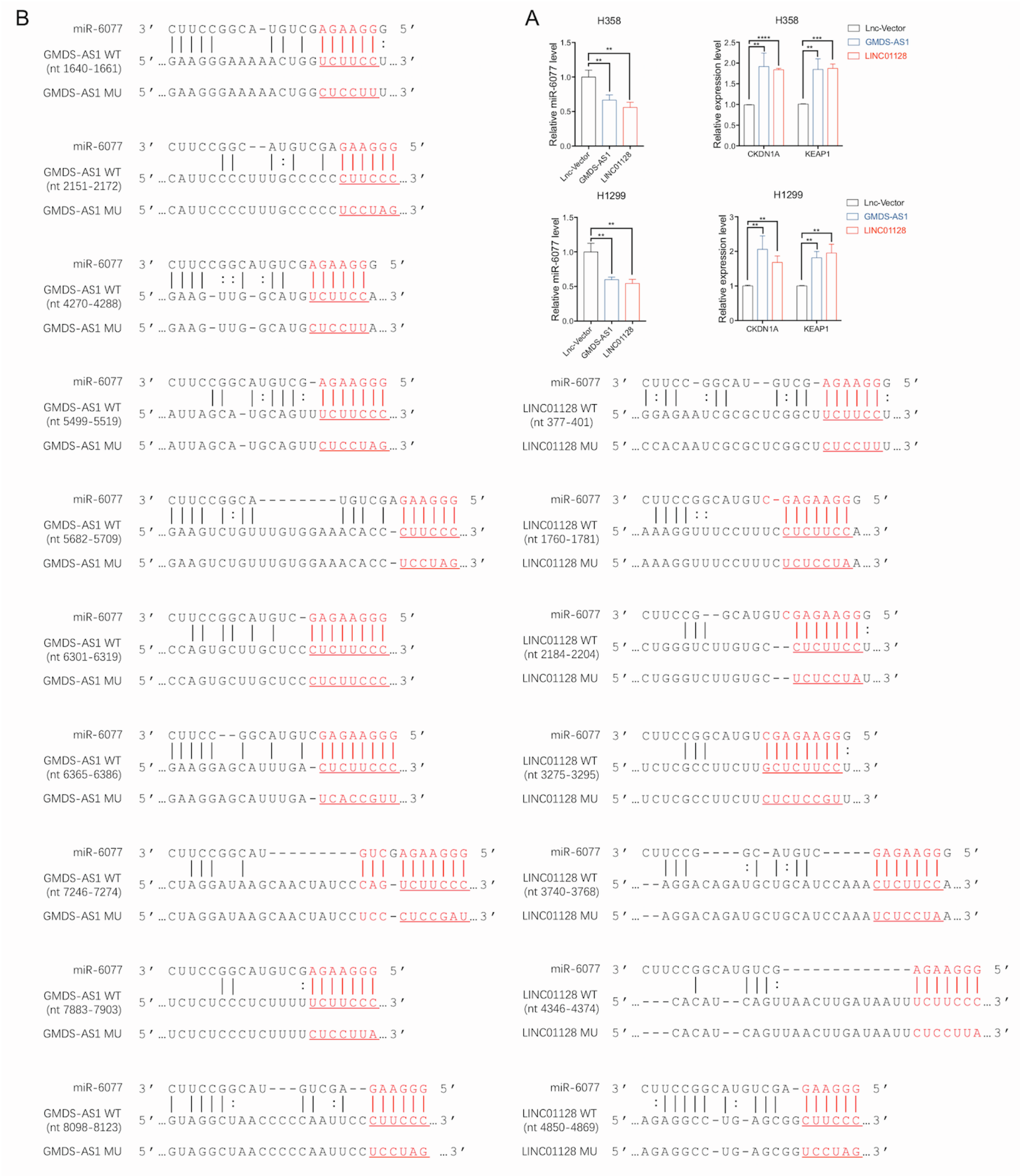

Supplementary Figure 6. Targeting relationship between miR-6077 and GMDS-AS1 /LINC01128. **(A)** Relative expression levels of miR-6077, CDKN1A, and KEAP1 in H358 and H1299 with or without GMDS-AS1/LINC01128 overexpression. **(B)** The predicted target sites of miR-6077 in GMDS-AS1 and LINC01128. Normal and mutant seed regions are highlighted and underlined.

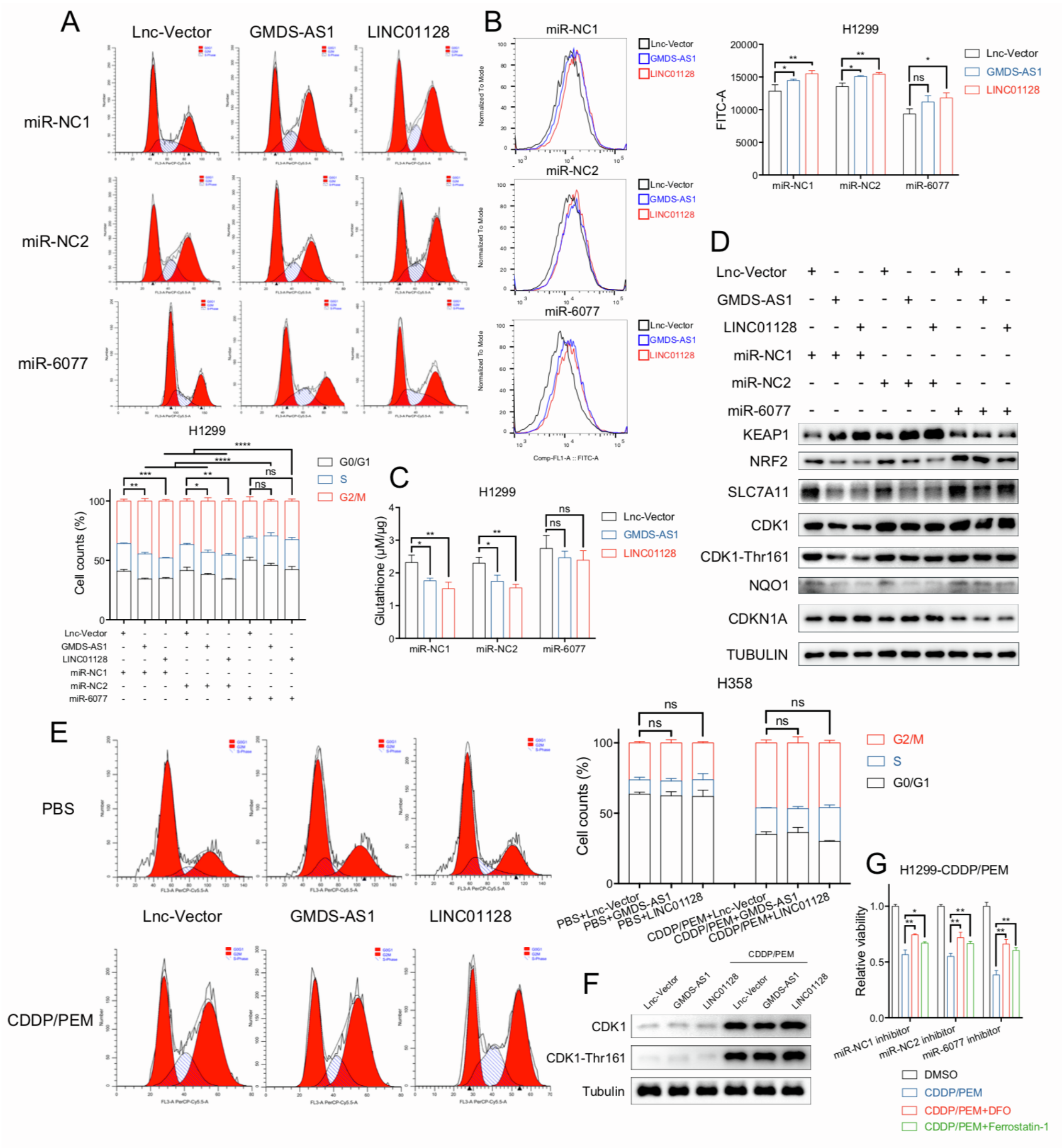

Supplementary Figure 7. Overexpression of GMDS-AS1 and LINC01128 augment the magnitude of CDDP/PEM caused G2/M arrest and lipid peroxidation by inhibiting miR-6077 in different LUAD cells. **(A, B, C, D)** Cell cycle proportion **(A)**, lipid peroxidation levels **(B)**, glutathione levels **(C)**, and the protein levels of CDKN1A/KEAP1 and their downstream molecules **(D)** in GMDS-AS1/LINC01128 overexpressed H1299 cells transfected with miR-NC1, miR-NC2, and miR-6077 upon treatments with PBS or CDDP (20 μM)/PEM (2 μM) for 48 h. **(E, F)** Cell cycle proportion **(E)** and the protein levels of CDK1 **(F)** in GMDS-AS1/LINC01128

overexpressed H358 cells transfected with miR-6077 inhibitor upon treatments with PBS or CDDP (20 μM)/PEM (2 μM) for 48 h. **(G)** Viability of H1299-CDDP/PEM-resistant cells transfected with miR-NC1 inhibitor, miR-NC2 inhibitor, and miR-6077 inhibitor upon treatments as indicated for 48h.
